# Sulfonyl Nitrene and Amidyl Radical: Structure and Reactivity

**DOI:** 10.1002/chem.202104493

**Published:** 2022-04-05

**Authors:** Jan Zelenka, Aleksandr Pereverzev, Ullrich Jahn, Jana Roithová

**Affiliations:** ^1^ Department of Spectroscopy and Catalysis Institute for Molecules and Materials Radboud University Nijmegen, Heyendaalseweg 135 6525 AJ Nijmegen The Netherlands; ^2^ Institute of Organic Chemistry and Biochemistry of the Czech Academy of Sciences Flemingovo náměstí 2 16610 Prague 6 Czech Republic

**Keywords:** amidyl radical, ion spectroscopy, nitrene, photocatalysis, reaction mechanisms

## Abstract

Photocatalytic generation of nitrenes and radicals can be used to tune or even control their reactivity. Photocatalytic activation of sulfonyl azides leads to the elimination of N_2_ and the resulting reactive species initiate C−H activations and amide formation reactions. Here, we present reactive radicals that are generated from sulfonyl azides: sulfonyl nitrene radical anion, sulfonyl nitrene and sulfonyl amidyl radical, and test their gas phase reactivity in C−H activation reactions. The sulfonyl nitrene radical anion is the least reactive and its reactivity is governed by the proton coupled electron transfer mechanism. In contrast, sulfonyl nitrene and sulfonyl amidyl radicals react via hydrogen atom transfer pathways. These reactivities and detailed characterization of the radicals with vibrational spectroscopy and with DFT calculations provide information necessary for taking control over the reactivity of these intermediates.

## Graphical abstract

**Photochemical activation of sulfonyl azides** leads to generation of reactive radicals that activate aliphatic C−H bonds. This study compares intrinsic reactivities of triplet sulfonyl nitrene, sulfonyl amidyl radicals and sulfonyl nitrene radical anions in the gas phase towards a series of hydrocarbons and correlates it to their structures investigated by ion spectroscopy. The most reactive triplet nitrene abstracts a hydrogen atom from ethane.

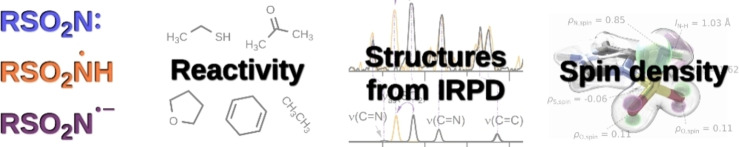

## Introduction

Sulfonyl nitrenes have been attracting chemists’ attention for more than a half of a century because of their ability to form C−N bonds.[^1^, ^2^] It has been shown that such nitrenes can react with thiols, phosphanes, amines and even aliphatic hydrocarbons.^[3]^ The reaction mechanisms were studied by electron paramagnetic resonance (EPR) spectroscopy and by the use of radical scavengers.^[4]^ Characterization of sulfonyl nitrenes relied on their photogeneration in cryo‐cooled matrixes and the trapped sulfonyl nitrenes were studied by EPR, ultraviolet and infrared spectroscopy.[^5^, ^6a^] These experiments revealed possibility of their pseudo‐Curtius rearrangement.[^5^, ^7^] UV‐pump IR‐probe experiments in solution showed that sulfonyl nitrenes have the triplet ground state and that the excited singlet state decays on the sub‐ns timescale.^[8]^ Reactions of sulfonyl nitrenes with unsaturated systems have been explored in silico.^[9]^ However, experimental studies of the reactivity of the isolated sulfonyl nitrenes have not been presented so far.

**Figure 1:**
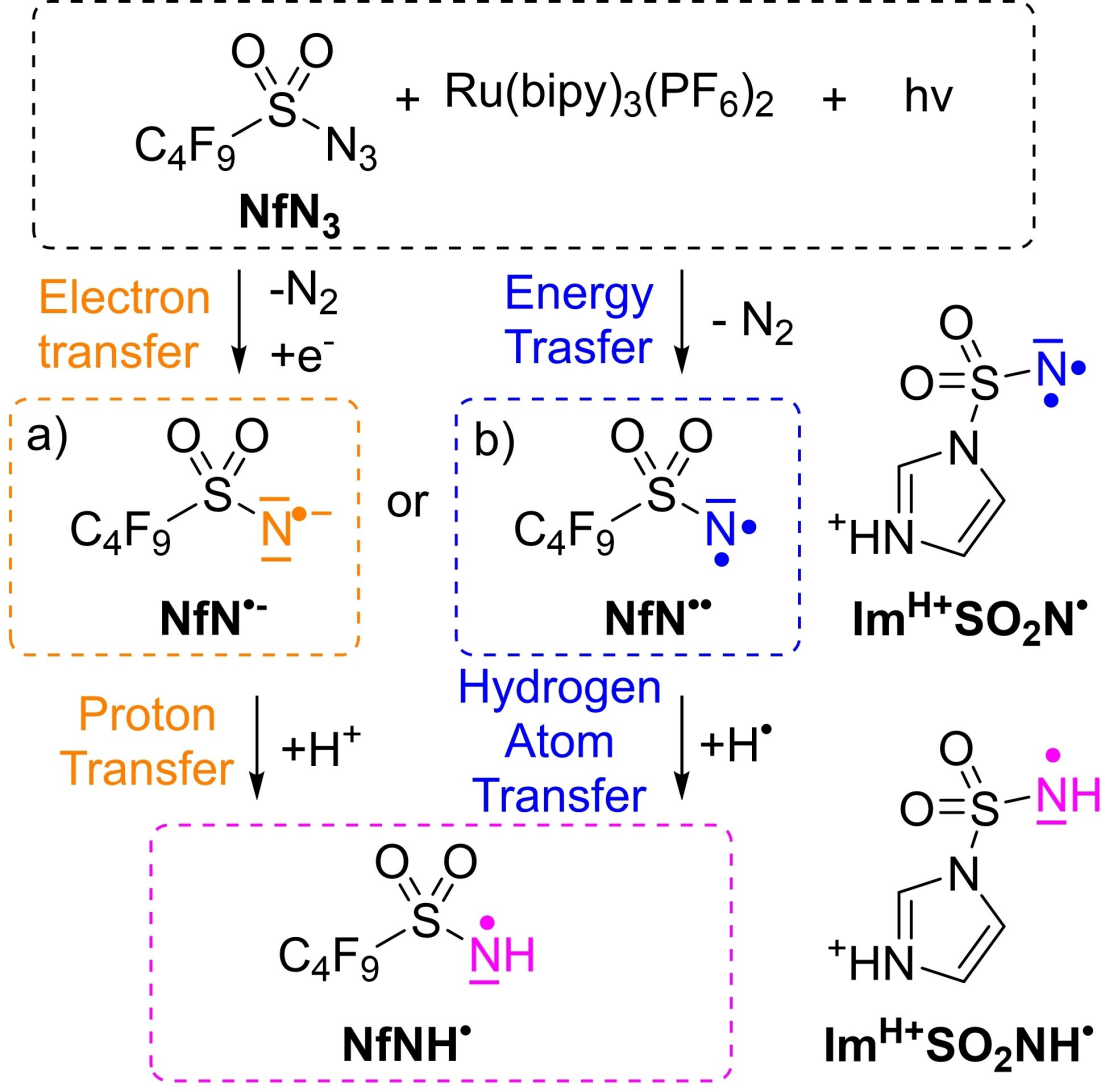
Possible products of Ru(bipy)_3_^2+^ photoreaction with nonaflyl azide (NfN_3_). a) Electron transfer leading to nonaflyl nitrene radical‐anion (NfN^.−^). b) Energy transfer leading to triplet nonaflyl‐nitrene (NfN^..^). Both pathways can lead to the nonaflyl amidyl radical (NfNH⋅). Imidazoyl sulfonyl nitrene and amidyl radical (Im^H+^SO_2_N^..^ and Im^H+^SO_2_NH⋅) are charge‐tagged analogues of the neutral intermediates.

Activation of nonafluorobutanesulfonyl azide (nonaflyl azide, NfN_3_) by photoexcited Ru(bipy)_3_(PF_6_)_2_ (bipy=2,2′‐bipyridine) leads to nitrene‐based chemistry (Figure 1).^[10]^ Two reaction pathways can be considered: a) the excited ruthenium catalyst transfers an electron to the nonaflyl azide, leading to the formation of a NfN^.−^ radical anion (Figure 1a)^[11]^ or b) energy transfer from the excited ruthenium catalyst to the azide, leading to a neutral nitrene (NfN^..^, Figure 1b). In order to understand this chemistry, we investigate the intrinsic reactivities of the bare, isolated intermediates (sulfonyl nitrenes, nitrene radical anions and amidyl radicals) acting on these two pathways.

## Results and Discussion

**Figure 2:**
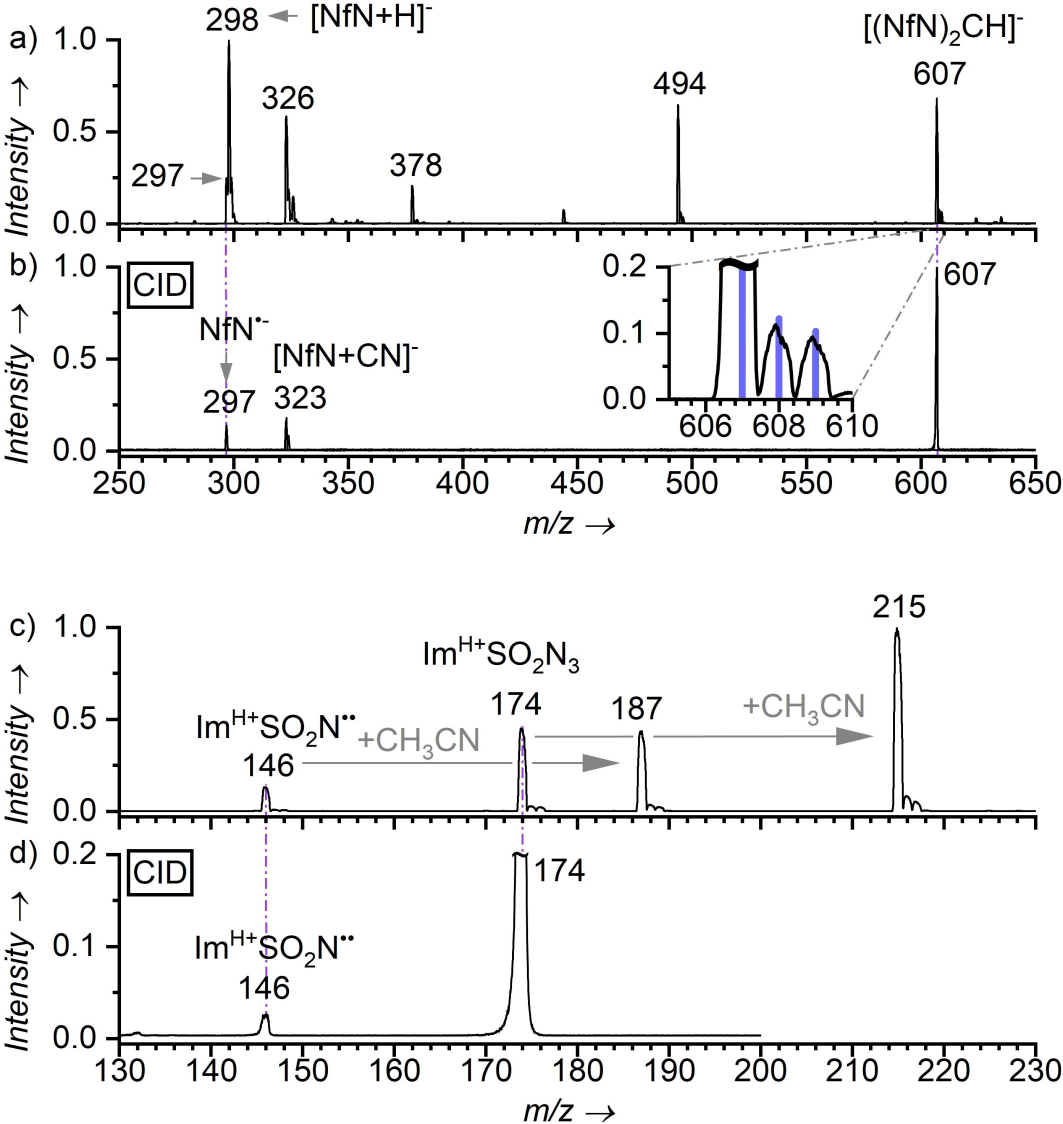
Generation of nitrene radical anion and nitrene intermediates. a) Electrospray ionization mass spectrum of a mixture of nonaflyl azide (NfN_3_, 2.6 mM) and Ru(bipy)_3_(PF_6_)_2_ (83 μM, 3 mol.%) in CD_3_CN after irradiation (445 nm LEDs, 5 min) in a vial. b) Collision‐induced dissociation (CID) spectrum of ions with *m/z* 607 (*p*_Xe_=0.2 mTorr, *E*_CM_=4.8 eV). Insert: Comparison of the experimental and theoretical isotopic patterns of (C_4_F_9_SO_2_N)_2_CH (blue lines). c) Electrospray ionization mass spectrum of imidazole‐1‐sulfonyl azide (Im^H+^SO_2_N_3_) hydrogensulfate solution in acetonitrile (168 μM). d) CID spectrum of ions with *m/z* 174 (*p*_Xe_=0.2 mTorr, *E*_CM_=10.5 eV). Signals are annotated by their *m/z* ratio and tentative structures.

Mass spectrometry offers ideal methods for studying uni‐ and bimolecular reactivity of highly reactive species under well‐defined conditions.^[12]^ In addition, the structure of these reactive species can be characterized with infrared photodissociation spectroscopy.^[13]^ Thus, the combination offers a direct structure‐reactivity correlation. Employing this strategy, we have first investigated NfN^.−^ generated from the reaction mixture of nonaflyl azide (NfN_3_) and Ru(bipy)_3_^2+^ after the irradiation at 445 nm. The NfN^.−^ anions (m/z 297) cannot be directly detected from solution by electrospray ionization mass spectrometry (ESI‐MS) presumably due to their high reactivity.[^14^, ^15^, ^16^] However, they can be generated in the gas phase by fragmentation of one of the photodegradation products ‐ namely (NfN)_2_CH^.−^ (m/z 607, Figure 2b, see the Supporting Information for details). Hence, using the in‐source collisional activation^[17]^ we generated the desired NfN^.−^ radical anions in a sufficient abundance for further experiments (Figures 2a and S20).

Neutral species cannot be directly studied by mass spectrometry; therefore, we employed a charge‐tagged azide analogue bearing an electron‐accepting imidazolium group instead of the nonaflyl chain (Im^H+^SO_2_N_3_, Figure 1). The ESI‐MS spectrum of the acetonitrile solution of Im^H+^SO_2_N_3_ in the absence of Ru(bipy)_3_(PF_6_)_2_ shows the signals of the parent ions (m/z 174) as well as of the nitrene fragment Im^H+^SO_2_N^..^ (m/z 146, see Figure 2c and d). In addition, we detected acetonitrile adducts of both ions at m/z 187 and 215, respectively.

Next, we tested the reactivity of the generated intermediates with various substrates in the gas phase (Table 1). Nonaflyl nitrene radical‐anion (NfN^.−^, alternative nomenclature could be nonaflyl imidyl radical anion^[18]^) readily abstracts hydrogen atoms from ethanethiol and acetylacetone (present mainly in the enol form in the gas phase, Figures S30–S33).^[19]^ However, NfN^.−^ is unreactive towards 1,4‐cyclohexadiene (S34), acetone, methanol, THF, cyclohexane and ethane. The bond dissociation energy of reactive ethanethiol (88.6±2^[20]^) is higher than the one of the unreactive 1,4‐cyclohexadiene (76.0±1.2^[21]^). The cleavage of the stronger S−H bond can be explained by a mechanism initiated by proton transfer, because the S−H bond is more prone to a heterolytic cleavage than the C−H bond. Therefore, NfN^.−^ reactivity pattern is consistent with proton‐coupled electron transfer (PCET) mechanism of the detected hydrogen atom transfer (HAT) reactions.^[22]^ The standalone proton transfer was excluded based on the absence of ethanethiolate and acetylacetonate peaks in the product mass spectra (Figures S30 and S32, for calculations and discussion refer to the Table S1 in the Supporting Information).

**Table 1:** Gas‐phase reactivity of nonaflyl nitrene radical‐anion (NfN^.−^), imidazole‐1‐sulfonyl nitrene (Im^H+^SO_2_N^..^) and imidazole‐1‐sulfonyl amidyl radical (Im^H+^SO_2_NH⋅).^[a]^

Collision gas	C−H *BDE* ^[23]^	Reactivity with		
	[kcal mol^−1^]	NfN^.−^	Im^H+^SO_2_N^..^	Im^H+^SO_2_NH⋅
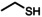	S−H: 89^[20]^	HAT	HAT^[b,c]^, 2xHAT, other^[e]^	HAT
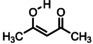	O−H_enol_: 90^[24]^	HAT	HAT^[b]^, 2xHAT	HAT
	C^3^−H_keto_: 88^[25]^			
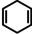	76^[21]^	Traces^[d]^	HAT^[b]^, 2xHAT, other^[e]^	HAT
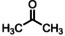	96^[26]^	None	HAT^[b]^, 2xHAT	HAT
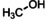	C−H: 96[^27^, ^28^]	None	HAT, 2xHAT	HAT
	O−H: 105[^27^, ^29^]			
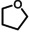	93^[30]^	None	HAT, 2xHAT	HAT
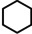	100^[31a]^	None	HAT, 2xHAT	HAT
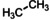	101[^27^, ^32^]	None	HAT^[f]^	Traces^[g]^

[a] 2xHAT=two consecutive hydrogen atom transfer steps. Conditions: *E*_CM_=0 eV, *p*_gas_=0.1–0.3 mTorr (see Figures S30–S68). [b] Intense subsequent fragmentation attesting a large exothermicity of HAT. [c] Additional pathway leading to a transfer of SH. [d] A trace signal of HAT that could be due to the reaction with 1,4‐CHD or with background impurities. [e] Product of nitrogen atom transfer – probably originating from fragmentation of the adduct. [f] *KIE* for CH_3_CD_3_ is ∼2.1–2.5, a faint signal of 2xHAT. [g] A trace signal of HAT that could be due to the reaction with ethane or with background impurities.

In contrast, Im^H+^SO_2_N^..^ was able to abstract a hydrogen atom from ethanethiol, acetylacetone, acetone, methanol, tetrahydrofuran, cyclohexane and even ethane, which clearly attests to a much larger reactivity of the free nitrene compared to the nitrene radical anion (Figure 3, Figures S37–S56).

**Figure 3:**
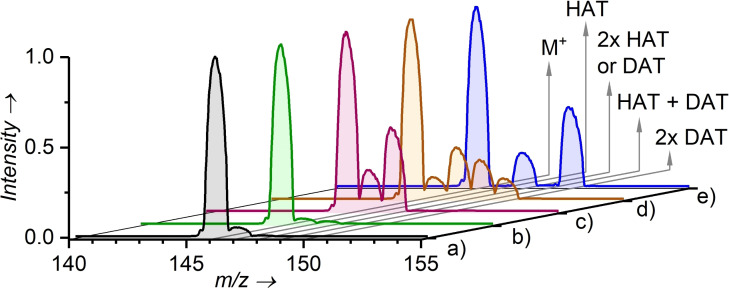
Reaction of Im^H+^SO_2_N^..^ (*E*_CM_=0 eV) with: a) ethane ‐ C_2_H_6_ (*p*_CH3CH3_=0.13 mTorr), b) ethane‐*d3* ‐ CH_3_CD_3_ (*p*_CH3CD3_=0.13 mTorr), c) cyclohexane C_6_H_12_ (*p*_C6H12_=0.12 mTorr), d) cyclohexane‐*d0/d12*‐C_6_H_12_+C_6_D_12_ (*p*_C6H12/C6D12_=0.14 mTorr), e) cyclohexane‐*d12*‐C_6_D_12_ (*p*_C6D12_=0.11 mTorr).

Interestingly, in the reactions of Im^H+^SO_2_N^..^ with cyclohexane and tetrahydrofuran we observed the hydrogen‐atom transfer (HAT) and also an intense double HAT (Figure 3‐trace c and e, Figures S50 and S51). This double HAT reactivity might correspond either to the dehydrogenation of a substrate molecule forming Im^H+^SO_2_NH_2_ or to two consecutive single HAT reactions where initially formed sulfonylamidyl radical Im^H+^SO_2_NH⋅ reacts with another molecule of the substrate. To resolve this ambiguity, we reacted Im^H+^SO_2_N^..^ with a 1 : 1 mixture of cyclohexane and cyclohexane‐*d_12_* (trace d in Figure 3).

The products of the double HAT show more or less statistical distribution of H and D (influenced by the kinetic isotope effect), which clearly indicates that the reaction mechanism does not correspond to the H_2_ or D_2_ eliminations from the cyclohexane, but rather to two independent HAT reactions. This result clearly illustrates that sulfonylamidyl radical Im^H+^SO_2_NH⋅ is still very reactive. We have further verified this by studying the reactivity of in‐source generated Im^H+^SO_2_NH⋅ (sprayed Im^H+^SO_2_N_3_ with tetrahydrofuran vapors in the electrospray ionization source, Figure S29). As expected, the reactivity of Im^H+^SO_2_NH⋅ is dominated by hydrogen atom transfer reactions.

The difference in reactivities of Im^H+^SO_2_N^..^ and Im^H+^SO_2_NH⋅ was revealed in their reaction with ethane (trace a in Figure 3). For Im^H+^SO_2_N^..^ we can observe almost exclusively single HAT, suggesting that the Im^H+^SO_2_NH⋅ product does not further react with ethane. In agreement, independently generated Im^H+^SO_2_NH⋅ reacted with ethane rather sluggishly (see Figure S67–pressure dependence). Hence, we used the fact that we see almost exclusively single HAT and determined the kinetic isotope effect (KIE) in the reaction of Im^H+^SO_2_N^..^ with CH_3_CD_3_ (trace b in Figure 3, pressure dependence in Figure S55). The spectrum shows that the KIE is in the range of 2.1–2.5. This value is typical for HAT reactions of free radicals in the gas phase.^[33]^ In summary, Im^H+^SO_2_N^..^ is slightly more reactive than Im^H+^SO_2_NH⋅ and substantially more reactive than NfN^.−^.

In solution, the initial HAT between the nitrene reactant R'N^..^ and a hydrocarbon RH could be followed by a rebound of the radicals to yield R'NH(R).[^1^, ^2^, ^3^] We cannot observe this process directly in the gas phase, but we probably observed fragments of the rebound products Im^H+^SO_2_NH(R). This is because the rebound of two radicals is very exothermic. The excess energy cannot be dissipated in the dilute gas phase; therefore, the rebound products usually release their energy by eliminating a small fragment. Here, we observe the formation of protonated imidazole (Im^H+^) and nitrogen insertion (RNH^+^) which might be signs of the fragmentation of the rebound product Im^H+^SO_2_NH(R) (see also Figures S40, S41 and S56).

To connect the observed reactivities with the structures of the intermediates, we have characterized the studied ions with IR photodissociation spectroscopy.^[13]^ Several sulfonylnitrenes (RSO_2_N^..^, R=F,^[5a]^ Ph,^[5b]^ N_3_,^[5c]^ CH_2_Cl, CHCl_2_,^[5d]^ C_4_H_3_S,^[5]^ CF_3_,^[7a]^ *p*‐BrPh, *p*‐MePh, CH_3_ ^[7b]^) were characterized by IR spectra using matrix isolation[^5^, ^7a^] or UV‐IR pump‐probe techniques.^[7b]^ However, no structural characterization of sulfonylamide radicals exists so far to the best of our knowledge. We measured infrared photodissociation spectra of mass‐selected Im^H+^SO_2_N^..^ and of Im^H+^SO_2_NH⋅.^[35]^

**Figure 4:**
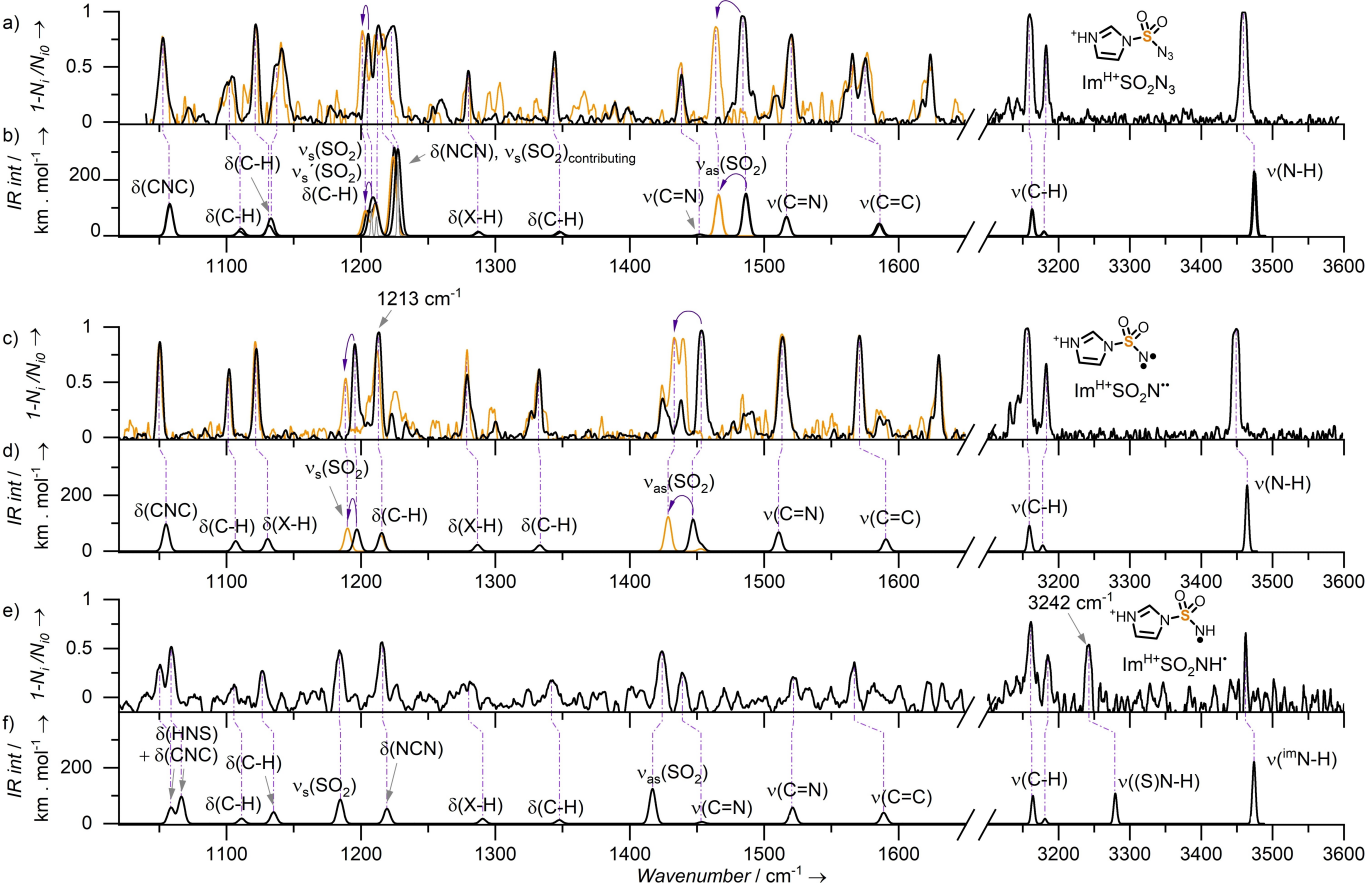
Comparison of helium tagging IRPD and calculated (B3LYP/6‐311+G**, GD3‐BJ, PC‐3 basis set used for the sulfur atom^[34]^) vibrational spectra. a) IRPD and b) calculated spectra of Im^H+^SO_2_N_3_. c) IRPD and d) calculated spectra of Im^H+^SO_2_N^..^. e) IRPD and f) calculated spectra of Im^H+^SO_2_NH⋅. The orange lines represent spectra of ^34^S isomer. Grey line in b) represents calculated spectrum with a narrower peak width in the 1200–1250 cm^−1^ region. Two black lines in the calculated spectrum b) represent two conformers which differ by 1 kJ mol^−1^ in energy and are separated by 5 kJ mol^−1^ barrier.

First, we tested the experimental and theoretical methods by studying the stable precursor Im^H+^SO_2_N_3_. The predicted structure of Im^H+^SO_2_N_3_ has a synperiplanar geometry between the azide function and one of the S=O bonds which agrees with geometries of other sulfonyl azides (see Figure S74).[^36^, ^37^] The corresponding theoretical IR spectrum matches very well with the experimental infrared photodissociation spectrum (IRPD) of mass‐selected Im^H+^SO_2_N_3_ (Figure 4a and b). We confirmed the assignment by comparing the IR spectra of the ^32^S and ^34^S isotopologs (black and yellow spectra in Figure 4a and b).^[13]^ The band at 1484.5 cm^−1^ (shifting to 1464 cm^−1^ upon the ^34^S labelling) corresponds to the SO_2_ asymmetric vibration. The SO_2_ symmetric vibration band is in the range 1205–1230 cm^−1^. This region shows several bands of vibrations coupled with the S=O vibration. Most likely, the symmetric SO_2_ vibration band is at 1205.5 cm^−1^ and red‐shifts to 1201 cm^−1^ upon the isotopic labelling (4.5 cm^−1^ experimentally, 5 cm^−1^ theoretically). The experimental band at 1624 cm^−1^ might correspond to a combination band of the imidazole C−H out‐of‐plane wagging and twisting vibrations which is present in the anharmonic calculations (Figure S70). Overall, the agreement between the experiment and theory demonstrates that the chosen theoretical method provides IR spectra that can serve for the assigning of the correct structure of this type of ions.

With the experimental spectrum of Im^H+^SO_2_N_3_ as a reference and the benchmark in our hand, we measured the spectrum of Im^H+^SO_2_N^..^. The experimental spectrum of Im^H+^SO_2_N^..^ (Figure 4c) matches perfectly with the calculated one (Figure 4d). The comparison of the spectra of ions with ^32^S and ^34^S isotopes visualizes the S=O vibrations. The isotopic shift of the asymmetric SO_2_ vibration band (1453 cm^−1^) is −20 cm^−1^ (theoretically: −18.5 cm^−1^) and of the SO_2_ symmetric vibration band (1195.5 cm^−1^) −7 cm^−1^ (theoretically: −7 cm^−1^). We excluded all other possible isomers including pseudo‐Curtius rearranged Im^H+^NSO_2_ based on the agreement between the calculation and the experiment (Figure S71).

We also measured the IRPD spectrum of the amidyl radical Im^H+^SO_2_NH⋅ (Figure 4e). The frequency of the N−H stretch of the amidyl group is 3242 cm^−1^, which is unusually low for a gaseous N−H stretch.^[38]^ The asymmetric (1424 cm^−1^) and symmetric (1184 cm^−1^) SO_2_ vibrations are slightly red‐shifted with respect to those of Im^H+^SO_2_N^..^.

**Figure 5:**
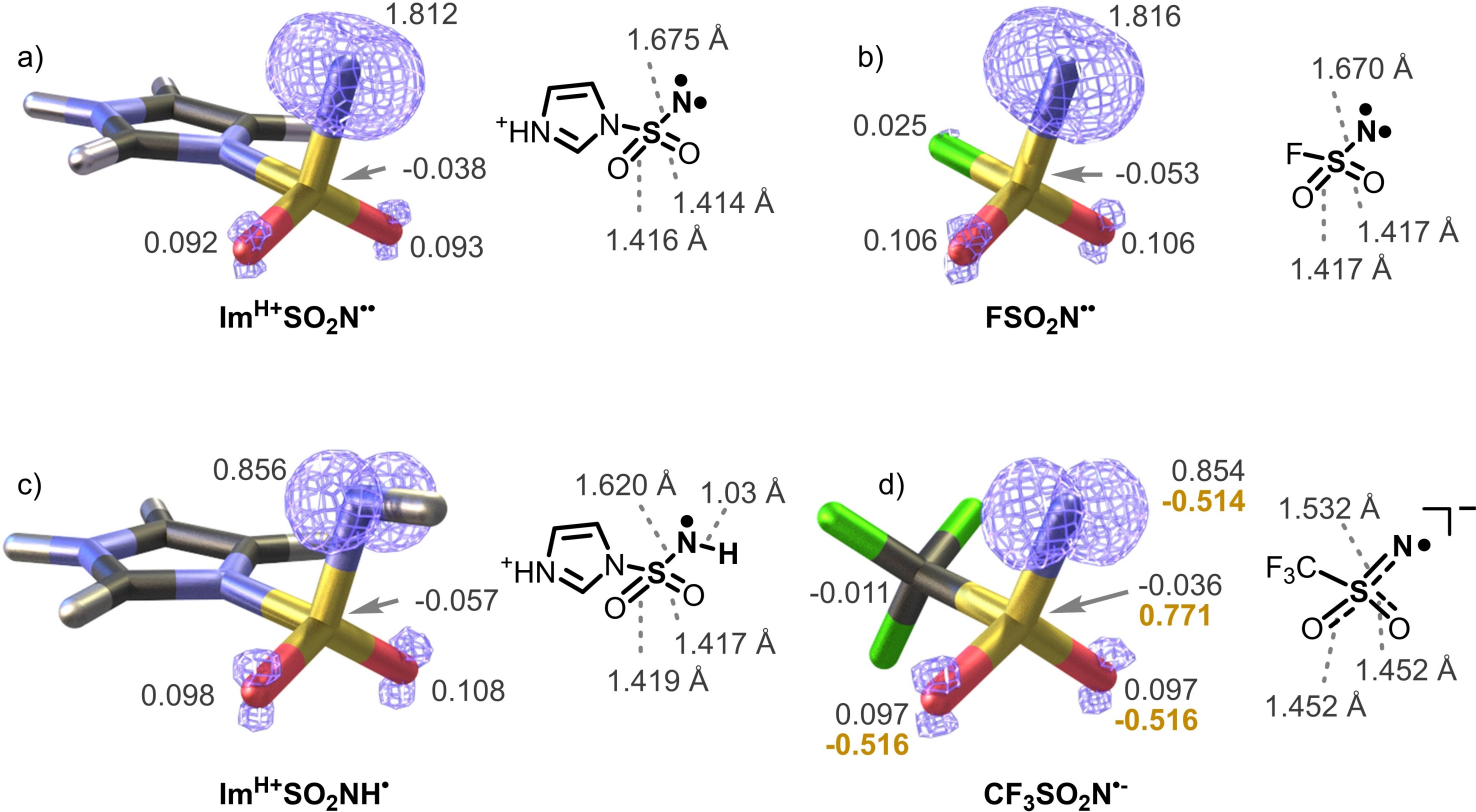
Optimized structures with spin density (blue, the isosurface value=0.02 e, the numbers refer to the Mulliken spin densities) and bond lengths of a) Im^H+^SO_2_N^..^, b) FSO_2_N^..^, c) Im^H+^SO_2_NH⋅ and d) CF_3_SO_2_N^.−^ (Mulliken charges denoted in gold).

The agreement between the experimental IR spectra and the theoretical prediction shows that the DFT method describes these radicals well. Hence, we analyzed the calculated spin densities and the geometries in detail (Figure 5). The spin density is almost exclusively localized at the nitrogen atom in both Im^H+^SO_2_N^..^ and Im^H+^SO_2_NH⋅ with only a small delocalization towards the oxygen atoms. This delocalization is larger for Im^H+^SO_2_NH⋅ which is associated with prolongation of the S−O bonds and thus the red‐shift observed in the IRPD spectrum above.

For comparison, we also calculated the previously reported FSO_2_N^..^. The spin density of FSO_2_N^..^ is slightly more delocalized towards the oxygen atoms than in Im^H+^SO_2_N^..^, which agrees with the red‐shifted IR characteristics of the S=O bands (the SO_2_ asymmetric vibration of FSO_2_N^..^ is red‐shifted by 26.5 cm^−1^ with respect to that of Im^H+^SO_2_N^..^). Finally, we also assessed the radical anion CF_3_SO_2_N^.−^ representing the other possible reactive species generated in the photochemical reaction (see Figure 1). This species could be compared to Im^H+^SO_2_NH⋅ as its protonated analogue. The localization of the spin density at the nitrogen atom is almost identical for both species, but the bond lengths differ significantly. The shorter S−N bond and the substantially longer S−O bonds of the radical anion correlate with the delocalization of the negative charge at the oxygen atoms. The S−N bond has thus partially a double‐bond character. This and the delocalized negative charge are probably responsible for the lower reactivity that we observed for this ion and for the mechanistic shift of the HAT reaction pathway towards proton‐coupled electron‐transfer reaction mechanism.

## Conclusion

We directly compared the reactivity of three reactive species: nonaflyl nitrene radical‐anion (NfN^.−^), imidazole‐1‐sulfonyl amidyl radical (Im^H+^SO_2_NH⋅) and triplet imidazole‐1‐sulfonyl nitrene (Im^H+^SO_2_N^..^). These radicals are models of possible intermediates in photocatalyzed reactions of sulfonyl azides that lead to the activation of aliphatic C−H bonds. The reactivities observed in solution are in clear correlation with the reactivity of the neutral nitrene and the amidyl radical. Both species react as typical radicals in hydrogen‐atom transfer reactions. The nitrene activates even highly unreactive species like ethane with the kinetic isotope effect of ∼2.3. On the other hand, the nitrene radical‐anion reacted only with “acidic” S−H or O−H bonds suggesting a proton‐ coupled electron‐transfer pathway. However, protonation of the nitrene radical anions leads to the amidyl radicals boosting the reactivity and enabling the C−H activation reactions (albeit the reactivity is lower than that of the nitrenes). Hence, tuning the properties of the photocatalyst can steer the reactivity along different paths (Figure 1) providing access to more selective reactions.

## Experimental Section

Nonaflyl azide (NfN_3_) and 3‐azidosulfonyl‐3H‐imidazol‐1‐ium Hydrogen Sulfate (Im^H+^SO_2_N_3_*HSO_4_^−^) were prepared by a slightly modified published procedures (See Supporting Information).^[39]^ Mass spectra were measured by Finnigan TSQ‐7000 quadrupole‐octopole‐quadrupole mass spectrometer equipped with electrospray ionization source (ESI‐MS). The same instrument was used for the gas‐phase reactivity experiments. In the reactivity experiments, the desired ions were mass selected by the first quadrupole and collided with a desired gas in the octopole collision cell at nominal zero collision energy. The collision energy was determined by retarding potential analysis (Supporting Information). The collision products were mass‐analysed by the third quadrupole and detected by a Daly‐type detector.

Gas‐phase infrared photodissociation spectra were recorded with a custom‐built instrument described in a detail elsewhere.^[13h]^ The instrument is based on TSQ‐7000, therefore the ions could be generated and mass‐selected in the same way as for the reactivity studies. The mass‐selected ions were guided through a quadrupole bender and an octopole to a linear wire quadrupole trap operating at 3 K. The ions were trapped and thermalized by the pulsed helium buffer gas. The thermalized ions formed complexes with helium. The trapped ions were irradiated in odd cycles. Finally, the ions were extracted from the trap, mass‐analyzed by a quadrupole and detected by a Daly‐type detector. The photon absorption was monitored as the depletion of the number of helium complexes detected with (*N_i_* (ν)) vs. without (*N_i0_*) IR irradiation of the trapped ions (1‐*N_i_* (ν)/*N_i0_*). The IR photons were generated by tunable OPO/OPA photon source.

The calculations were performed with Gaussian G16 using the B3LYP−D3 functional[^40^, ^41^] and the PC‐3 basis set for sulphur[^34d^, ^34e^] and the 6–311+G** basis set for remaining atoms. The harmonic IR spectra were scaled by factor 0.99 in the range below 2500 cm^−1^ and by factor 0.965 in the range above 2500 cm^−1^. Scaling factors have been determined based on overlap between experimental and theoretical spectrum of Im^H+^SO_2_N_3_.

## Conflict of interest

The authors declare no conflict of interest.

1

## Supporting information

As a service to our authors and readers, this journal provides supporting information supplied by the authors. Such materials are peer reviewed and may be re‐organized for online delivery, but are not copy‐edited or typeset. Technical support issues arising from supporting information (other than missing files) should be addressed to the authors.

Supporting Information

## Data Availability

The data that support the findings of this study are available in the supplementary material of this article.
