# Supplementary material for: Sulfonyl Nitrene and Amidyl Radical: Structure and Reactivity
Source: Chemistry. 2022 Apr 5;28(28):e202104493. doi: 10.1002/chem.202104493 (PMC9323475; doi:10.1002/chem.202104493)
Supplement: Supplementary file 1 — Supporting Information [file CHEM-28-0-s001.pdf]

Chemistry–A European Journal

Supporting Information

Sulfonyl Nitrene and Amidyl Radical: Structure and Reactivity

Jan Zelenka, Aleksandr Pereverzev, Ullrich Jahn, and Jana Roithová*

Table of content

1 Synthesis ... 2

1.1 Preparation of nonaflyl azide (NfN_3) ... 2

1.2 Preparation of 3-azidosulfonyl-3H-imidazol-1-ium Hydrogen Sulfate ($Im^{H+}SO_2N_3*HSO_4^-$) ... 2

2 ESI-MS and ESI-MS^2 experiments ... 3

2.1 Experimental details (S2-S4) ... 3

2.1.1 Instrumentation ... 3

2.1.2 Irradiation layout ... 3

2.1.3 Ionization method: overpressure unit ... 4

2.2 MS and CID data and conditions (S5-S26) ... 4

2.2.1 Source spectra acquired without irradiation – Nonaflyl azide ... 5

2.2.2 Source spectra acquired with irradiation – Nonaflyl azide ... 6

2.2.3 Fragmentations – Nonaflyl azide derivates ... 10

2.2.4 Fragmentation of $Im^{H+}SO_2N^{••}$ ... 11

2.2.5 Source spectra acquired without irradiation – Imidazoliumsulfonyl azide ... 12

2.2.6 Source spectra acquired with irradiation – imidazole-1-sulfonyl azide ... 12

2.3 Gas-phase reactivity of selected ions (S27-S68) ... 13

2.3.1 Conditions used for generation of reactive ions and their MS spectra ... 13

2.3.2 Gas-phase reactivity of $NfN^{•-}$ ... 15

2.3.3 Gas-phase reactivity of $Im^{H+}SO_2N^{••}$ ... 18

2.3.4 Gas-phase reactivity of $Im^{H+}SO_2NH^{•}$ ... 25

3 IRPD spectra, DFT calculations (S69-S76) ... 29

3.1 Experimental Details ... 29

3.2 Computational Details ... 30

3.3 Benchmark of computational methods used to predict vibrational spectra ... 30

3.4 Geometry of optimized DFT structure of $ImH^+SO_2N_3$ ... 33

3.5 Enthalpy of selected $NfN^{•-}$ reactions ... 33

3.6 Enthalphy of possible $NfN^{•-}$ fragmentation channels ... 33

4 Cyclic voltammetry ... 34

5 XYZ coordinates of the calculated structures ... 35

6 NMR spectra of the synthetized compounds ... 37

1 Synthesis

Nonafluorobutanesulfonyl azide (nonaflyl azide, **NfN**$_3$) and imidazole-1-sulfonyl azide hydrogen sulfate (**Im**$^{H+}$**SO**$_2$**N**$_3$*HSO_4^-) were synthetized in accordance with published procedures (Figure S1).[1] Special caution was taken during the distillation of nonaflyl azide to avoid accidental deflagration caused by overheating of the bath above 100 °C.[2] For imidazolium-substituted sulfonyl azide (**Im**$^{H+}$**SO**$_2$**N**$_3$*HSO_4^-), the amount of sulfuric acid added was corrected based on molar ratios. NMR spectrum for referencing of imidazole-1-sulfonyl azide hydrogen sulfate was taken from the literature.[3] The NMR values reported in the synthetic paper[1c] belongs to decomposition product, as apparent from our NMR experiments and from spectra reported by Fischer.[3] The amount of sulfuric acid reported in ref. 1c is obviously incorrect (method A: 156 mmol of concentrated H_2SO_4 is definitely not 3 mL, but roughly 8.5 mL).

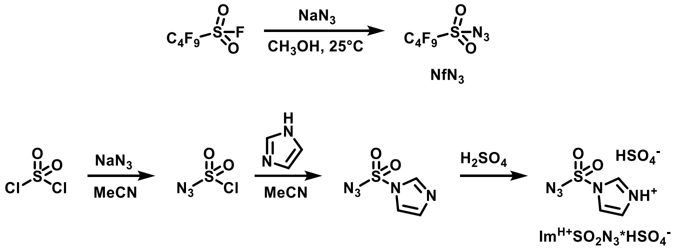

Figure S1. Synthesis of **NfN**$_3$ and **Im**$^{H+}$**SO**$_2$**N**$_3$*HSO_4^-.

1.1 Preparation of nonaflyl azide (NfN$_3$)

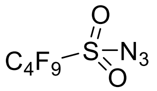

To a suspension of NaN_3 (8.01 g, 122 mmol, 99%, Acros) in methanol (220 mL) nonafluorobutanesulfonyl fluoride (20 mL, 106 mmol, 95%, Fluorochem) was slowly added. At the beginning of the addition the solution turned orange for a few moments, then it quickly became colorless again. The temperature slightly raised itself a few minutes after the addition and the suspension started to turn into emulsion. After stirring for 24 h, the reaction mixture was poured onto ice (400 mL). Persistent emulsion formed. Addition of 200 mL of H_2O and 100 mL of brine did not help to separate the layers. The emulsion was filtered through a frit, which improved the separation. Separation was achieved after addition of Na_2SO_4 and subsequent filtration. After separation, target compound was dried over Na_2SO_4. ^{1}H NMR spectrum of neat sample of crude product (23.4 g) showed intense signals – probably of residual methanol. For this reason, the compound was distilled prior to use. Yield 19.68 g (57%) after distillation.

^{19}F NMR (471 MHz, neat, DMSO-*d*$_6$ external standard) δ -83.07 (tt, *J* = 10.2, 2.4 Hz, 3F, ***CF***$_3$), -111.33 (t, *J* = 14.2 Hz, 2F, ***CF***$_2$*S*), -122.52 (m, 2F, *CF*$_3$***CF***$_2$), -127.74 (m, 2F, ***CF***$_2$*CF*$_2$*S*).

^{13}C NMR (126 MHz, neat, DMSO-*d*$_6$ external standard) δ 116.62(***CF***$_3$), 114.80 (***CF***$_2$*S*), 109.62 (*CF*$_3$***CF***$_2$), 108.01 (***CF***$_2$ *CF*$_2$*S*).

1.2 Preparation of 3-azidosulfonyl-3H-imidazol-1-ium Hydrogen Sulfate (Im^{H+}SO$_2$N$_3$*HSO$_4^-$)

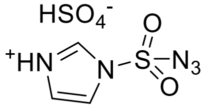

A suspension of NaN_3 (10.2 g, 157 mmol, Fisher scientific) in acetonitrile (77 mL, dry) was cooled in a water-ice bath (0 °C) under argon atmosphere. At this temperature sulfuryl chloride (13 mL, 158 mmol, 98,5%, acros) was slowly added over 10 min. This mixture was warmed to room temperature. The suspension became thicker after 1 h. After stirring for 18 h, the suspension was cooled-down with water-ice bath back to 0 °C and imidazole (20.15 g, 300.4 mmol) was added over a course of 10 min. The temperature was kept at 0 °C for another 4.5 h. The mixture was then warmed to room temperature, diluted with EtOAc (250 mL) and extracted with 350 mL of saturated $NaHCO_3$ solution and then with 250 mL of H_2O. The organic layer was dried over $MgSO_4$. After removal of the drying agent, the solution was once again cooled to 0 °C and H_2SO_4 (8.8 mL, 162 mmol, 98.5%) was added. After 13 hours of stirring the formed precipitate was filtered off and flushed with 50 mL of EtOAc. After drying in air on a frit and subsequently in vacuum, a colorless crystalline compound 27.89g (65%) was obtained.

NOTE: NMR sample needs to be measured within minutes, otherwise decomposition occurs (see recorded NMR spectra).

^{1}H NMR (400 MHz, DMSO-*d*$_6$, fresh sample) δ 8.56 (m, 1H), 7.96 (m, 1H), 7.32 (m, 1H).

elemental analysis calcd (%) for $C_{20}H_{32}N_2O_5$: C 13.29, N 25.82; found: C 13.71, N 25.72.

2 ESI-MS and ESI-MS^2 experiments

2.1 Experimental details (S2-S4)

2.1.1 Instrumentation

The reported ESI-MS and ESI-MS/MS spectra were measured with Finnigan TSQ-7000 instrument with an electrospray ionization source. We used 1 W laser diode (445 nm, blue, e-bay) which was operated on 0.5-1A current for capillary tip irradiation. For irradiation in vial we used a set of 4 LED diodes (4 x 700mA, 445nm, Luxeon Rebel). In all cases, we used fused silica capillary (internal diameter 100 µm, outer diameter 190 µm, polyimide coating, PostNova part no. Z-FSS-100190) for the ESI.

2.1.2 Irradiation layout

We irradiated a sample at two different positions in our ESI-MS experiments: in a vial, or at the tip of a capillary. Vial irradiation was carried out with 4 diodes as illustrated in Figure S2. For the tip irradiation we used a laser diode beam which was focused on the tip of the capillary. We used an optical table shown in Figure S3 to focus the beam. Both methods were fully described elsewhere with schematics and photo-documentation.[4]

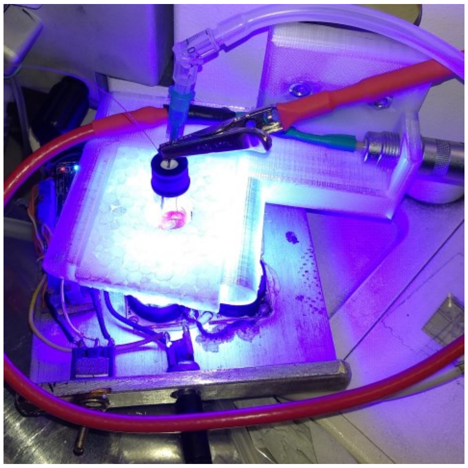

Figure S2. Vial irradiation in course of ESI-MS spraying.

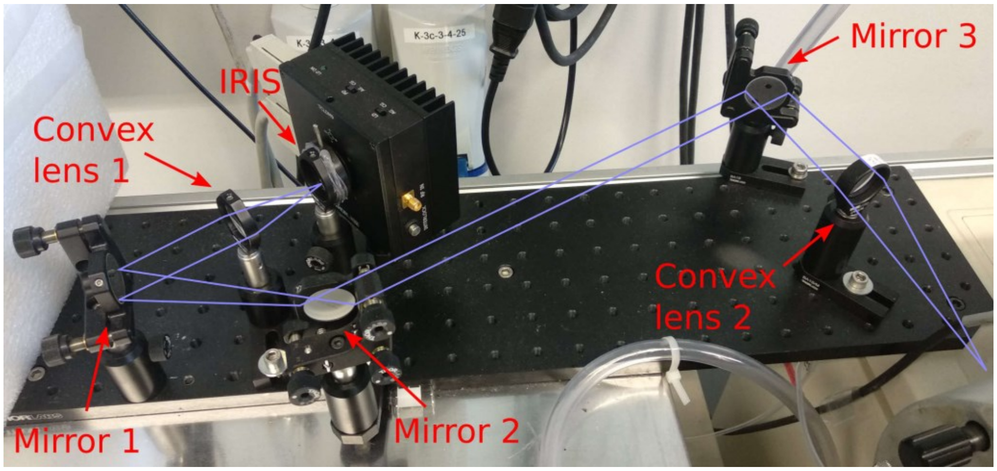

Figure S3. Optical table used for the capillary tip irradiation. The IRIS is positioned in a focal point of the LED laser source and filters the light beam. This beam is then collimated by the convex lens 1 and refocused onto the tip of the capillary by the convex lens 2.

2.1.3 Ionization method: overpressure unit

We pressurized a vial with a sample to deliver the analyzed solution into the mass spectrometer. This method was described elsewhere in detail.[4a,5] One of our implementations is documented in the Figure S4.

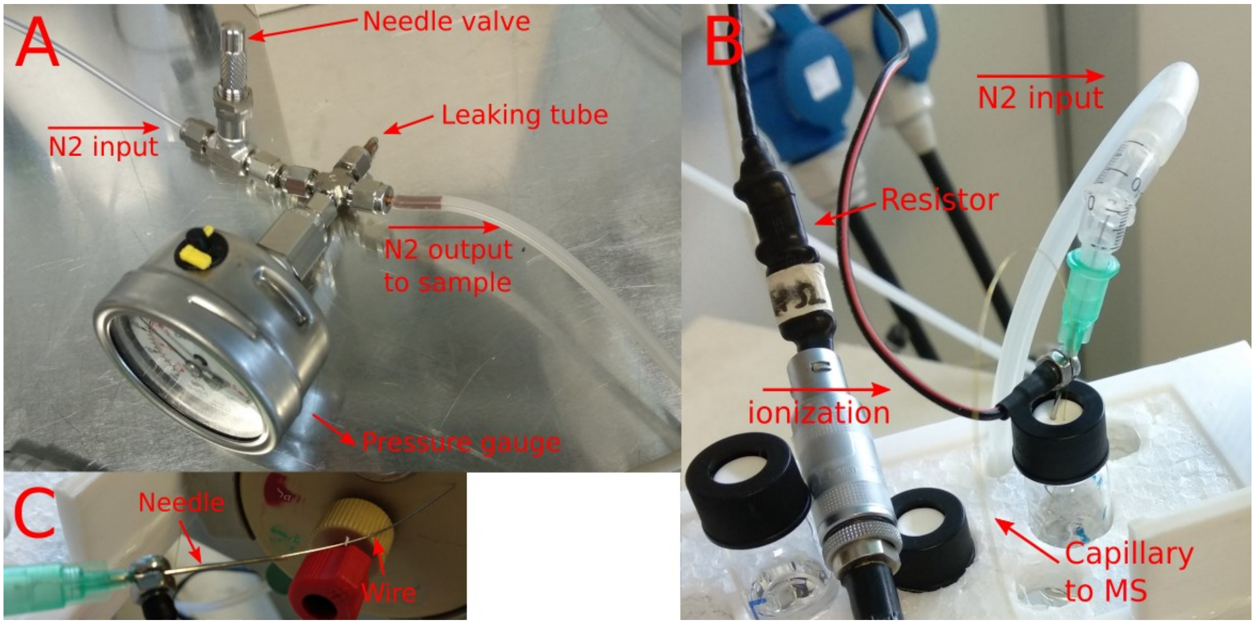

Figure S4. A) Pressure regulator by which the desired vial pressure is set. Tube with a small hole (leaking tube) helps substantially to stabilize the pressure. B) Syringe with a wire in it by which ionization voltage and pressure were introduced into the vial with sample through a cap with a septum. The sample is delivered to the electrospray source through fused silica capillary which is submerged into the vial. C) Detail of the needle with stainless steel wire in it used for ionization. The wire is submerged into the sample whereas the needle tip end should be above the sample.

2.2 MS and CID data and conditions (S5-S26)

In a case that a mixture of nonaflyl azide and $Ru(bipy)_3(PF_6)_2$ is irradiated at the tip of the capillary, a weak signal of nonaflyl nitrene radical anion appears (**NfN$^{\bullet -}$**, m/z 297, Figures S14 and S16). This signal is absent when the vial is irradiated instead of the tip under the same ionization conditions (Figure S15). It is also absent in the case that a non-deuterated acetonitrile is used as a solvent (Figure S12). The peak of **NfN$^{\bullet -}$** can also be generated artificially by fragmentation of m/z 607 upon hardening of the electrospray ionization source conditions (in-source fragmentation) regardless of the irradiation technique employed (compare Figure S15 – soft ESI conditions with Figure S17 – hardened ESI conditions).

Adduct corresponding to the m/z 607 ($(NfN)_2CH^{\bullet -}$) probably stems from photocatalyzed decomposition of $Ru(bipy)_3(PF_6)_2$ by **NfN**-based radicals as bipyridine is the only source of the C-H fragment present in the deuterated acetonitrile. Alternatively, it may stem from acetonitrile with subsequent D/H exchange, or from some trace impurity present in the sample. As apparent from the Figure S13 it is one of a many **NfN$^{\bullet -}$** adducts formed during photodegradation. The chloride in $C_4F_9NCl^-$ adduct visible in the Figure S13 probably originates from Cl^- contamination of $Ru(bipy)_3(PF_6)_2$ (see the spectrum S5). The ion of the m/z 607 did not exchange any hydrogen atom upon addition of D2O to the mixture. Ion of m/z 607 was also formed when a concentrated mixture of NfN3 and $Ru(bipy)_3(PF_6)_2$ in non-deuterated acetonitrile was irradiated (sample and conditions were similar to the one described in the Figure S27).

2.2.1 Source spectra acquired without irradiation – Nonaflyl azide

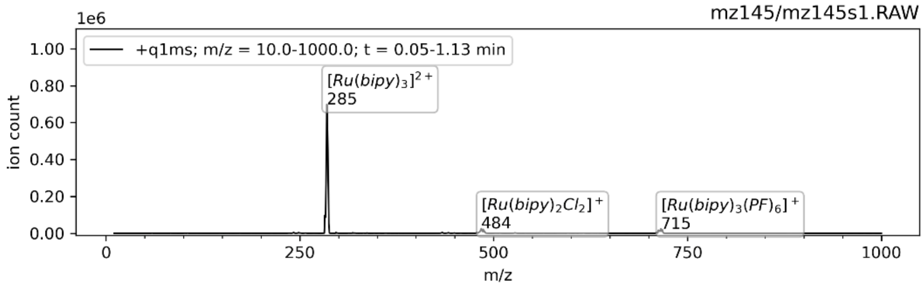

Figure S5. Positive ESI-MS spectrum of $Ru(bipy)_3(PF_6)_2$ in CH_3CN. Conditions: electron multiplier voltage 1500 V, sheath gas pressure 20 psi, auxiliary gas not used, spray voltage 4.5 kV, capillary temperature 243 °C, capillary voltage 0 V, tube lens voltage 70 V (rather soft ESI source conditions). Sample preparation: 0.12 mg of $Ru(bipy)_3(PF_6)_2$ was dissolved in 1 ml of acetonitrile. 100 µl of the concentrated solution was then diluted with 2 ml of acetonitrile.

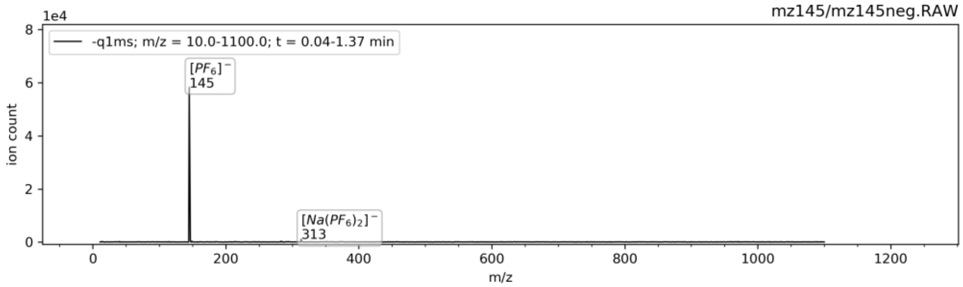

Figure S6. Negative ESI-MS spectrum of $Ru(bipy)_3(PF_6)_2$ in CH_3CN. Conditions: sheath gas pressure 20 psi, auxiliary gas not used, spray voltage 4.5 kV, capillary temperature 250 °C, capillary voltage 0 V, tube lens voltage -60 V (rather soft ESI source conditions). Sample preparation is the same as in the Figure S5.

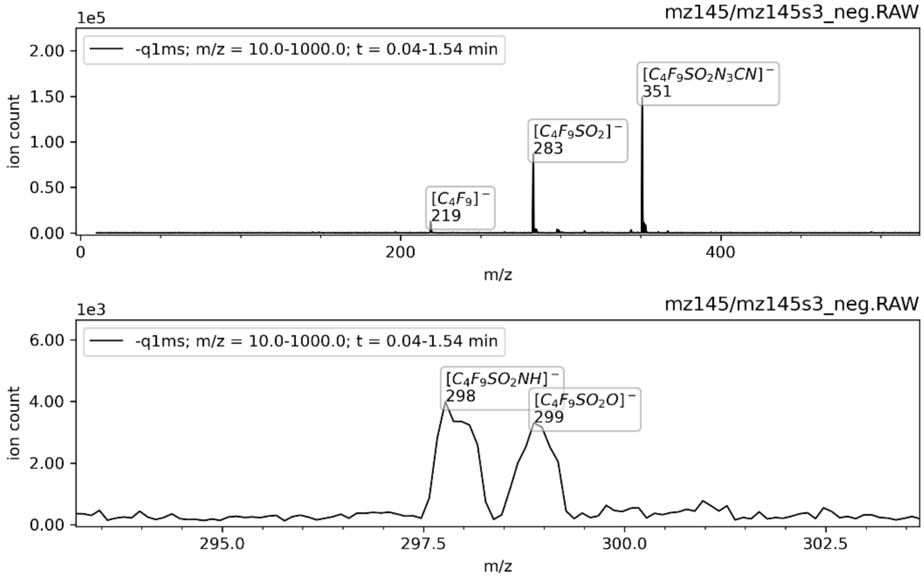

Figure S7. Negative ESI-MS spectrum of NfN_3 in CH_3CN. Conditions: sheath gas pressure 20 psi, auxiliary gas not used, spray voltage 4.5 kV, capillary temperature 250 °C, capillary voltage 0 V, tube lens voltage -60 V (rather soft ESI source conditions). Sample preparation: 8.09 mg of NfN_3 was dissolved in 4 ml of acetonitrile. 100 µl of the concentrated solution was then diluted with 2 ml of acetonitrile. Small peaks of nonaflyl amidyl anion and nonaflate anion are visible upon zooming.

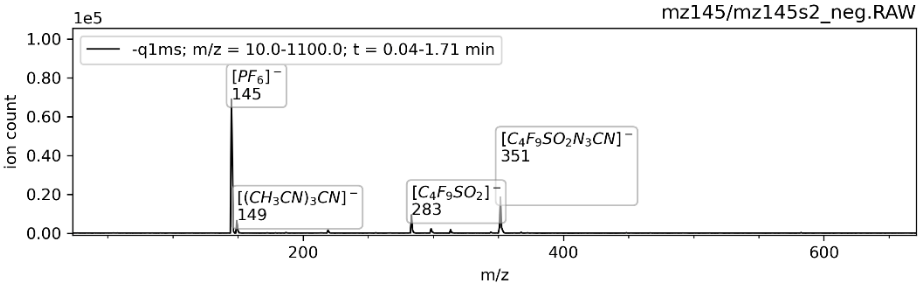

Figure S8. Negative ESI-MS spectrum of NfN_3 with $Ru(bipy)_3(PF_6)_2$ in CH_3CN. Conditions: sheath gas pressure 20 psi, auxiliary gas not used, spray voltage 4.5 kV, capillary temperature 250 °C, capillary voltage 0 V, tube lens voltage -60 V (rather soft ESI source conditions). Sample preparation: 8.09 mg of NfN_3 was dissolved in 4 ml of acetonitrile. 100 µl of this concentrated solution was subsequently added to $Ru(bipy)_3$ solution used in the Figure S5.

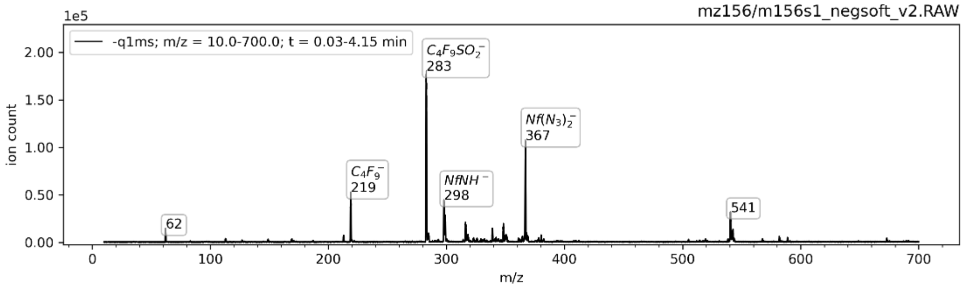

Figure S9. Negative ESI-MS spectrum of NfN_3 in CD_3CN. Conditions: electron multiplier voltage 1800 V, sheath gas pressure 12 psi, auxiliary gas not used, spray voltage 3.5 kV, capillary temperature 248 °C, capillary voltage 0 V, tube lens voltage -60 V (rather soft ESI source conditions). Sample preparation: 2.95 mg of NfN_3 was dissolved in 0.5 ml of acetonitrile-*d*$_3$. 100 µl of the concentrated solution was then diluted with 0.5 ml of acetonitrile-*d*$_3$.

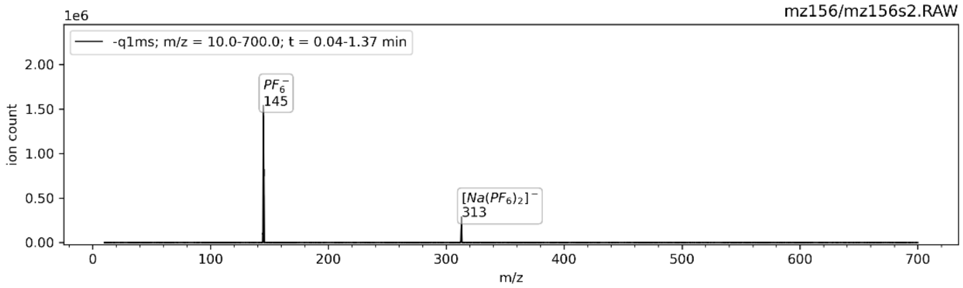

Figure S10. Negative ESI-MS spectrum of $Ru(bipy)_3(PF_6)_2$ in CD_3CN. Conditions: electron multiplier voltage 1500 V, sheath gas pressure 12 psi, auxiliary gas not used, spray voltage 3.5 kV, capillary temperature 240 °C, capillary voltage 0 V, tube lens voltage -60 V (rather soft ESI source conditions). Sample preparation: 0.23 mg of $Ru(bipy)_3$ was dissolved in 0.5 ml of acetonitrile-*d*$_3$. 100 µl of the concentrated solution was then diluted with 0.5 ml of acetonitrile-*d*$_3$.

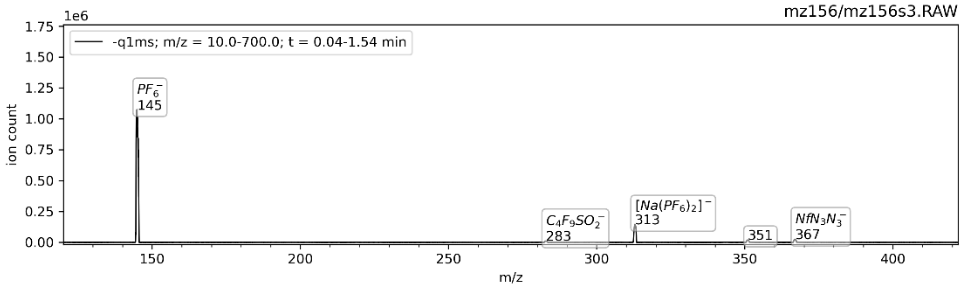

Figure S11. Negative ESI-MS spectrum of $Ru(bipy)_3(PF_6)_2$ and NfN_3 in CD_3CN. Conditions: electron multiplier voltage 1500 V, sheath gas pressure 14 psi, auxiliary gas not used, spray voltage 5.5 kV, capillary temperature 250 °C, capillary voltage 0 V, tube lens voltage -60 V (rather soft ESI source conditions). Sample preparation: 2.95 mg of NfN_3 was dissolved in 0.5 ml of acetonitrile-*d*$_3$. 100 µl of the concentrated solution was then added to the sample used in Figure S10.

2.2.2 Source spectra acquired with irradiation – Nonaflyl azide

When nonaflyl azide solution was irradiated, no changes in the ESI-MS spectrum were observed. Upon $Ru(bipy)_3$ addition, the difference between irradiated and non-irradiated spectra was clearly visible (Figure S12).

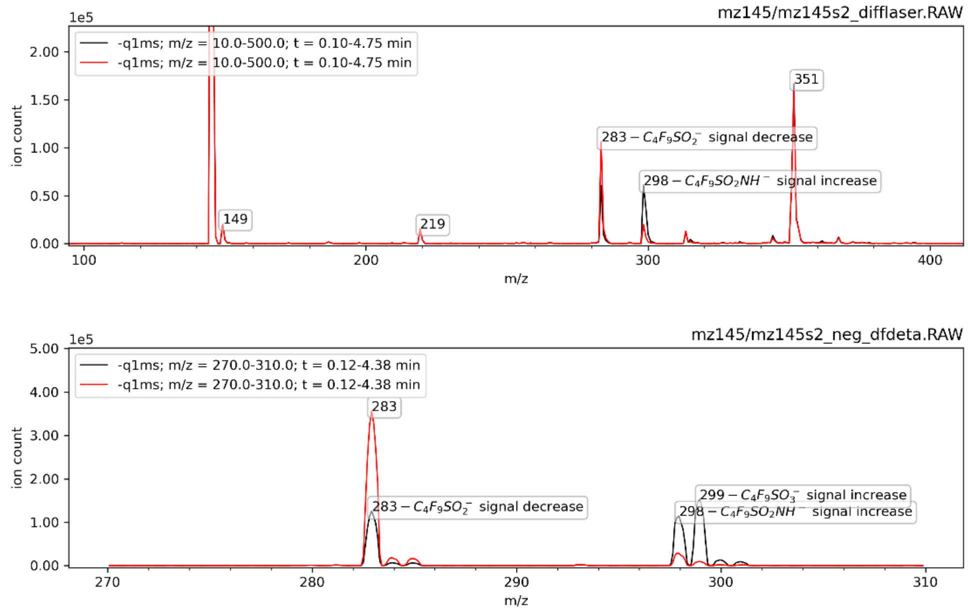

Figure S12. Negative ESI-MS spectrum of NfN_3 and $Ru(bipy)_3(PF_6)_2$ in CH_3CN upon tip-irradiation. Black line represents spectrum with irradiation, red line represents spectrum without irradiation. Conditions: sheath gas pressure 20 psi, auxiliary gas not used, spray voltage 4.5 kV, capillary temperature 250 °C, capillary voltage 0 V, tube lens voltage -60 V (rather soft ESI source conditions). Sample preparation: 8.09 mg of NfN_3 was dissolved in 4 ml of acetonitrile. 100 µl of this concentrated solution was then added to $Ru(bipy)_3$ sample used in the Figure S5.

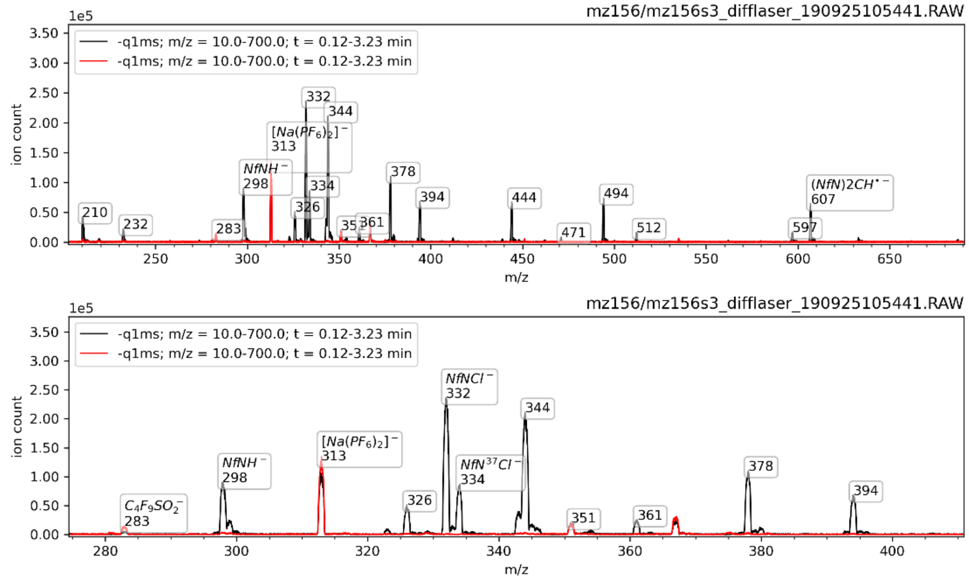

Figure S13. Negative ESI-MS spectrum of $Ru(bipy)_3(PF_6)_2$ and NfN_3 in CD_3CN upon tip-irradiation. Conditions: electron multiplier voltage 1500 V, sheath gas pressure 14 psi, auxiliary gas not used, spray voltage 5.5 kV, capillary temperature 250 °C, capillary voltage 0 V, tube lens voltage -60 V (rather soft ESI source conditions). Sample preparation: 2.95 mg of NfN_3 was dissolved in 0.5 ml of acetonitrile-d_3. 100 µl of this concentrated solution was then added to the sample used in the Figure S10.

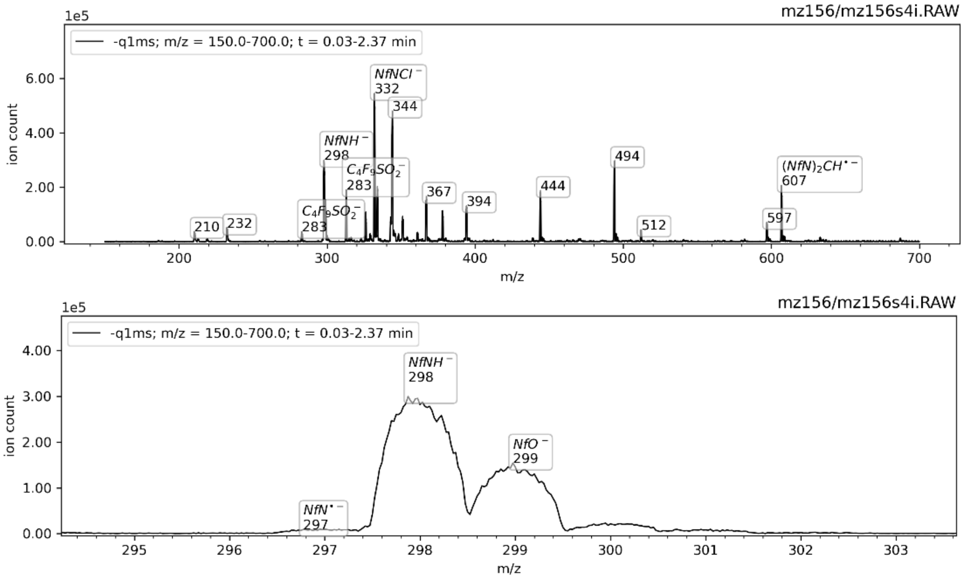

Figure S14. Negative ESI-MS spectrum of NfN_3 and $Ru(bipy)_3(PF_6)_2$ in CD_3CN recorded upon tip-irradiation. Conditions: electron multiplier voltage 1500 V, sheath gas pressure 14 psi, auxiliary gas not used, spray voltage 5.5 kV, capillary temperature 250 °C, capillary voltage 0 V, tube lens voltage -60 V (rather soft ESI source conditions). Sample preparation: 2.95 mg of NfN_3 was dissolved in 0.5 ml of acetonitrile-*d*$_3$. 100 µl of the concentrated solution was then mixed with 0.05 mg of $Ru(bipy)_3$ in 600 µl of acetonitrile-*d*$_3$. The small peak of m/z 297 visible in the spectrum corresponds to the nonaflyl nitrene radical anion.

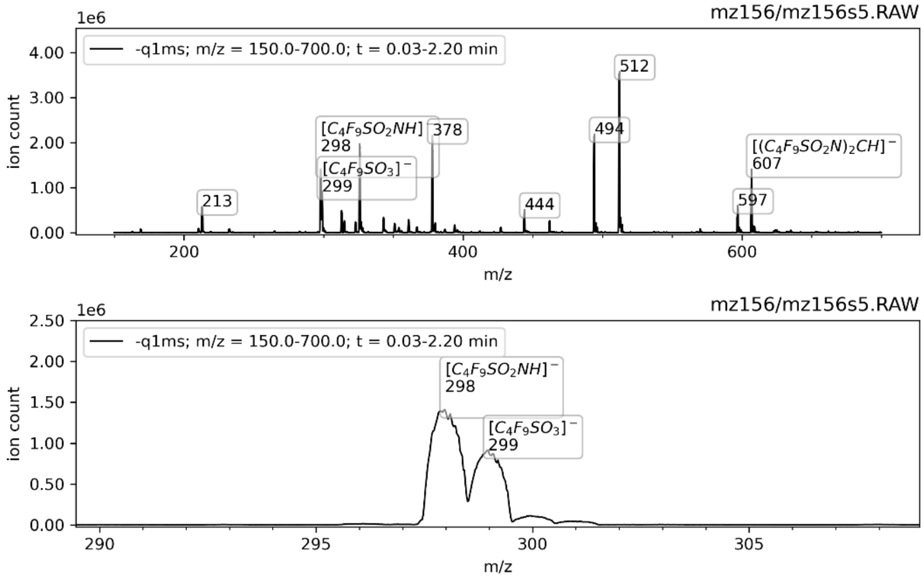

Figure S15. Negative ESI-MS spectrum of NfN_3 and $Ru(bipy)_3(PF_6)_2$ in CD_3CN recorded upon vial-irradiation. Conditions: electron multiplier voltage 1500 V, sheath gas pressure 14 psi, auxiliary gas not used, spray voltage 5.5 kV, capillary temperature 250 °C, capillary voltage 0 V, tube lens voltage -60 V (rather soft ESI source conditions). Sample preparation: 2.95 mg of NfN_3 was dissolved in 0.5 ml of acetonitrile-*d*$_3$. 100 µl of this concentrated solution was then mixed with 0.05 mg of $Ru(bipy)_3$ in 600 µl of acetonitrile-*d*$_3$. The peak of m/z 297 which corresponds to the nonaflyl nitrene radical anion is absent in this spectrum.

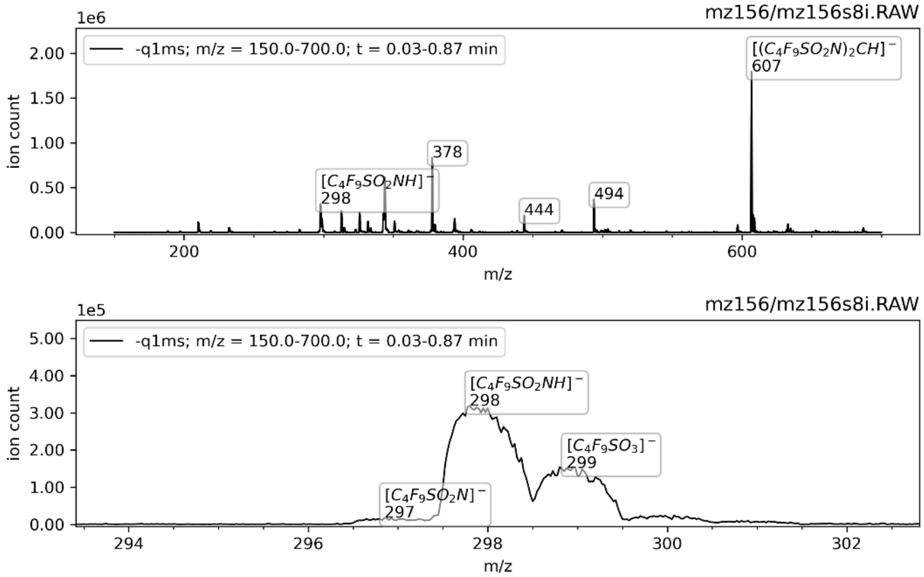

Figure S16. Negative ESI-MS spectrum of NfN_3 and $Ru(bipy)_3(PF_6)_2$ in CD_3CN recorded upon tip-irradiation. Conditions: electron multiplier voltage 1800 V, sheath gas pressure 10 psi, auxiliary gas not used, spray voltage 5.5 kV, capillary temperature 250 °C, capillary voltage 0 V, tube lens voltage -60 V (rather soft ESI source conditions). Sample preparation: 10.3 mg of NfN_3 was dissolved in 0.1 ml of acetonitrile-*d*$_3$. 100 µl of the concentrated solution was then added to 0.1 mg $Ru(bipy)_3$ dissolved in 0.2 ml of acetonitrile-*d*$_3$. There is a small peak of m/z 297 present in the spectrum which corresponds to the nonaflyl nitrene radical anion.

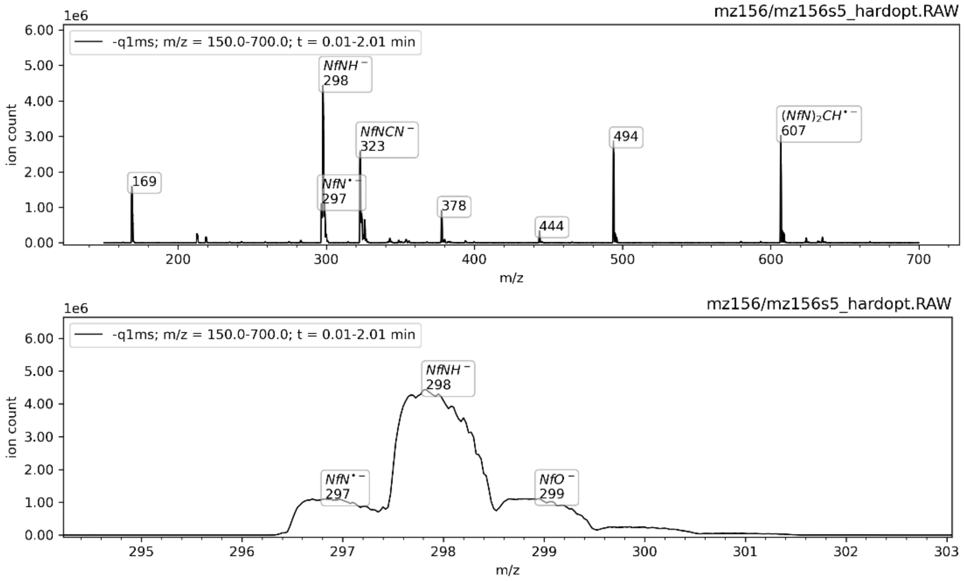

Figure S17. Negative ESI-MS spectrum of $Ru(bipy)_3(PF_6)_2$ and NfN_3 in CD_3CN upon vial-irradiation (LED diodes, 5 minuts). Conditions: electron multiplier voltage 1800 V, sheath gas pressure 20 psi, auxiliary gas not used, spray voltage 7.0 kV, capillary temperature 275 °C, capillary voltage -20 V, tube lens voltage -135 V (rather hard ESI source conditions). Sample: same as in the Figure S15. There is a clearly visible peak of the m/z 297 in the spectrum which corresponds to the nonaflyl nitrene radical anion (**NfN$^{\bullet -}$**).

2.2.3 Fragmentations – Nonaflyl azide derivates

Nonaflyl nitrene radical anion (**NfN**$^{\bullet-}$, m/z 297) visible in the ESI-MS spectra probably originates mainly from the in-source fragmentation of the m/z 607 ion (Figure S19). Its intensity in the source spectrum increases with hardening of the electrospray ionization source conditions (with increasing potential difference in the source region, the generated ions collide with the nitrogen sheath gas with increased collision energy). Parent ion scans for generation of ions with the m/z 297 shows also other potential precursor ions, but the ions with m/z 607 are by-far the most dominant (see Figures S20, S21).

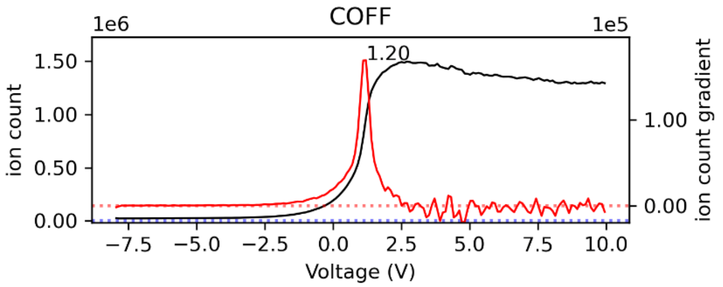

Figure S18. Retarding potential analysis of the kinetic energy distribution of mass selected **NfN**$^{\bullet-}$ (m/z 297) ion. The red curve represents a derivation of the stopping potential curve (black). The collision energy scale in the following MS/MS experiments is set relative to the center of the kinetic energy distribution (red peak).

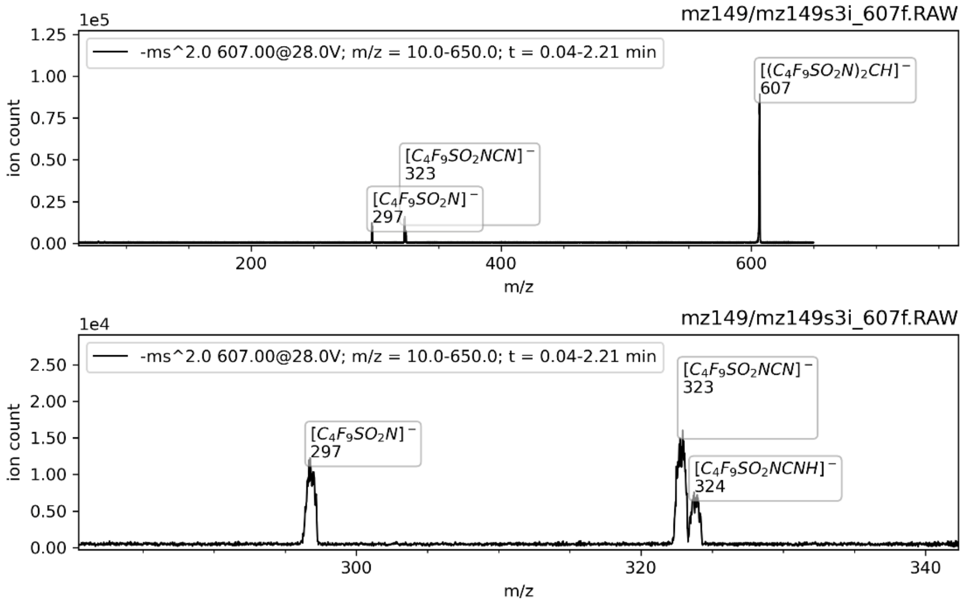

Figure S19. ESI-MS/MS fragmentation spectrum of the m/z 607 parent ion. Conditions: electron multiplier voltage 1800 V, sheath gas pressure 18 psi, auxiliary gas not used, spray voltage 5.0 kV, capillary temperature 250 °C, capillary voltage 0 V, tube lens voltage -80 V, *p*(Xe) = 0.19 mTorr (nominal), E_{cm} = 4.8 eV.

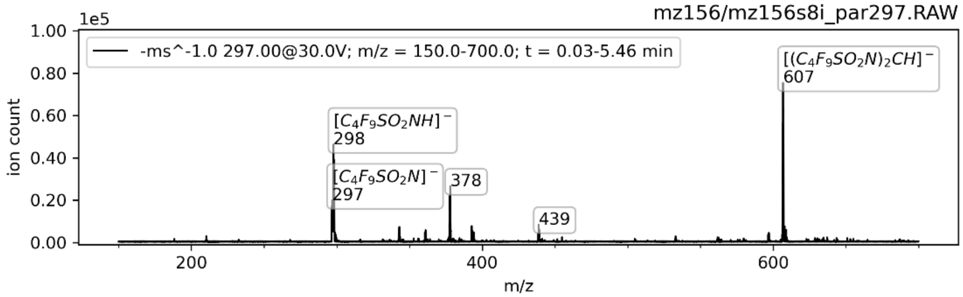

Figure S20. ESI-MS/MS precursor ion scan spectrum of the m/z 297 daughter ion. Conditions: electron multiplier voltage 2500 V, sheath gas pressure 10 psi, auxiliary gas not used, spray voltage 5.5 kV, capillary temperature 250 °C, capillary voltage 0 V, tube lens voltage -60 V, *p*(Xe) = 0.14 mTorr (nominal), E_{cm} = 5.1 eV. The parent ions of the m/z 378 are parent ions of the m/z 298 daughter ion as apparent from the Figure S21. They are present in this spectrum because of the low Q3 (daughter selection quadrupole) resolution.

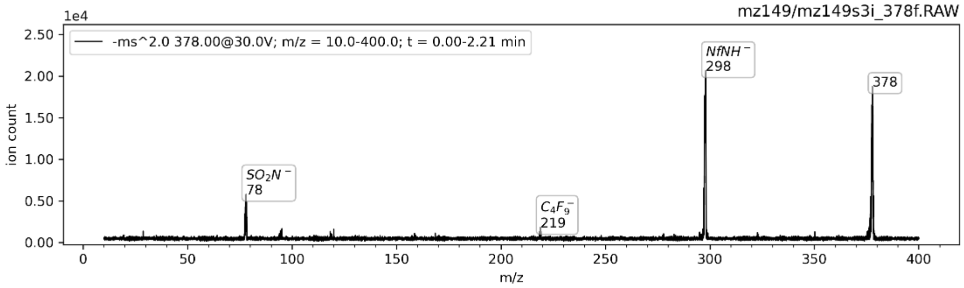

Figure S21. ESI-MS/MS fragmentation spectrum of the m/z 378 parent ion. Conditions: electron multiplier voltage 2200 V, sheath gas pressure 18 psi, auxiliary gas not used, spray voltage 5.0 kV, capillary temperature 250 °C, capillary voltage 0 V, tube lens voltage -70 V, *p*(Xe) = 0.20 mTorr (nominal), E_{cm} = 7.4 eV.

2.2.4 Fragmentation of $Im^{H+}SO_2N^{••}$

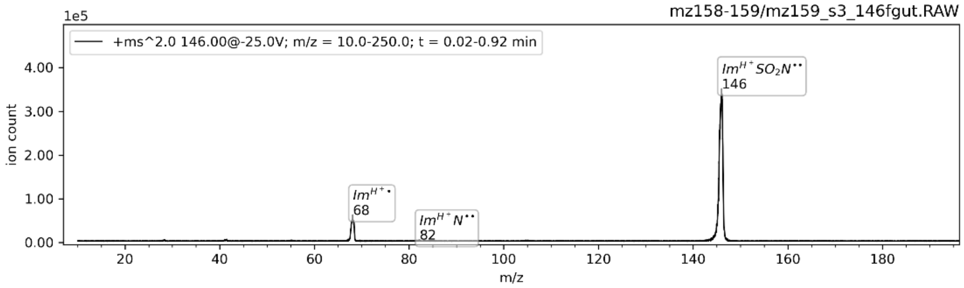

Figure S22. ESI-MS/MS fragmentation spectrum of the m/z 146 parent ion. Conditions: electron multiplier voltage 2000 V, sheath gas pressure 40 psi, auxiliary gas not used, spray voltage 5.5 kV, capillary temperature 250 °C, capillary voltage 0 V, tube lens voltage 80 V, *p*(Xe) = 0.12 mTorr (nominal), E_{cm} = 11.6 eV. The major fragmentation channel is a neutral loss of SO_2N. Peaks corresponding to loss of SO_2 (64 and 82) are almost absent.

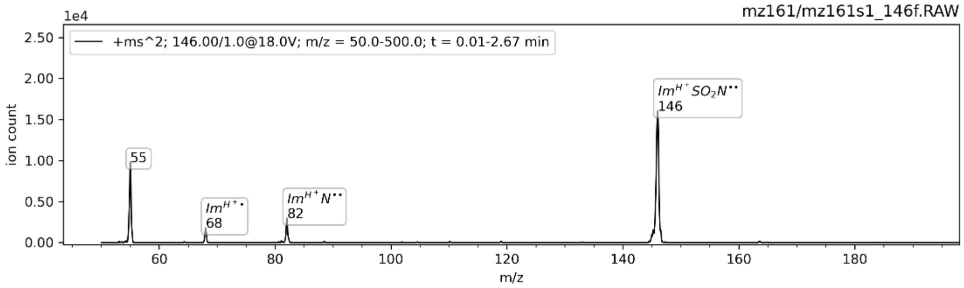

Figure S23. ESI-MS/MS fragmentation spectrum of the m/z 146 parent ion in LCQ Deca (cylindrical ion trap instrument). Conditions: sheat gas flow rate 20 (arb.), auxiliary gas not used, spray voltage 4.5 kV, capillary temperature 250 °C, capillary voltage 20 V, tube lens voltage 130 V, E_{coll}(arb) = 18.

2.2.5 Source spectra acquired without irradiation – Imidazoliumsulfonyl azide

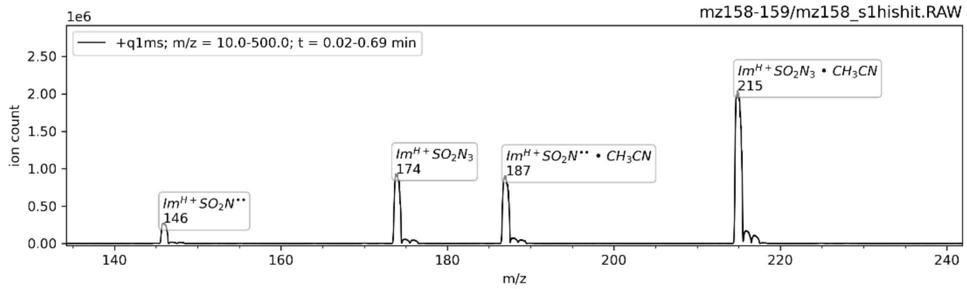

Figure S24. ESI-MS spectrum of the imidazole sulfonyl azide – hard ESI source conditions. Conditions: source voltage 5.5 kV, capillary temperature 250 °C, capillary voltage 0 V, tube lens voltage 80 V. The isotopic pattern correlates with one sulfur atom in the molecule.

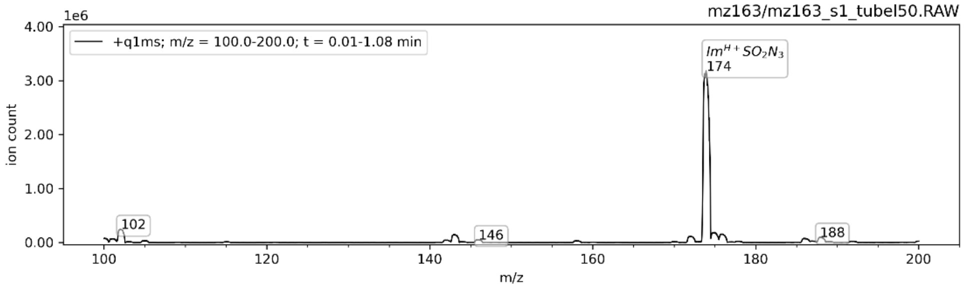

Figure S25. ESI-MS spectrum of imidazole sulfonyl azide – soft ESI source conditions. Conditions: source voltage 5.5 kV, capillary temperature 250 °C, capillary voltage 0 V, tube lens voltage 50 V.

2.2.6 Source spectra acquired with irradiation – imidazole-1-sulfonyl azide

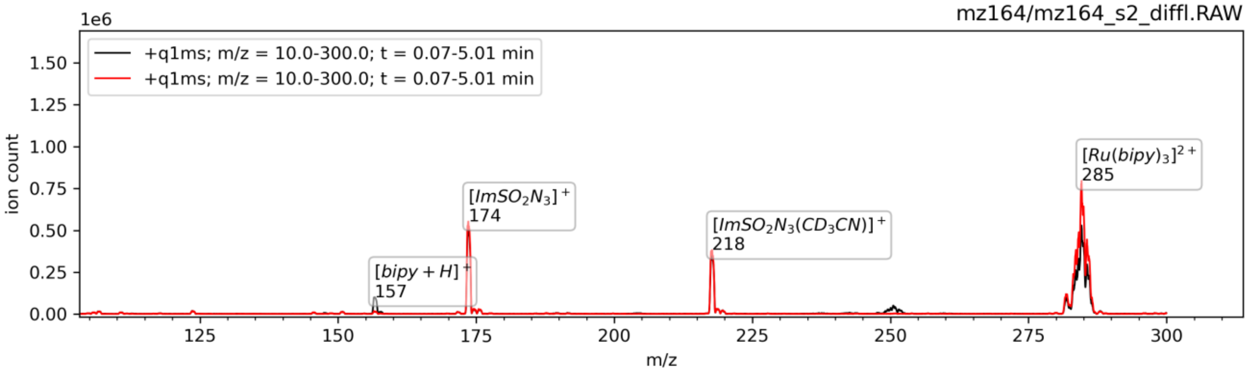

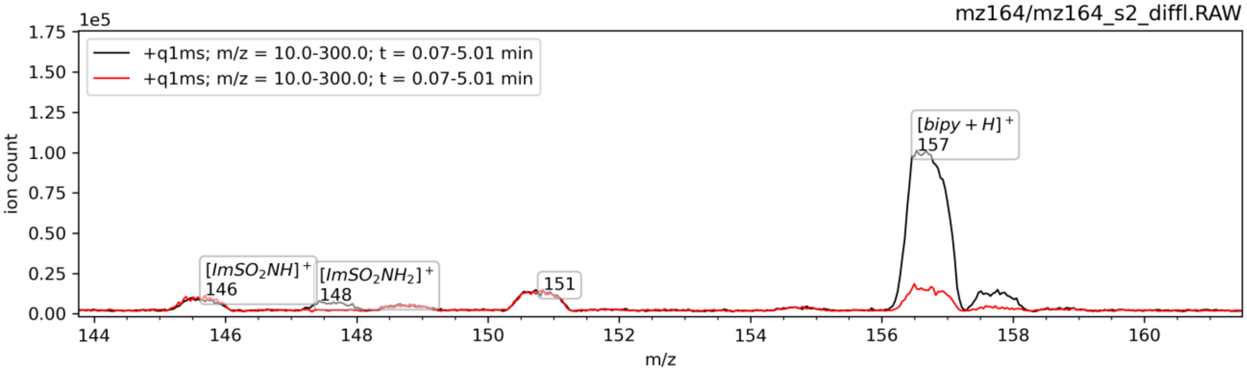

Figure S26. Positive ESI-MS spectrum of $Ru(bipy)_3(PF_6)_2$ and $ImSO_2N_3$ in CD_3CN upon tip-irradiation. Conditions: electron multiplier voltage 1400 V, sheath gas pressure 10 psi, auxiliary gas not used, spray voltage 5.5 kV, capillary temperature 250 °C, capillary voltage 0 V, tube lens voltage 50 V (rather soft ESI source conditions). Sample: 50 µM/l $Ru(bipy)_3(PF_6)_2$ and 320 µM/l $ImSO_2N_3$ in acetonitrile-*d*$_3$. There is no difference in intensity of the $ImSO_2N_3$ peak (m/z 146) between irradiated and non-irradiated spectrum.

2.3 Gas-phase reactivity of selected ions (S27-S68)

2.3.1 Conditions used for generation of reactive ions and their MS spectra

For **NfN$^{•-}$** ESI-MS/MS reactivity experiments **NfN$^{•-}$** (m/z 297) was generated by in-source fragmentation of (**NfN)$_2$CH$^{•-}$** (m/z 607). This ion is visible when irradiated mixture of nonaflyl azide (**NfN$_3$**) and $Ru(bipy)_3^{2+}$ is sprayed. The peak of (**NfN)$_2$CH$^{•-}$** (m/z 607) is more abundant when concentrated solution is irradiated. We thus irradiated a concentrated solution which was then diluted prior to the use in the ESI-MS. Figure S27 illustrates the spectrum of a sample used for generation of **NfN$^{•-}$**.

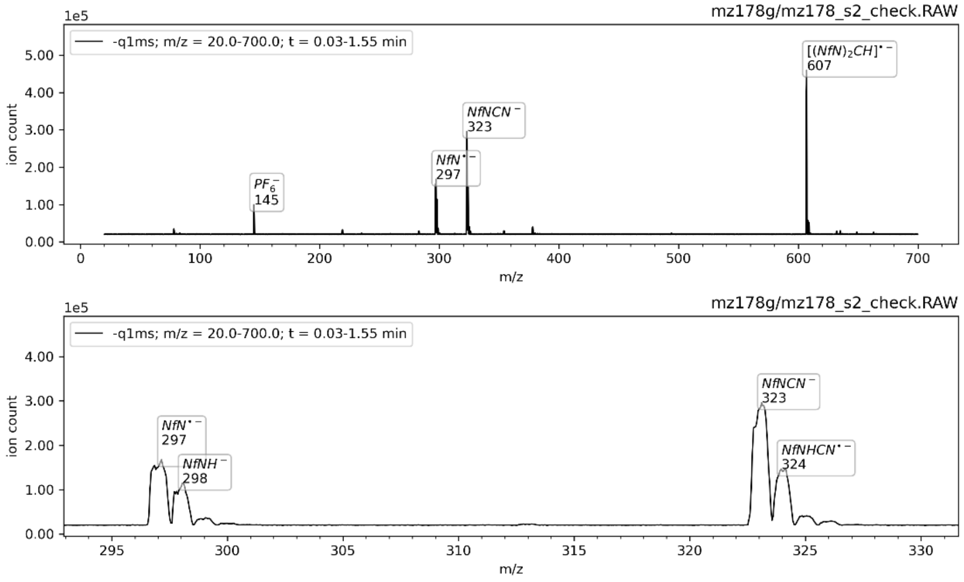

Figure S27. ESI-MS spectrum of the vial-irradiated solution of nonaflyl azide (**NfN$_3$**) and Ru(bipy)$_3$(PF$_6$)$_2$ in CD$_3$CN (200 µl). Sample preparation: solution of nonaflyl azide (**NfN$_3$**, 78.9 mmol/l) and Ru(bipy)$_3$(PF$_6$)$_2$ (1.3 mmol/l) in CD$_3$CN (s200 µl) was irradiated for 5 minutes by 4x700mA diodes (Luxeon Rebel, 445nm, Figure S2). Then it was diluted with CH$_3$CN (2ml) and sprayed. Conditions: sheath gas pressure 12 psi, auxiliary gas pressure 40 psi, low sample flow (overpressure used), sprayed from a close proximity without discharge, source voltage 5 kV, capillary temperature 250 °C, capillary voltage -60 V, tube lens voltage -120 V. The peak of **NfN$^{\bullet-}$** (m/z 297) is very intense under these conditions.

For **Im^{H+}SO$_2$N$^{\bullet\bullet}$** ions ESI-MS/MS reactivity experiments imidazole-1-sulfonyl nitrene (**Im^{H+}SO$_2$N$^{\bullet\bullet}$**, m/z 146) ion was generated by in-source fragmentation of the **Im^{H+}SO$_2$N$_3$** (*m/z* 174). **Im^{H+}SO$_2$N$_3$** hydrogensulfate solution in acetonitrile has been used as an analyte.

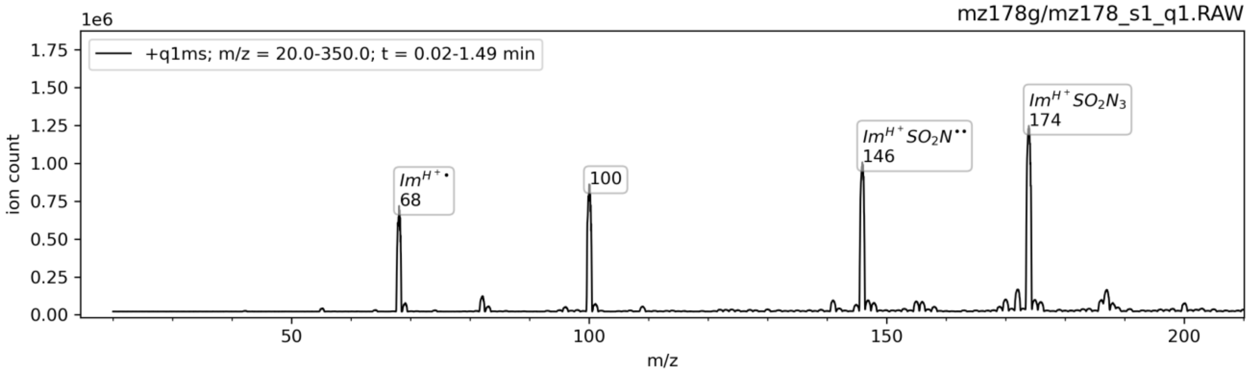

Figure S28. ESI-MS spectrum of a solution of imidazole-1-sulfonyl nitrene hydrogensulfate (**Im^{H+}SO$_2$N$_3$***HSO$_4^-$, 302 µmol/l) in CH$_3$CN. Conditions: sheath gas pressure 12 psi, auxiliary gas pressure 40 psi, low sample flow (overpressure used), sprayed from a close proximity without discharge, source voltage 5 kV, capillary temperature 250 °C, capillary voltage 15 V, tube lens voltage 50 V. The peak of **Im^{H+}SO$_2$N$^{\bullet\bullet}$** (m/z 146) is intense under these conditions.

For **Im^{H+}SO$_2$NH$^{\bullet}$** ion ESI-MS/MS reactivity experiments imidazole-1-sulfonyl amidyl radical (**Im^{H+}SO$_2$NH$^{\bullet}$**, m/z 147) was generated by spraying **Im^{H+}SO$_2$N$_3$** hydrogensulfate solution. Conditions were slightly changed from the one used for the generation of the **Im^{H+}SO$_2$N$^{\bullet\bullet}$** ion. In this case, auxiliary gas flow was set almost to zero and a tissue wetted with tetrahydrofuran (THF) was placed into the ESI-source. THF vapors served as a hydrogen source to convert **Im^{H+}SO$_2$N$^{\bullet\bullet}$** to **Im^{H+}SO$_2$NH$^{\bullet}$**. A syringe pump filled with THF was connected through HPLC plastic tube to this tissue to constantly wet it with a fresh THF. Figure S29 shows the source spectrum recorded under these conditions.

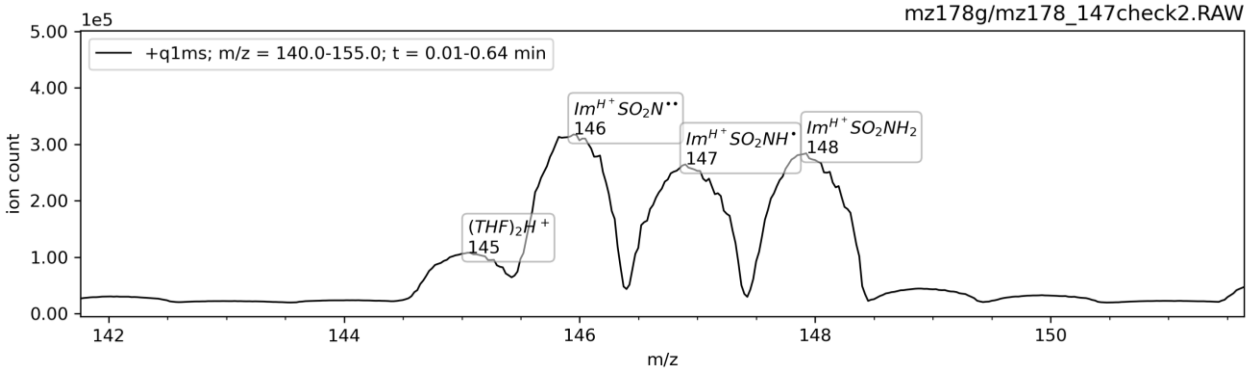

Figure S29. ESI-MS spectrum of a solution of imidazole-1-sulfonyl nitrene hydrogensulfate (**$Im^{H+}SO_2N_3$***HSO_4^-, 302 µmol/l) in CH_3CN. A tissue placed in the ESI source was wetted constantly with 0.4 ml/h flow of THF. Conditions: sheath gas pressure 12 psi, auxiliary gas pressure almost 0 psi, low sample flow (overpressure used), sprayed from a close proximity without discharge, source Voltage 5 kV, capillary temperature 250 °C, capillary voltage 15 V, tube lens voltage 50 V. The peak of **$Im^{H+}SO_2NH^{\bullet}$** (m/z 147) is relatively intense, but still slightly contaminated with ^{13}C isomer of **$Im^{H+}SO_2N^{\bullet\bullet}$** (m/z 146). As this contamination can lead to misinterpretation of the observed reactivity, the source spectrum was checked routinely between the reactivity experiments, to ensure that indeed the reactivity of **$Im^{H+}SO_2NH^{\bullet}$** and not ^{13}C- **$Im^{H+}SO_2N^{\bullet\bullet}$** is being recorded. In a case that the THF flow was increased too much, the signals of **$Im^{H+}SO_2NH_2$** and **$(THF)_2H^+$** dominated the whole spectrum.

2.3.2 Gas-phase reactivity of $NfN^{\bullet-}$

Nonaflyl nitrene radical-anion (**$NfN^{\bullet-}$**, m/z 297) was unreactive towards: ethane, cyclohexane, tetrahydrofuran, ethylene, cyclooctene, acetylene, acetone, water and acetonitrile. It reacted with ethanethiol, ethanethiol-d and acetylacetone.

2.3.2.1 Reactivity with ethanethiol and ethanethiol-d1

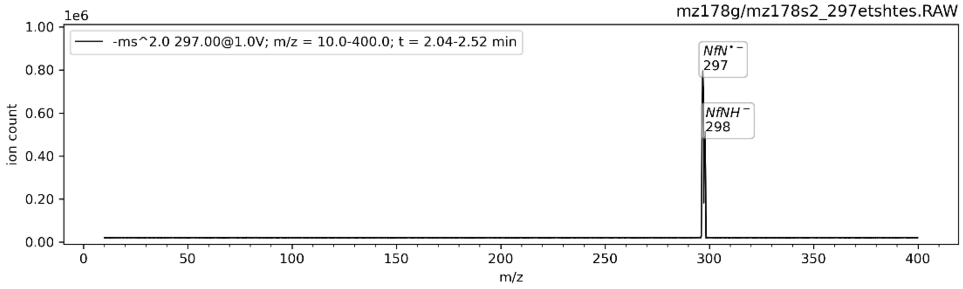

Figure S30. ESI-MS/MS spectrum of the gas-phase reaction of the **$NfN^{\bullet-}$** (m/z 297) parent ion with ethanethiol (C_2H_5SH). Conditions: source conditions were similar to the one used in the Figure S27, $p(C_2H_5SH)$ = 0.40 mTorr (nom.), E_{CM} = 0 eV.

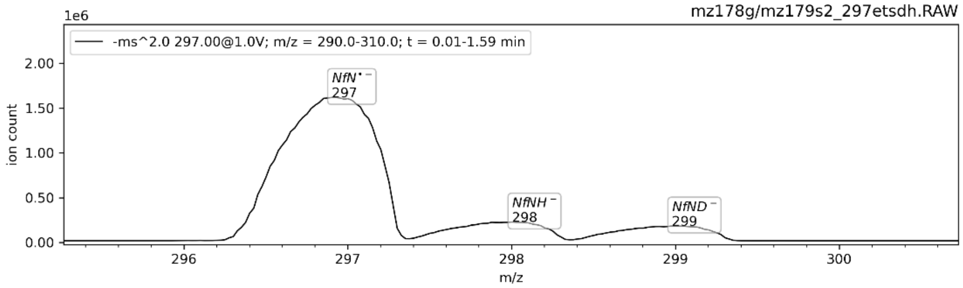

Figure S31. ESI-MS/MS spectrum of the gas-phase reaction of the **$NfN^{\bullet-}$** (m/z 297) parent ion with ethanethiol (C_2H_5SH / C_2H_5SD). Conditions: source conditions were similar to the one used in the Figure S27, $p(C_2H_5SH/\ C_2H_5SD)$ = 0.30 mTorr (nom.), E_{CM} = 0 eV. The collision gas in this case were vapors from a tube containing ethanethiol/D_2O emulsion, thus ratio of C_2H_5SH : C_2H_5SD is unknown. However, it is definitely apparent that the hydrogen atom bound to sulfur is being transferred.

2.3.2.2 *Reactivity with acetylacetone*

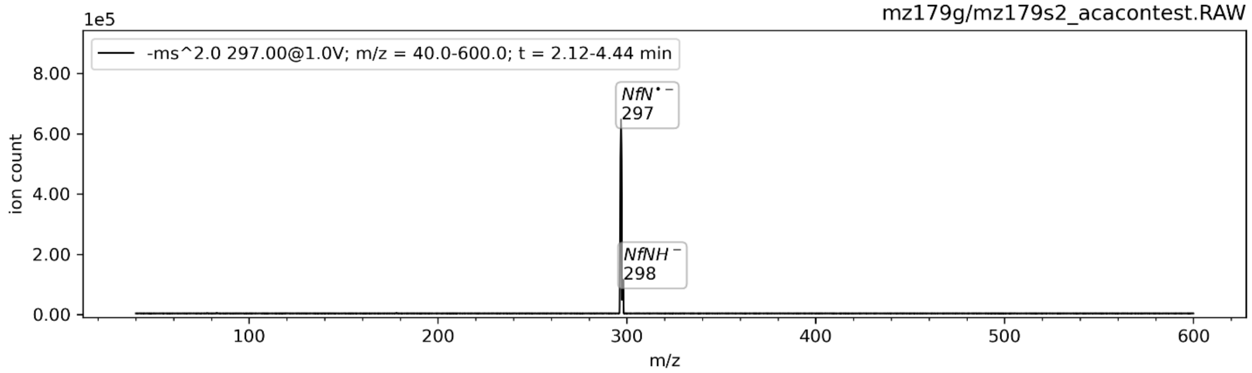

Figure S32. ESI-MS/MS spectrum of the gas-phase reaction of the **NfN$^{•-}$** (m/z 297) parent ion with acetylacetone ($(CH_3CO)_2CH_2$). Conditions: source conditions were similar to the one used in the Figure S27, *p*(acetylacetone) = 0.44 mTorr (nom.), E_{CM} = 0 eV.

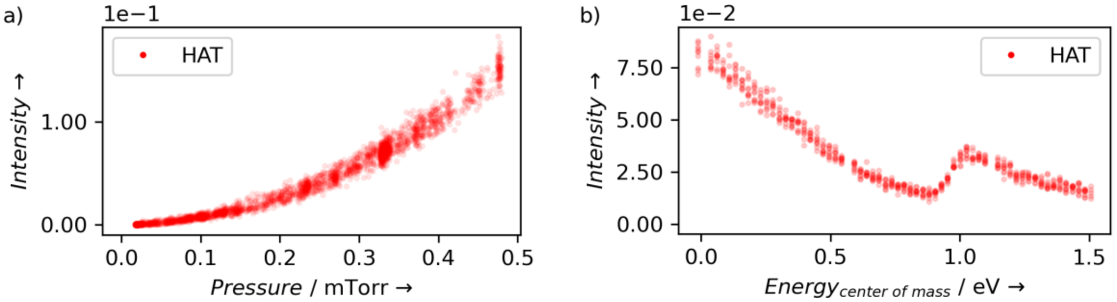

Figure S33. Pressure (a, E_{CM} = *0*) and collision-energy (b, *p*(acetylacetone) = 0.33 mTorr) dependencies of the hydrogen atom transfer (HAT) channel relative intensity in the gas-phase reactivity of (**NfN$^{•-}$** m/z 297) parent ion with acetylacetone. Conditions: source conditions were similar to the one used in the Figure S27. The intensity of the channel has been plotted relatively to sum of selected ions intensities – m/z 297 and m/z 298 in this case. Other channels were neglected. Background was subtracted. Collision-energy dependence of the intensity suggests that there are two gas-phase reactions taking place. One at zero collision-energy and one at collision energy of approximately 1 eV. We speculate, that these two reactions might correspond to gas phase reactions of the keto and the enol form of acetylacetone.

2.3.2.3 *Reactivity with 1,4-cyclohexadiene*

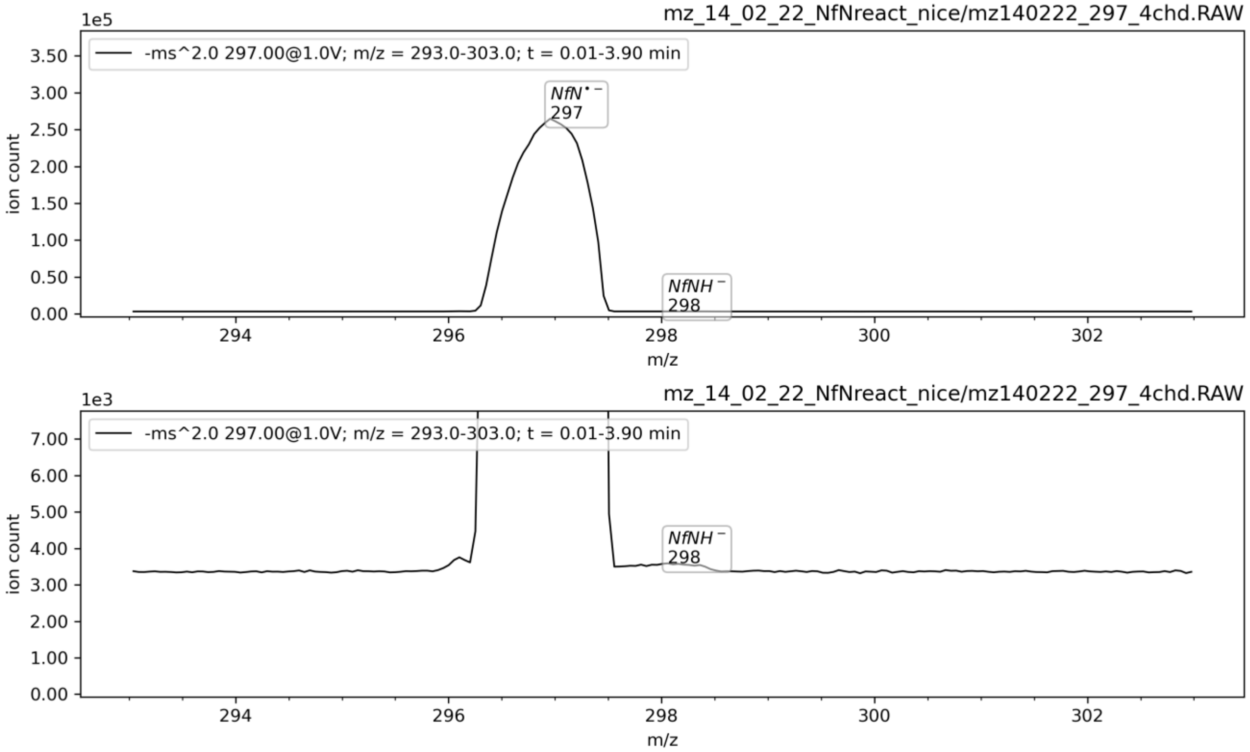

Figure S34. ESI-MS/MS spectrum of the gas-phase reaction of the **NfN**$^{\bullet-}$ (m/z 297) parent ion with 1,4-cyclohexadiene. Conditions: source conditions were similar to the one used in the Figure S27, *p*(1,4-cyclohexadiene) = 0.33 mTorr (nom.), E_{CM} = 0 eV. There is a very small peak of M+1 which corresponds to **NfNH**$^{-}$, however due to its very small intensity its inconclusive, if it stems from reactivity of **NfN**$^{\bullet-}$ with 1,4-cyclohexadiene, or from reaction of **NfN**$^{\bullet-}$ with unknown trace impurity present in the collision cell during the experiment.

2.3.2.4 *Reactivity with 1,3-cyclohexadiene*

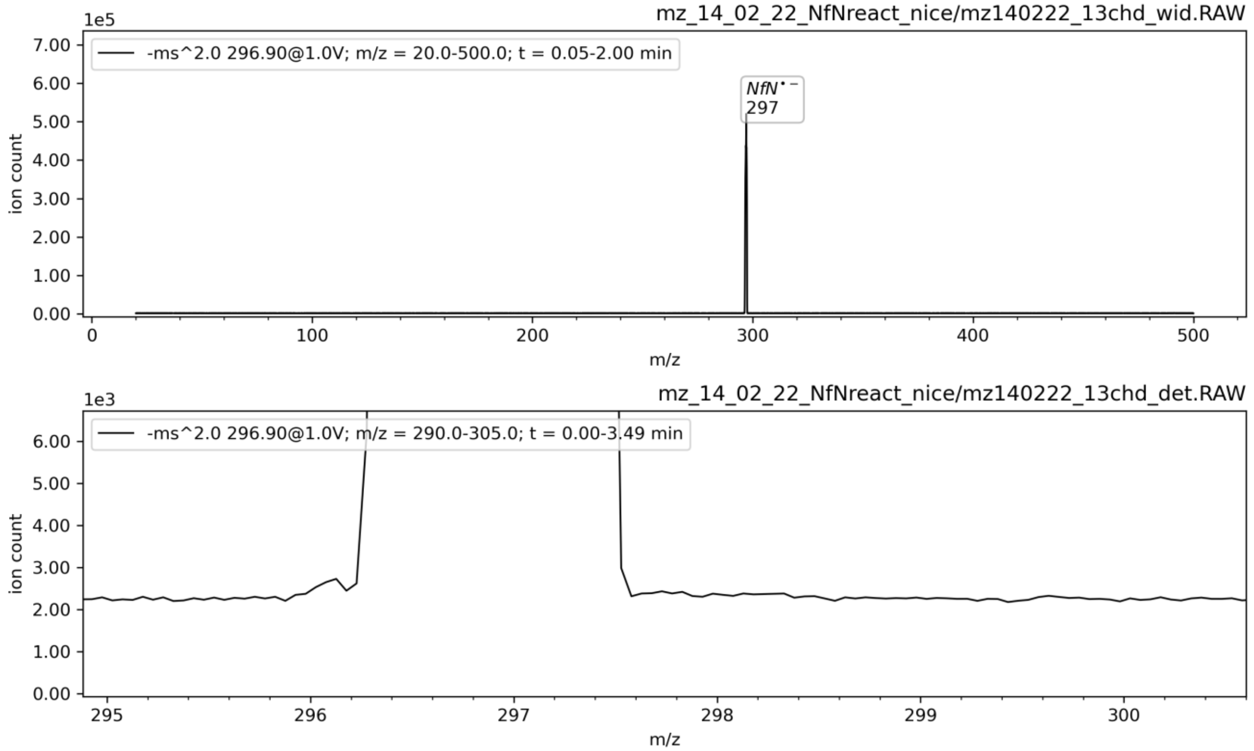

Figure S35. ESI-MS/MS spectrum of the gas-phase reaction of the **NfN$^{•-}$** (m/z 297) parent ion with 1,3-cyclohexadiene. Conditions: source conditions were similar to the one used in the Figure S27, *p*(1,3-cyclohexadiene) = 0.37 mTorr (nom.), E_{CM} = 0 eV. There is no apparent peak of **NfNH$^-$**, which implies absence of gas-phase reaction in this case.

2.3.3 Gas-phase reactivity of $Im^{H+}SO_2N^{••}$

The imidazole-1-sulfonyl nitrene (**$Im^{H+}SO_2N^{••}$**, m/z 146) was unreactive towards water, nitrous oxide and methane. It reacted with: ethanethiol, acetylacetone, 1,4-cyclohexadiene, 1,3-cyclohexadiene, benzene, acetone, methanol, tetrahydrofuran, cyclohexane and ethane. The reactivity was dominated mainly by hydrogen atom transfer. The nature of the hydrogen atom transfer (ECPT, PCET, concerted HAT) cannot be derived from our experiments.

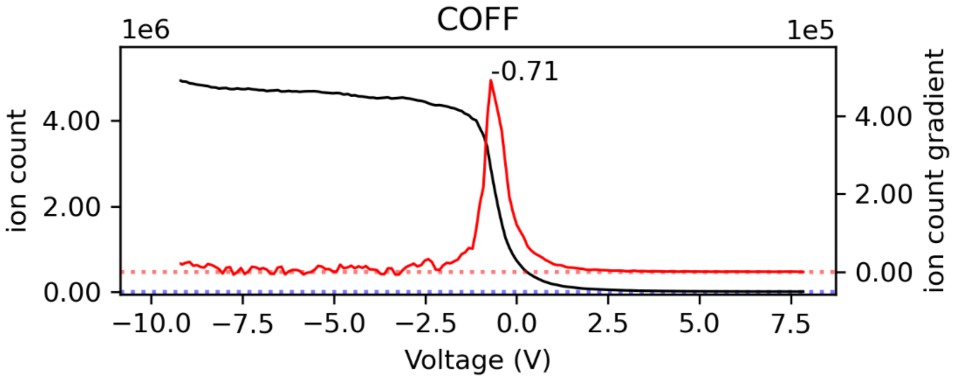

Figure S36. Retarding potential analysis of the kinetic energy distribution of the mass selected **$Im^{H+}SO_2N^{••}$** (m/z 146) ion. The red curve represents a derivation of the stopping potential curve (black). The collision energy scale in the following MS/MS experiments is set relative to the center of the kinetic energy distribution (red peak).

2.3.3.1 *Reactivity with ethanethiol and ethanethiol-d1*

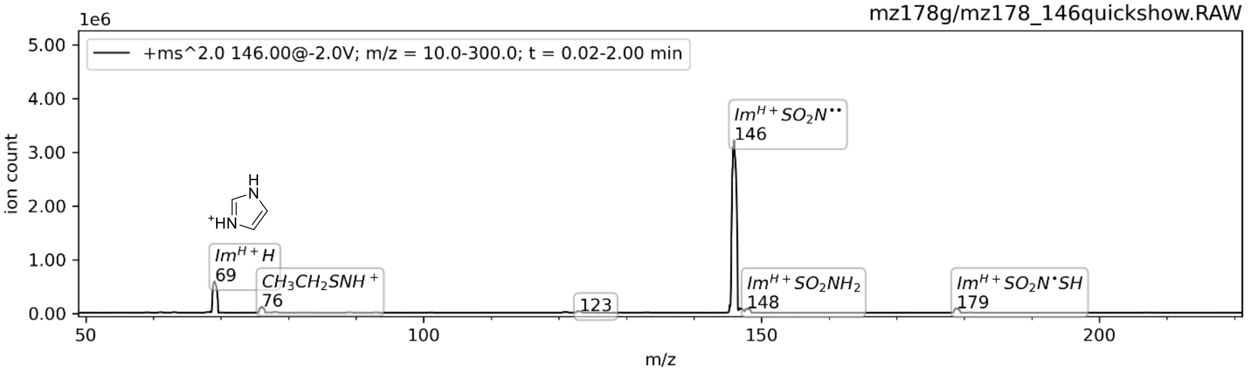

Figure S37. ESI-MS/MS spectrum of the gas-phase reaction of $\mathbf{Im^{H+}SO_2N^{••}}$ (m/z 146) parent ion with ethanethiol (C_2H_5SH). Conditions: source conditions were similar to the one used in the Figure S28, *p*(ethanethiol) = 0.09-0.12 mTorr (nom.), E_{CM} = 0 eV. The assignment of the m/z 179 and the m/z 76 peaks is speculative.

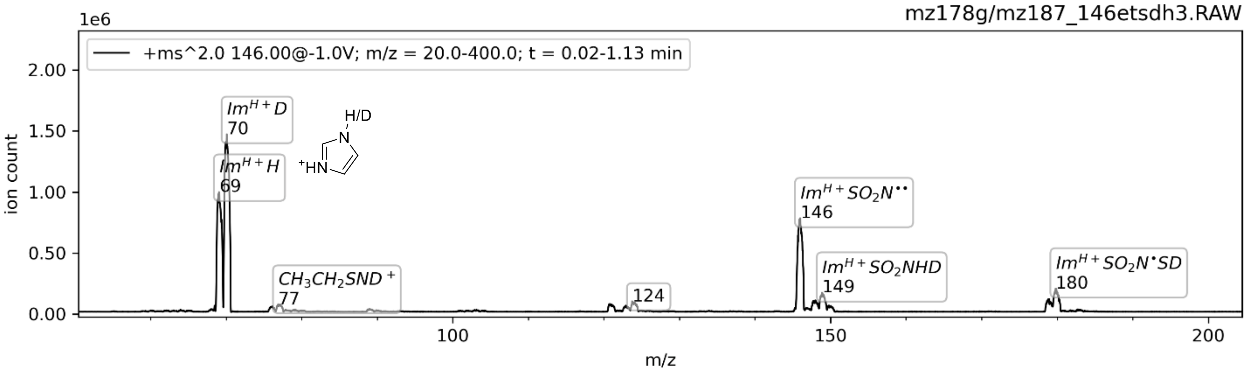

Figure S38. ESI-MS/MS spectrum of the gas-phase reaction of $\mathbf{Im^{H+}SO_2N^{••}}$ (m/z 146) parent ion with ethanethiol (C_2H_5SH/ C_2H_5SD). Conditions: source conditions were similar to the one used in the Figure S28, *p*(C_2H_5SH/ C_2H_5SD) = 0.27 mTorr (nom.), E_{CM} = 0 eV. Collision gas were vapors from a tube containing ethanethiol/D_2O emulsion, thus ratio of C_2H_5SH : C_2H_5SD is unknown. However, it is definitely apparent that the hydrogen atom bound to sulfur is being transferred. The assignment of the m/z 180 and the m/z 77 peaks is speculative.

2.3.3.2 *Reactivity with acetylacetone*

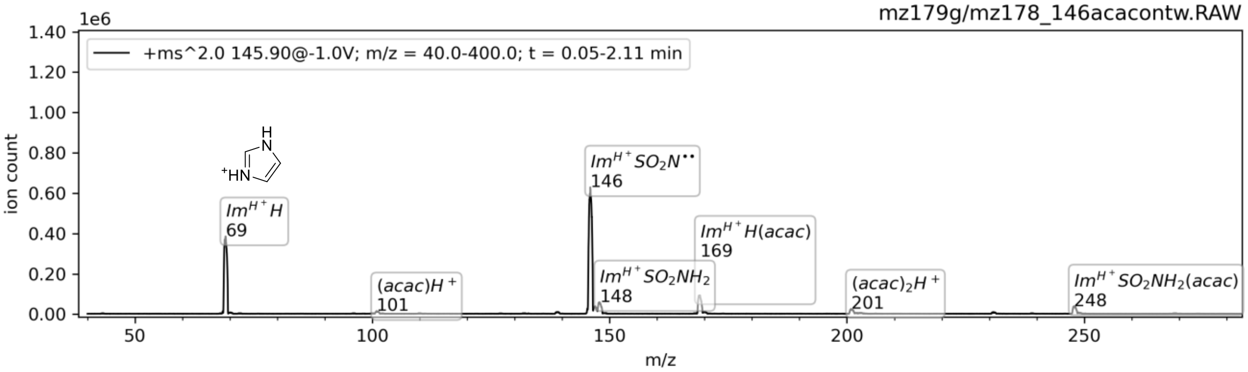

Figure S39. ESI-MS/MS spectrum of the gas-phase reaction of $\mathbf{Im^{H+}SO_2N^{••}}$ (m/z 146) parent ion with acetylacetone ($(CH_3CO)_2CH_2$). Conditions: source conditions were similar to the one used in the Figure S28, *p*(acetylacetone) = 0.05 mTorr (nom.), E_{CM} = 0 eV.

2.3.3.3 *Reactivity with 1,4-cyclohexadiene*

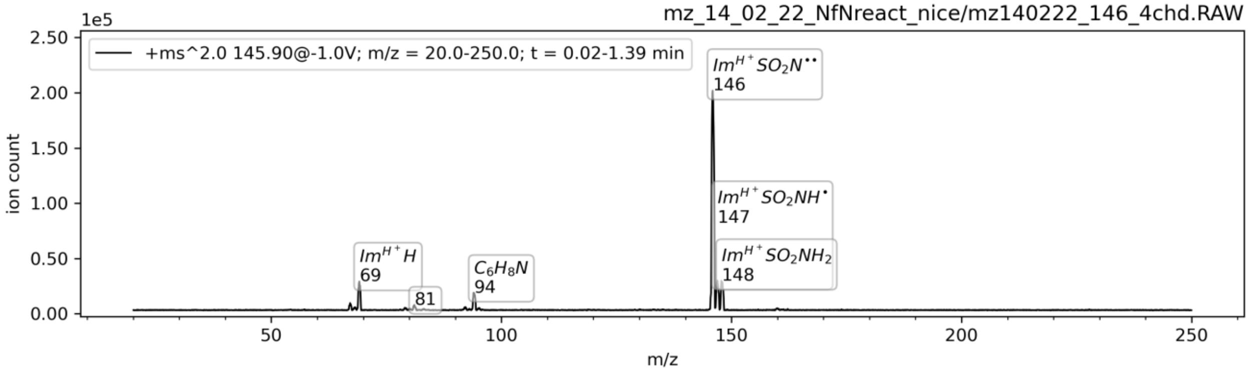

Figure S40. ESI-MS/MS spectrum of the gas-phase reaction of **$Im^{H+}SO_2N^{\bullet\bullet}$** (m/z 146) parent ion with 1,4-cyclohexadiene (C_6H_8). Conditions: source conditions were similar to the one used in the Figure S28, *p*(1,4-cyclohexadiene) = 0.08 mTorr (nom.), E_{CM} = 0 eV.

2.3.3.4 *Reactivity with 1,3-cyclohexadiene*

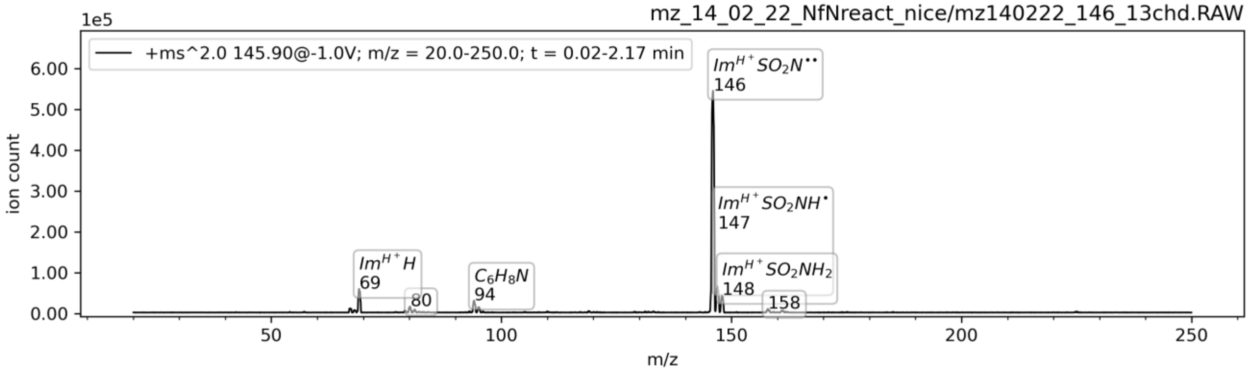

Figure S41. ESI-MS/MS spectrum of the gas-phase reaction of **$Im^{H+}SO_2N^{\bullet\bullet}$** (m/z 146) parent ion with 1,3-cyclohexadiene (C_6H_8). Conditions: source conditions were similar to the one used in the Figure S28, *p*(1,3-cyclohexadiene) = 0.07 mTorr (nom.), E_{CM} = 0 eV.

2.3.3.5 *Reactivity with acetone*

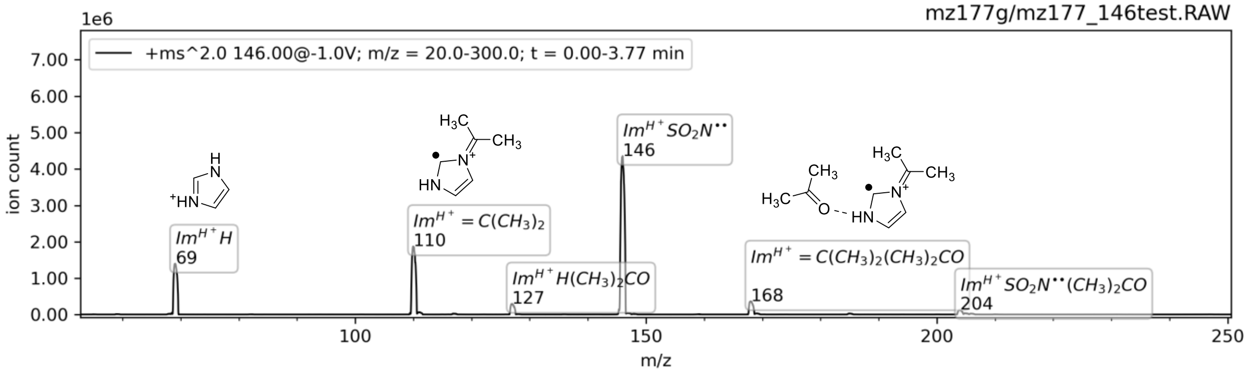

Figure S42. ESI-MS/MS spectrum of the gas-phase reaction of **$Im^{H+}SO_2N^{\bullet\bullet}$** (m/z 146) parent ion with acetone ($(CH_3)_2CO$). Conditions: source conditions were similar to the one used in the Figure S28, *p*(acetone) = 0.14 mTorr (nom.), E_{CM} = 0 eV.

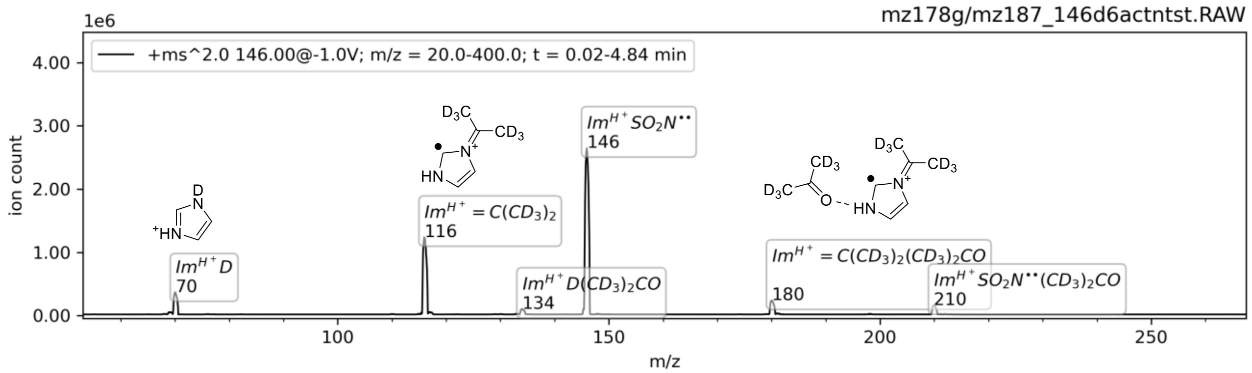

Figure S43. ESI-MS/MS spectrum of the gas-phase reaction of **$Im^{H+}SO_2N^{\bullet\bullet}$** (m/z 146) parent ion with acetone-*d6* ($(CD_3)_2CO$). Conditions: source conditions were similar to the one used in the Figure S28, *p*(acetone-d6) = 0.11 mTorr (nom.), E_{CM} = 0 eV.

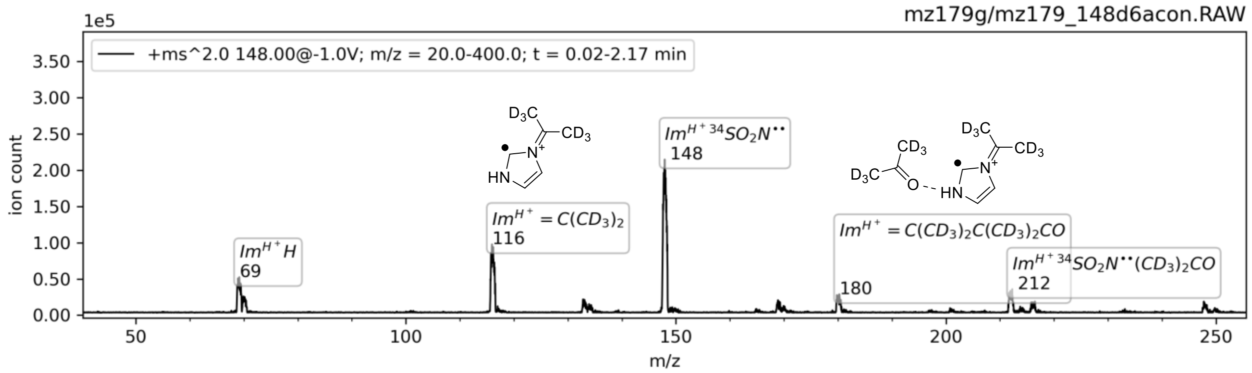

Figure S44. ESI-MS/MS spectrum of the gas-phase reaction of **$Im^{H+\,34}SO_2N^{\bullet\bullet}$** (m/z 148) parent ion with acetone-*d6* ($(CD_3)_2CO$). Conditions: source conditions were similar to the one used in the Figure S28, *p*(acetone-d6) = 0.14 mTorr (nom.), E_{CM} = 0 eV. Shifts in m/z caused by different isotopes made the peaks assignment possible for both this Figure and for the Figure S43.

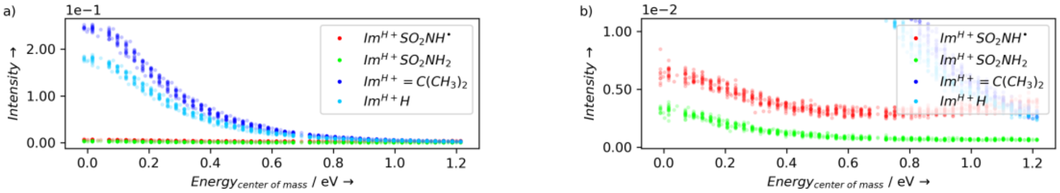

Figure S45. Collision energy dependece spectrum a) of the selected channels of the gas-phase reaction of **$Im^{H+\,34}SO_2N^{\bullet\bullet}$** (m/z 148) parent ion with acetone ($(CH_3)_2CO$). Spectrum b) is an inset. Conditions: source conditions were similar to the one used in the Figure S28, *p*(acetone) = 0.14 mTorr. Please refer to the Figure S43 for the peak assignment.

2.3.3.6 Reactivity with methanol

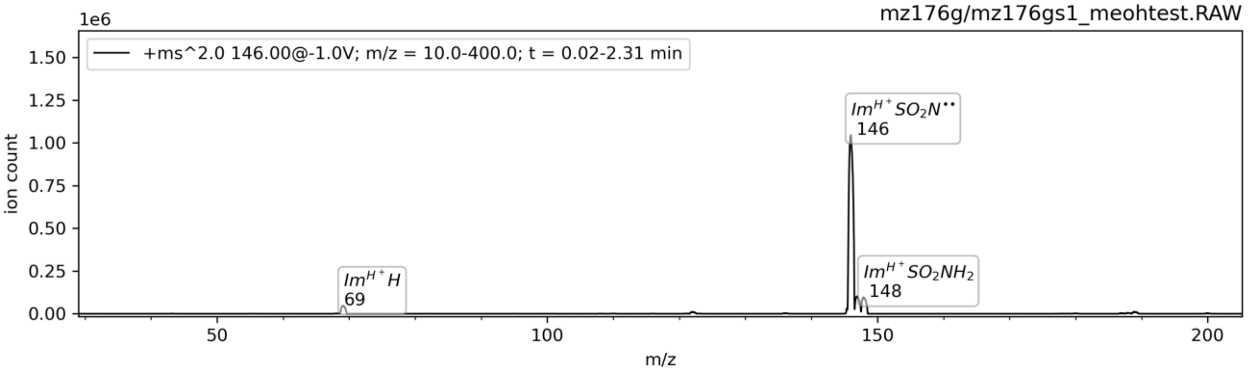

Figure S46. ESI-MS/MS spectrum of the gas-phase reaction of **$Im^{H+}SO_2N^{\bullet\bullet}$** (m/z 146) parent ion with methanol-d_0 (CH_3OH). Conditions: source conditions were similar to the one used in the Figure S28, *p*(methanol) = 0.12 mTorr (nom.), E_{CM} = 0 eV. Signals of the hydrogen atom transfer / fragmentation (HAT+FRG, m/z 69), hydrogen atom transfer (HAT, m/z 147) and double hydrogen atom transfer (2x HAT, m/z 148) are visible in the spectrum.

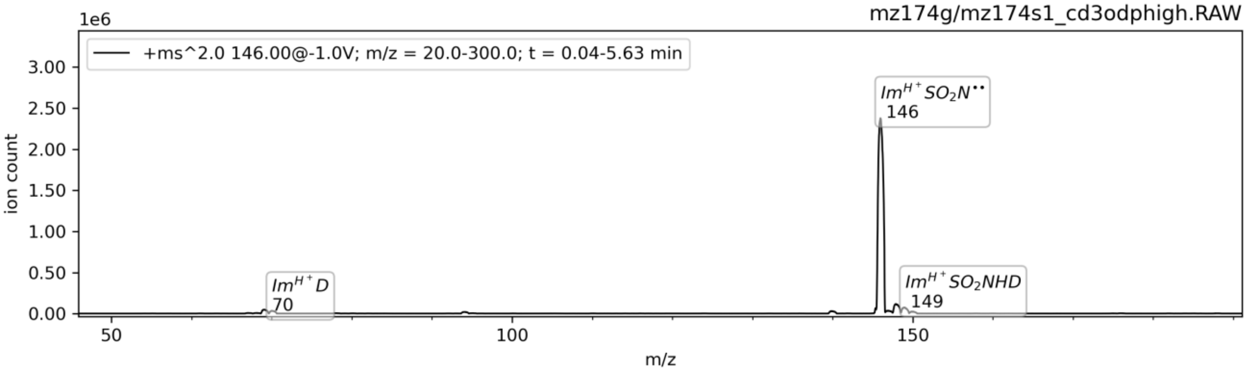

Figure S47. ESI-MS/MS spectrum of the gas-phase reaction of **$Im^{H+}SO_2N^{\bullet\bullet}$** (m/z 146) parent ion with methanol-d_3/d_4 (CD_3OH/CD_3OD). Conditions: source conditions were similar to the one used in the Figure S28, *p*(CD_3OH/CD_3OD) = 0.13 mTorr (nom.), E_{CM} = 0 eV. Signals of hydrogen atom transfer combined with fragmentation (HAT+FRG, m/z 69), deuterium atom transfer combined with fragmentation (DAT+FRG, m/z 70), hydrogen atom transfer (HAT, m/z 147), double hydrogen atom transfer/deuterium atom transfer (2x HAT/DAT, m/z 148), hydrogen and deuterium atom transfer (HAT+DAT, m/z 149) and double deuterium atom transfer (2x DAT, m/z 150) are visible in the spectrum.

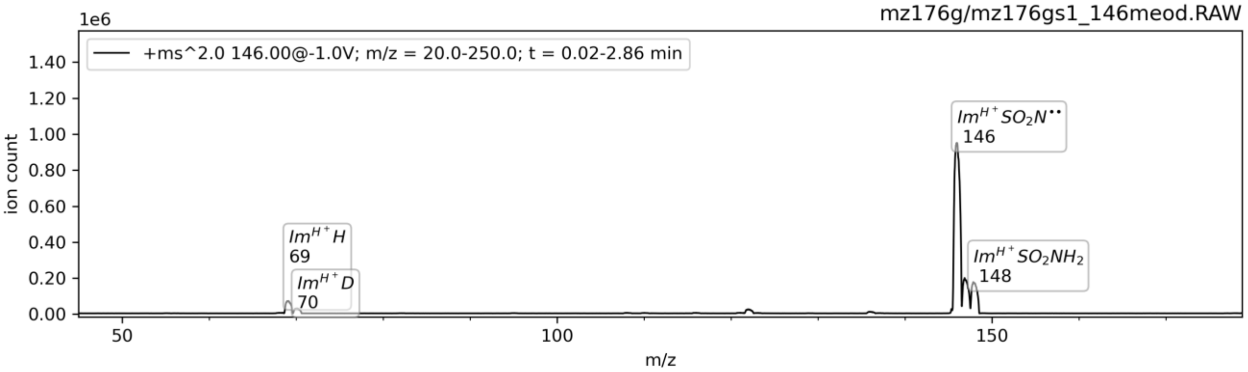

Figure S48. ESI-MS/MS spectrum of the gas-phase reaction of **$Im^{H+}SO_2N^{\bullet\bullet}$** (m/z 146) parent ion with methanol-d_0/d_1 (CH_3OH/CH_3OD). Conditions: source conditions were similar to the one used in the Figure S28, *p*(CH_3OH/CH_3OD) = 0.25 mTorr (nom.), E_{CM} = 0 eV. Signals of hydrogen atom transfer / fragmentation (HAT+FRG, m/z 69), small signal of deuterium atom transfer / fragmentation (DAT+FRG, m/z 70), hydrogen atom transfer (HAT, m/z 147), double hydrogen atom transfer (2x HAT) are visible in the spectrum. Signals of HAT/DAT and 2x DAT channels are completely missing in this case. Deuterated variant of the HAT+FRG channel could originate from the H/D exchange, that would imply, that **$Im^{H+}SO_2N$** is reactive only towards C-H (*BDE* = 96 kcal·mol^{-1}) bond, leaving the O-H (*BDE* = 105 kcal·mol^{-1}) bond initially intact.

2.3.3.7 *Reactivity with tetrahydrofuran*

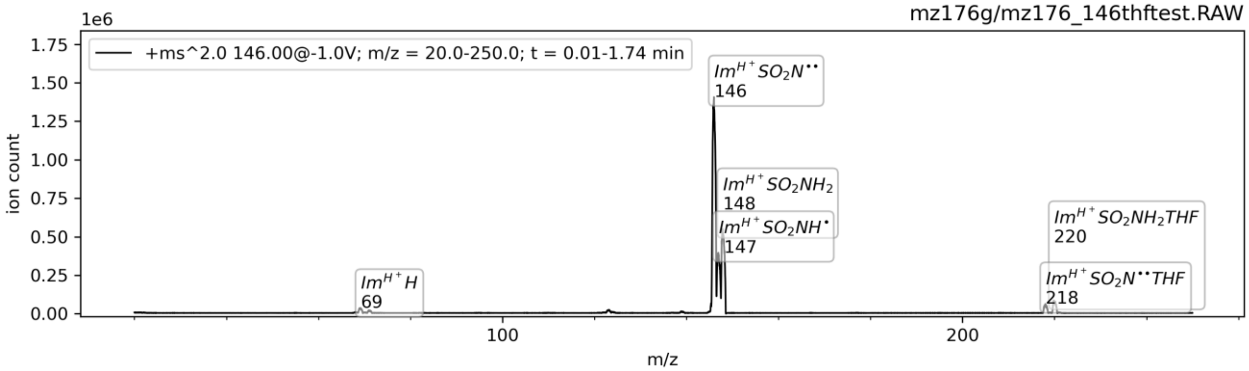

Figure S49. ESI-MS/MS spectrum of the gas-phase reaction of **$Im^{H+}SO_2N^{••}$** (m/z 146) parent ion with tetrahydrofuran (THF). Conditions: source conditions were similar to the one used in the Figure S28, *p*(tetrahydrofuran) = 0.08 mTorr (nom.), E_{CM} = 0 eV. Signals of hydrogen atom transfer with subsequent fragmentation (HAT+FRG, m/z 69), small signal of THF hydride abstraction (THF-H, m/z 71), hydrogen atom transfer (HAT, m/z 147), double hydrogen atom transfer (2x HAT) and adducts formation ($Im^{H+}SO_2N^{••}THF$, $Im^{H+}SO_2NH_2THF$) are visible in the spectrum.

2.3.3.8 *Reactivity with cyclohexane*

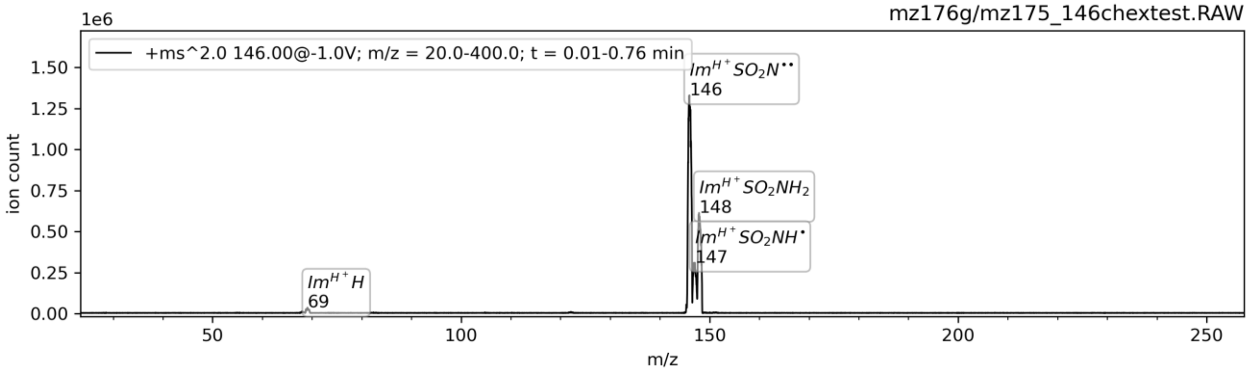

Figure S50. ESI-MS/MS spectrum of the gas-phase reaction of **$Im^{H+}SO_2N^{••}$** (m/z 146) parent ion with cyclohexane. Conditions: source conditions were similar to the one used in the Figure S28, *p*(cyclohexane) = 0.11 mTorr (nom.), E_{CM} = 0 eV. Signals of hydrogen atom transfer with subsequent fragmentation (HAT+FRG, m/z 69), hydrogen atom transfer (HAT, m/z 147), and double hydrogen atom transfer (2x HAT)) are visible in the spectrum. No adduct formation was observed.

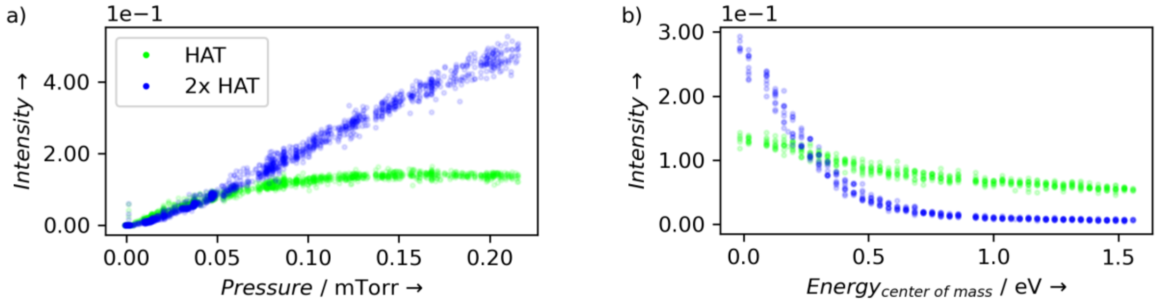

Figure S51. Pressure (a, E_{CM} = *0*) and collision-energy (b, *p*(cyclohexane) = 0.12 mTorr) dependencies of the hydrogen atom transfer (HAT, green) and double hydrogen atom transfer (2x HAT, blue) channels relative intensities in the gas-phase reactivity of (**$Im^{H+}SO_2N^{••}$**, m/z 146) parent ion with cyclohexane. Conditions: source conditions were similar to the one used in the Figure S28. The intensities of the channels have been plotted relatively to sum of selected ions intensities – m/z 146, m/z 147 and m/z 148 in this case. Other channels were neglected. Background was subtracted. The energy and the pressure dependencies suggest that the 2x HAT channel is a multiple collision reactivity channel. This is in agreement with the experiment described in the Figure S52.

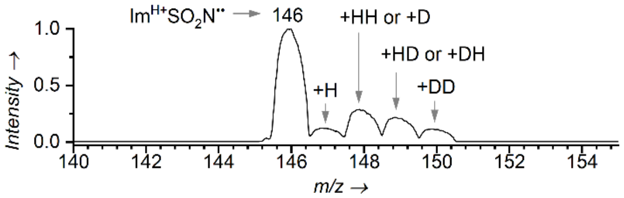

Figure S52. ESI-MS/MS spectrum of the gas-phase reaction of **$Im^{H+}SO_2N^{••}$** parent ion with 1:1 mixture of cyclohexane and cyclohexane-*d12*. Conditions: source conditions were similar to the one used in the Figure S28, E_{CM} = 0 eV, *p*(cyclohexane-*d0*,cyclohexane-*d12*) = 0.14 mTorr.

2.3.3.9 *Reactivity with ethane*

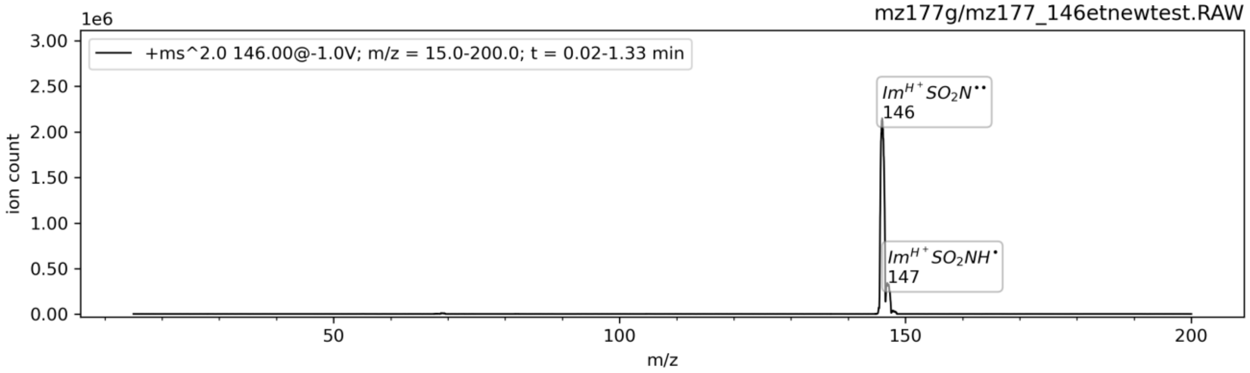

Figure S53. ESI-MS/MS spectrum of the gas-phase reaction of **$Im^{H+}SO_2N^{••}$** (m/z 146) parent ion with ethane. Conditions: source conditions were similar to the one used in the Figure S28, *p*(ethane) = 0.27 mTorr (nom.), E_{CM} = 0 eV. Signals of hydrogen atom transfer (HAT, m/z 147) and double hydrogen atom transfer (2x HAT)) are visible in the spectrum. No adduct formation was observed.

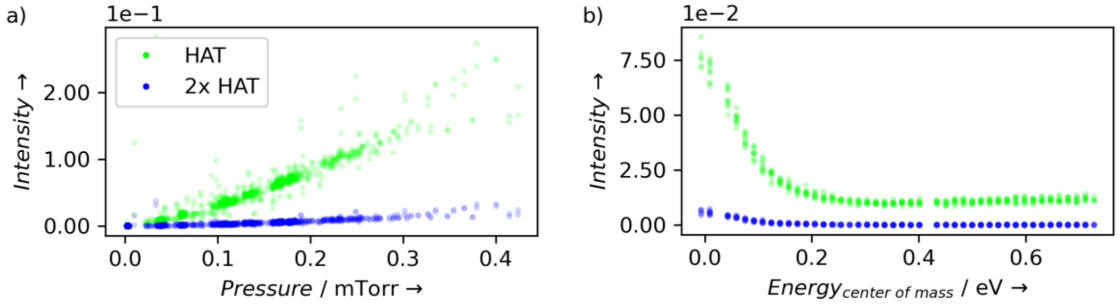

Figure S54. Pressure (a, E_{CM} = 0) and collision-energy (b, *p*(ethane) = 0.18 mTorr) dependencies of the hydrogen atom transfer (HAT, green), double hydrogen atom transfer (2x HAT, blue) channels relative intensities in the gas-phase reactivity of (**$Im^{H+}SO_2N^{••}$**, m/z 146) parent ion with ethane. Conditions: source conditions were similar to the one used in the Figure S28. The intensities of the channels have been plotted relatively to a sum of the selected ions intensities – m/z 146, m/z 147 and m/z 148 in this case. Background was subtracted.

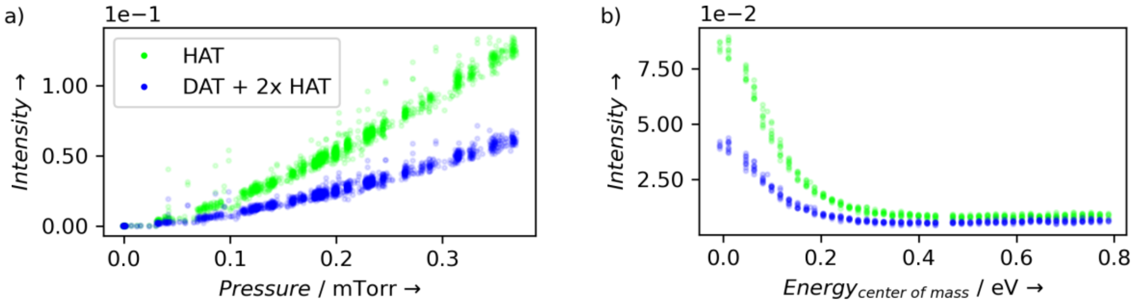

Figure S55. Pressure (a, E_{CM} = 0) and collision-energy (b, *p*(ethane-d$_3$) = 0.27 mTorr) dependencies of the hydrogen atom transfer (HAT, green), deuterium atom transfer and double hydrogen atom transfer (DAT + 2x HAT, blue) channels relative intensities in the gas-phase reactivity of (**$Im^{H+}SO_2N^{••}$**, m/z 146) parent ion with ethane. Conditions: source conditions were similar to the one used in the Figure S28. The intensities of the channels have been plotted relatively to a sum of the selected ions intensities – m/z 146, m/z 147 and m/z 148 in this case. Background was subtracted.

2.3.3.10 Reactivity with benzene

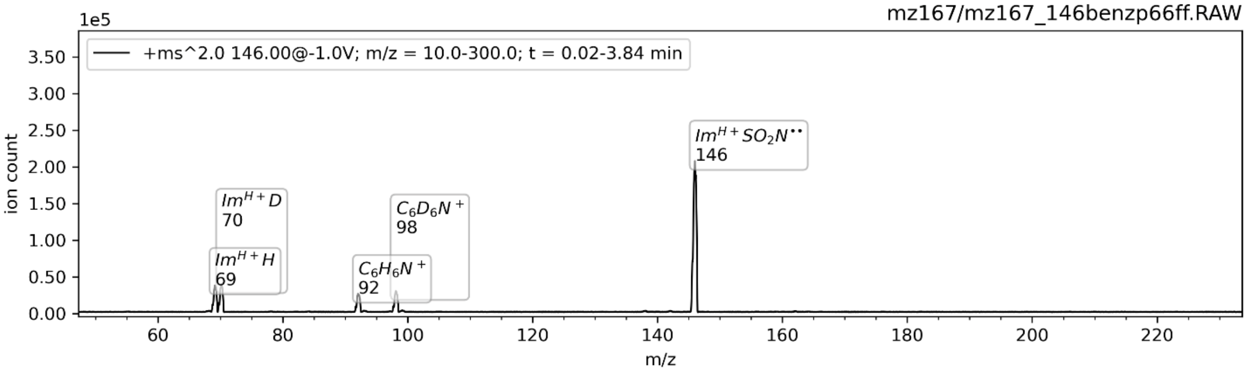

Figure S56. ESI-MS/MS spectrum of the gas-phase reaction of **$Im^{H+}SO_2N^{\bullet\bullet}$** (m/z 146) parent ion with with 1:1 mixture of benzene and benzene-*d6*. Conditions: source conditions were similar to the one used in the Figure S28, *p*(benzene-d0, benzene-d6) = 0.74 mTorr (nom.), E_{CM} = 0 eV. Signals of bond insertion or adduct formation with subsequent fragmentation (m/z 92 and m/z 98 for benzene fragments and m/z 69 and m/z 70 for imidazole fragments) are clearly visible from the spectrum. These fragments correspond either to a hydrogen atom transfer, subsequent rebound mechanism and fragmentation of the rebound product or to insertion of the nitrene radical into the C-H bond with subsequent fragmentation of the newly formed adduct.

2.3.4 Gas-phase reactivity of $Im^{H+}SO_2NH^{\bullet}$

The imidazole-1-sulfonyl amidyl radical (**$Im^{H+}SO_2NH^{\bullet}$**, m/z 147) reacted with: ethanethiol, acetylacetone, acetone, methanol, tetrahydrofuran, cyclohexane and ethane. The reactivity was dominated by hydrogen atom transfer. The nature of the hydrogen atom transfer (ECPT, PCET, concerted HAT) cannot be derived from our experiments.

2.3.4.1 Reactivity with ethanethiol

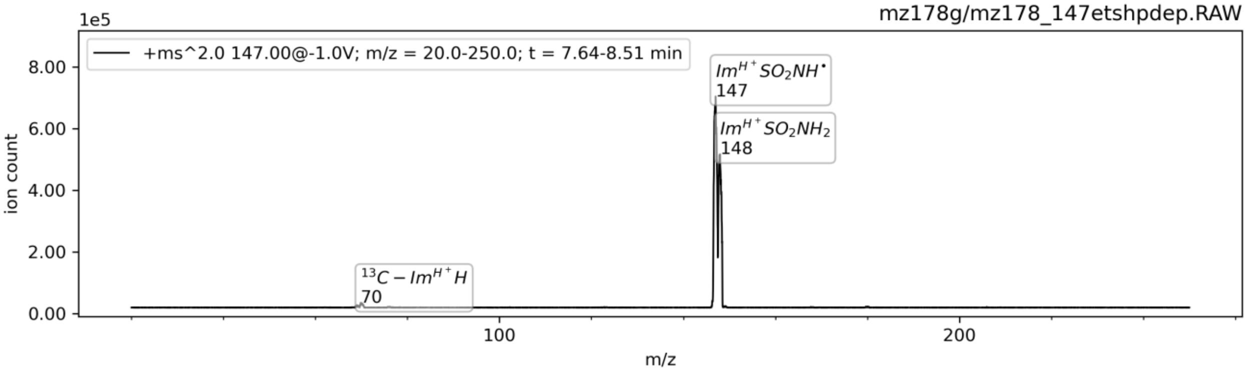

Figure S57. ESI-MS/MS spectrum of the gas-phase reaction of **$Im^{H+}SO_2NH^{\bullet}$** (m/z 147) parent ion with ethanethiol (C_2H_5SH). Conditions: source conditions were similar to the one used in the Figure S29, *p*(ethanethiol) = 0.07 mTorr (nom.), E_{CM} = 0 eV.

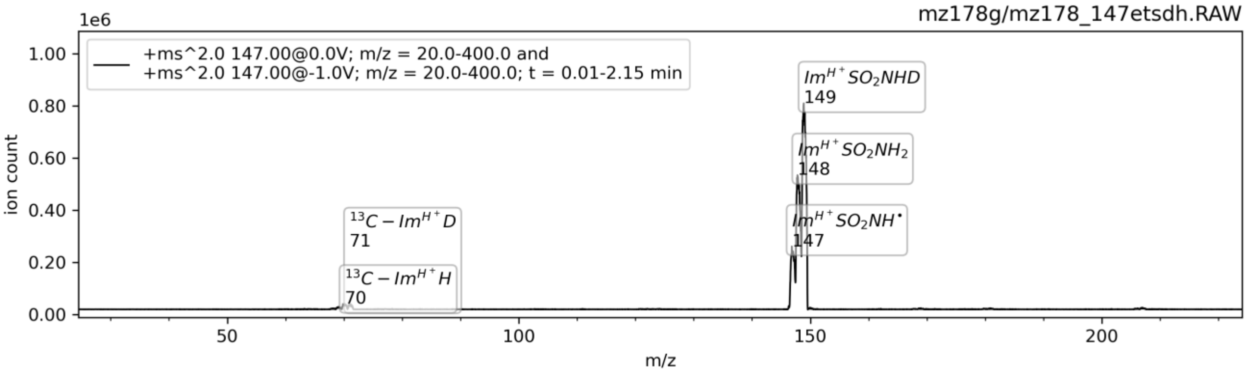

Figure S58. ESI-MS/MS spectrum of the gas-phase reaction of **$Im^{H+}SO_2NH^•$** (m/z 147) parent ion with ethanethiol (C_2H_5SH/ C_2H_5SD). Conditions: source conditions were similar to the one used in the Figure S29, *p*(C_2H_5SH/ C_2H_5SD) = 0.27 mTorr (nom.), E_{CM} = 0 eV. The collision gas were vapors from a tube containing ethanethiol/D2O emulsion, thus ratio of C_2H_5SH : C_2H_5SD is unknown.

2.3.4.2 Reactivity with acetylacetone

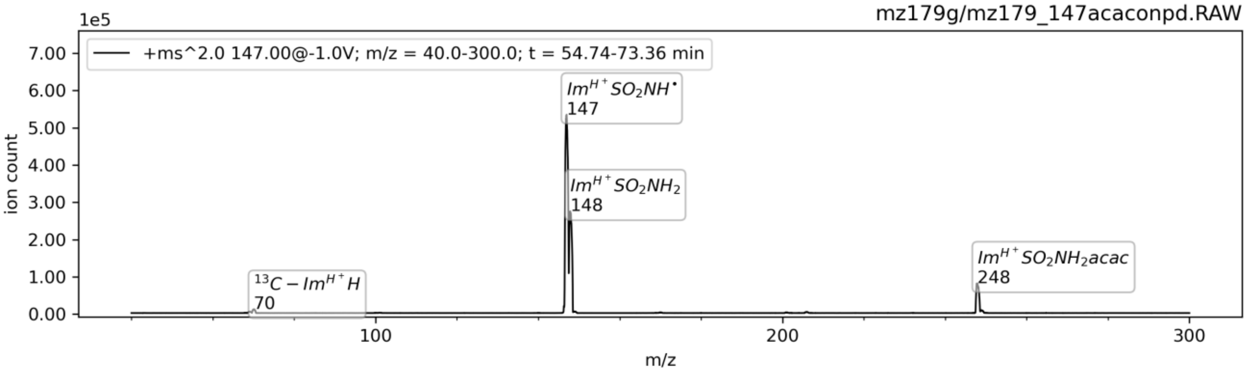

Figure S59. ESI-MS/MS spectrum of the gas-phase reaction of **$Im^{H+}SO_2NH^•$** (m/z 147) parent ion with acetylacetone (acac). Conditions: source conditions were similar to the one used in the Figure S29, *p*(acetylacetone) = 0.04 mTorr (nom.), E_{CM} = 0 eV.

2.3.4.3 Reactivity with 1,4-cyclohexadiene

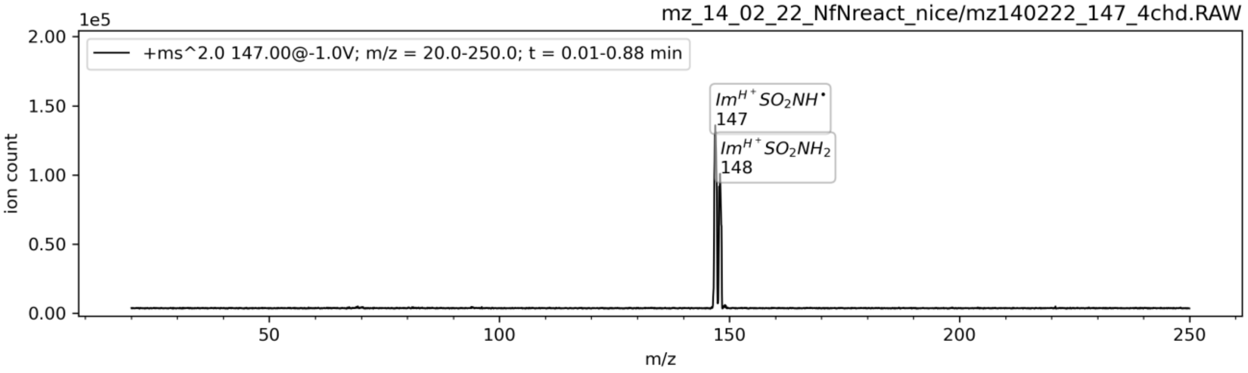

Figure S60. ESI-MS/MS spectrum of the gas-phase reaction of **$Im^{H+}SO_2NH^•$** (m/z 147) parent ion with 1,4-cyclohexadiene. Conditions: source conditions were similar to the one used in the Figure S29, *p*(1,4-cyclohexadiene) = 0.08 mTorr (nom.), E_{CM} = 0 eV.

2.3.4.4 *Reactivity with 1,3-cyclohexadiene*

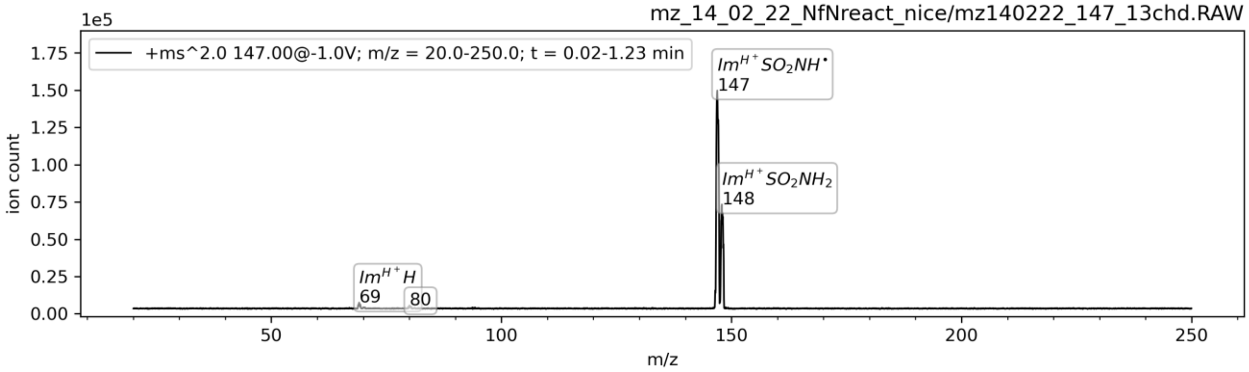

Figure S61. ESI-MS/MS spectrum of the gas-phase reaction of **$Im^{H+}SO_2NH^{\bullet}$** (m/z 147) parent ion with 1,3-cyclohexadiene. Conditions: source conditions were similar to the one used in the Figure S29, *p*(1,3-cyclohexadiene) = 0.07 mTorr (nom.), E_{CM} = 0 eV.

2.3.4.5 *Reactivity with acetone*

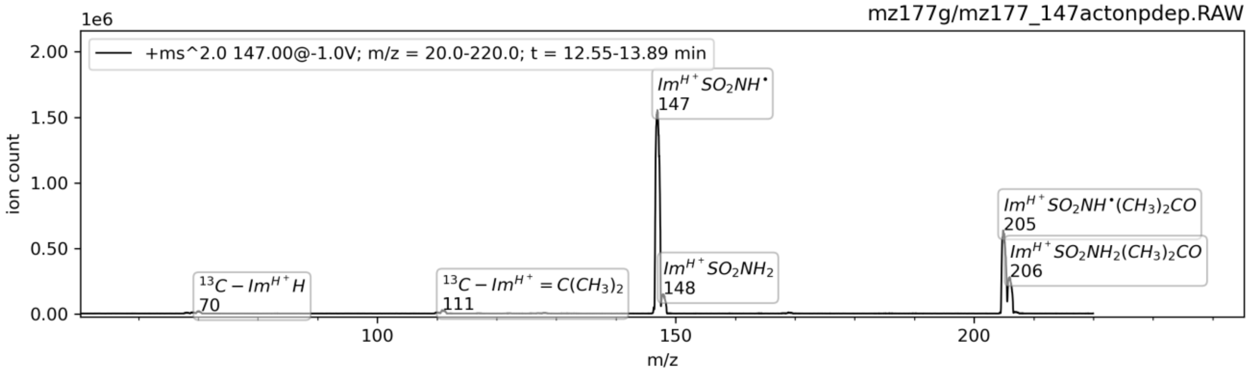

Figure S62. ESI-MS/MS spectrum of the gas-phase reaction of **$Im^{H+}SO_2NH^{\bullet}$** (m/z 147) parent ion with acetone. Conditions: source conditions were similar to the one used in the Figure S29, *p*(acetone) = 0.15 mTorr (nom.), E_{CM} = 0 eV.

2.3.4.6 *Reactivity with methanol*

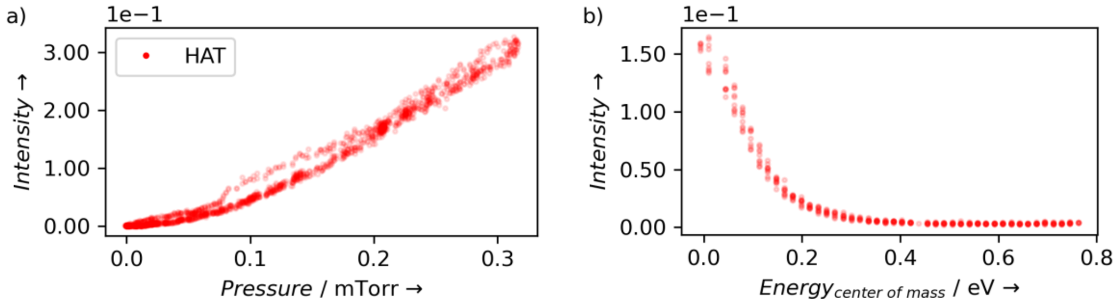

Figure S63. Pressure (a, E_{CM} = 0) and collision-energy (b, *p*(methanol) = 0.18 mTorr) dependencies of the hydrogen atom transfer (HAT, red) channel relative intensity in the gas-phase reactivity of **$Im^{H+}SO_2NH^{\bullet}$** (m/z 147) parent ion with methanol. Conditions: source conditions were similar to the one used in the Figure S29. The intensities of the channel have been plotted relatively to a sum of the selected ions intensities: m/z 147, m/z 148 in this case. Background was subtracted, other channels were neglected.

2.3.4.7 *Reactivity with tetrahydrofuran*

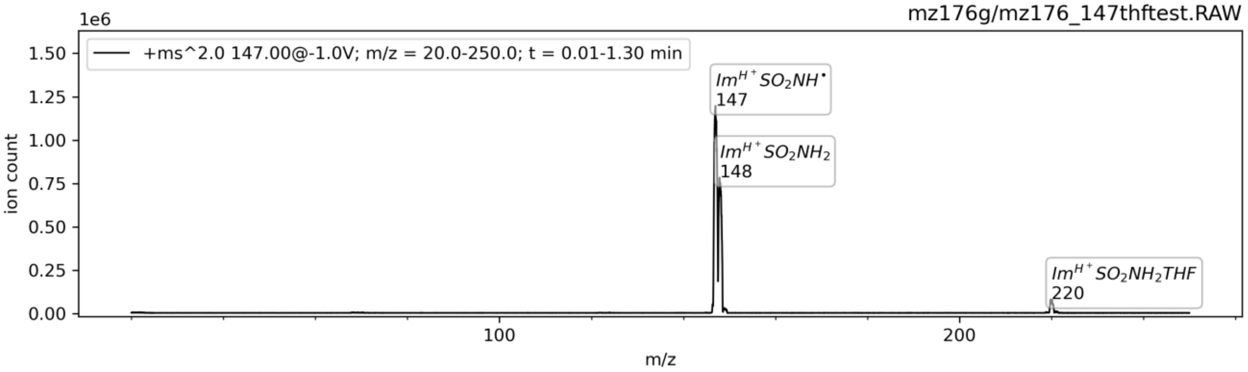

Figure S64. ESI-MS/MS spectrum of the gas-phase reaction of **$Im^{H+}SO_2NH^{•}$** (m/z 147) parent ion with tetrahydrofuran (THF). Conditions: source conditions were similar to the one used in the Figure S29, *p*(tetrahydrofuran) = 0.08 mTorr (nom.), E_{CM} = 0 eV. Signals of hydrogen atom transfer (HAT, m/z 148) and hydrogen atom transfer with subsequent adduct formation ($Im^{H+}SO_2NH_2$(THF)) are visible in the spectrum.

2.3.4.8 *Reactivity with cyclohexane*

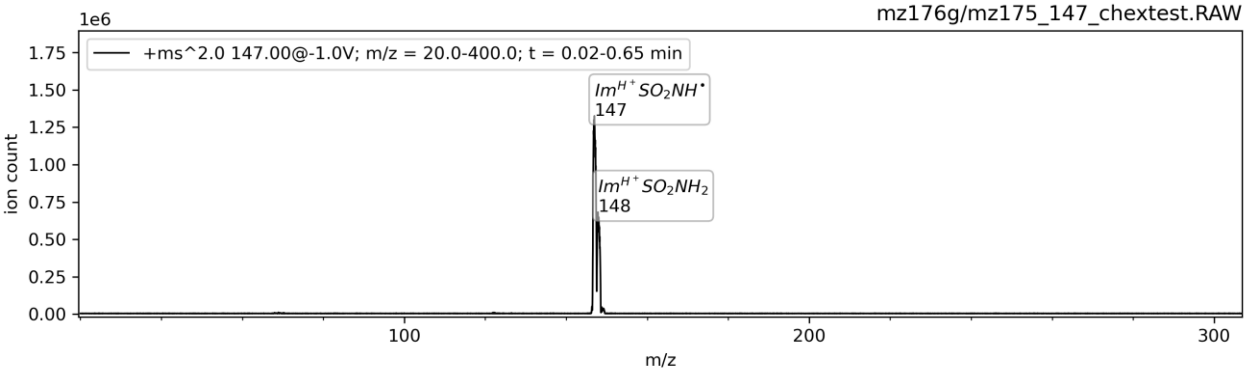

Figure S65. ESI-MS/MS spectrum of the gas-phase reaction of **$Im^{H+}SO_2NH^{•}$** (m/z 147) parent ion with cyclohexane. Conditions: source conditions were similar to the one used in the Figure S29, *p*(cyclohexane) = 0.12 mTorr (nom.), E_{CM} = 0 eV. Only intense signal visible in the spectrum is the signal of a hydrogen atom transfer (HAT, m/z 148). The faint signal of m/z 149 probably corresponds to ^{13}C-**$Im^{H+}SO_2NH_2$** which stems from ^{13}C-**$Im^{H+}SO_2N^{••}$** isotopic impurity of the parent ion.

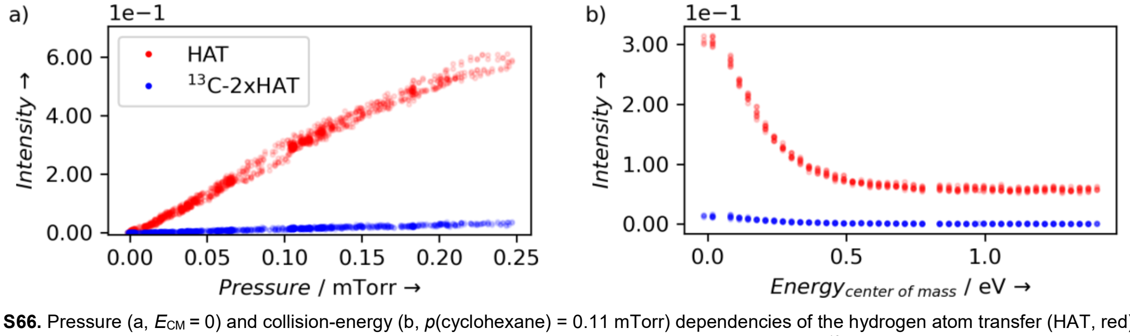

Figure S66. Pressure (a, E_{CM} = 0) and collision-energy (b, *p*(cyclohexane) = 0.11 mTorr) dependencies of the hydrogen atom transfer (HAT, red) channel relative intensity in the gas-phase reactivity of **$Im^{H+}SO_2NH^{•}$** (m/z 147) parent ion with cyclohexane (^{13}C-**$Im^{H+}SO_2N^{••}$** 2x HAT channel intensity is plotted too). Conditions: source conditions were similar to the one used in the Figure S29. The intensities of the channels have been plotted relatively to a sum of the selected ions intensities: m/z 147, m/z 148 and m/z 149 in this case. Background was subtracted, other channels were neglected.

2.3.4.9 *Reactivity with ethane*

Based on Q1 spectrum, we believe that the m/z 147 channel consisted from 94% of **$Im^{H+}SO_2NH^{\bullet}$** and 6% of ^{13}C-**$Im^{H+}SO_2N^{\bullet\bullet}$**.

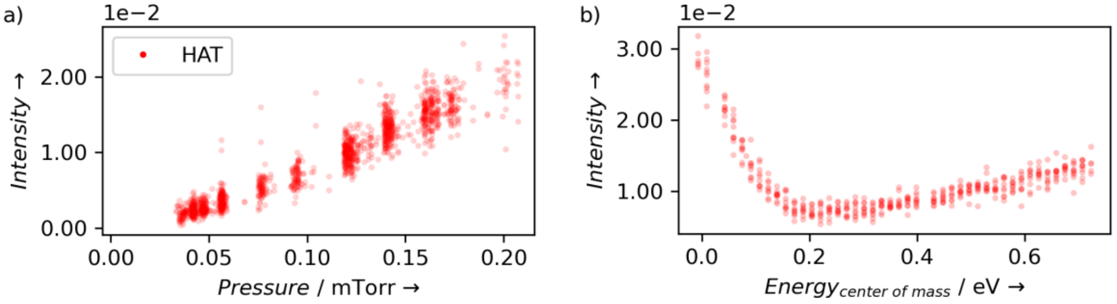

Figure S67. Pressure (a, E_{CM} = 0) and collision-energy (b, *p*(ethane) = 0.25 mTorr) dependencies of the hydrogen atom transfer (HAT, red) channel relative intensity in the gas-phase reactivity of **$Im^{H+}SO_2NH^{\bullet}$** (m/z 147). Conditions: source conditions were similar to the one used in the Figure S29. The intensities of the channels have been plotted relatively to a sum of the selected ions intensities: m/z 147 and m/z 148 in this case. Background was subtracted, other channels were neglected. The slope of the pressure dependence curve is approximately three times bigger then what would correspond to the reactivity of the ^{13}C-**$Im^{H+}SO_2N^{\bullet\bullet}$** contaminant. The energy dependence differs from the one observed for **$Im^{H+}SO_2N^{\bullet\bullet}$**.

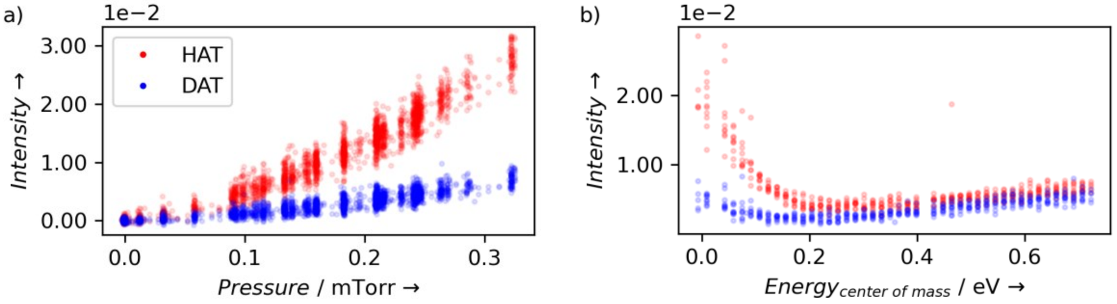

Figure S68. Pressure (a, E_{CM} = 0) and collision-energy (b, *p*(ethane-d3) = 0.25 mTorr) dependencies of the hydrogen atom transfer (HAT, red) channel relative intensity in the gas-phase reactivity of **$Im^{H+}SO_2NH^{\bullet}$** (m/z 147) parent ion. Conditions: source conditions were similar to the one used in the Figure S29. The intensities of the channels have been plotted relatively to a sum of the selected ions intensities: m/z 147 and m/z 148 and m/z 149 in this case. Background was subtracted, other channels were neglected. The energy dependence and slope of pressure dependence differ from the one observed for **$Im^{H+}SO_2N^{\bullet\bullet}$**. In this case, the contamination with ^{13}C-**$Im^{H+}SO_2N^{\bullet\bullet}$** was between 3-5% of the overall channel intensity.

3 IRPD spectra, DFT calculations (S69-S76)

3.1 Experimental Details

Ion spectroscopy (IRPD) technique has been described elsewhere in detail.[6,7] Samples and conditions were similar as the one described in the mass spectrometry section. Special attention has been paid to the voltage of the transfer quadrupole between the ion source and first mass selection quadrupole. This voltage was kept at a lower offset voltage relative to the preceding lens (the ions were thus accelerated) to ensure that the ions do not reside for a prolonged period of time in this transfer quadrupole. The transfer quadrupole is located in a high-pressure region, close to the entrance. If the ions would accumulate there, it may lead to their rearrangement, or fragmentation. The general procedure of IRPD experiments is described in the Figure S69. The number of helium pulses and the ion trap filling time were always optimized for each experiment in order to achieve the best signal to noise ratio. If not stated otherwise, the cycle time (N) has been set to 1 second.

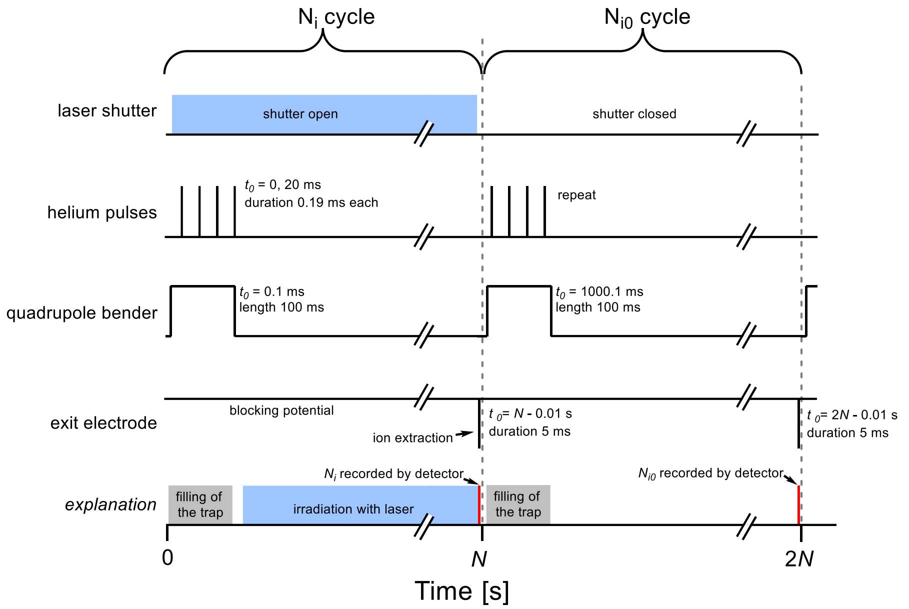

Figure S69. Pulse sequence of the IRPD experiments. General Experimental procedure: Ions were mass selected by the first quadrupole and guided by a bender and an octopole to the wire quadrupole ion trap. There they were cooled to 3K with helium buffer gas. At this temperature and pressure, the ions formed clusters with helium. These clusters were then irradiated at various wavelengths by IR laser. Usually the energy of a single absorbed photon is enough to dissociate the helium from the ion and thus to report the photon absorption at a specific wavelength. We measured the whole spectrum by varying the wavelength of the irradiation, reporting the dissociation yield ($1-N_i/N_{i0}$) as a function of absorption coefficient at specific wavelength. The N_{i0} cycle serves as a reference value for the number of clusters formed.

3.2 Computational Details

The calculations were performed with the B3LYP DFT functional[8] on the 6-311+G** basis set in Gaussian G16. D3 version of Grimme´s dispersion with Becke-Johnson damping was used.[9] For spectra presented in the main article in Figures 4, 5 and SI spectra in Figures S70, S71, S72, sulfur atom of the SO_2 group was described with a PC-3 basis set for a better prediction of the electron dispersion.[10] Structures pre-optimizations were carried out with AM1[11] and further refined with the B97 DFT functional[12] on the 6-31+G basis set. Except anharmonic calculation in Figure S70d, the IR vibrational spectra were scaled by factor 0.99 in the range below 2500 cm^{-1} and by factor 0.965 in the upper range. Relative energies (E_{rel} *(0K)*) in Figures S71 and S72 are electronic energies with zero-point vibrational energy correction calculated at the same level of theory as the vibrational spectra.

3.3 Benchmark of computational methods used to predict vibrational spectra

We benchmarked various calculation methods by comparing spectra predicted by them with the experimental spectrum of **$Im^{H+}SO_2N_3$** (Figure S70). When comparing spectra (b) (with PC3 on sulfur atom) and (c) (without PC3 on sulfur atom), it is apparent that the smaller 6-311+G** basis set predicts both SO_2 symmetric and SO_2 asymmetric bands wrong by more than 50 cm^{-1}. The anharmonic calculation spectrum (d) has slightly worse agreement than the PC-3/6-311+G** calculated spectrum (b). Moreover, the computational costs are substantially higher for the anharmonic calculations than for the harmonic one. Our benchmark shows clearly that the use of harmonic vibrational spectra calculated with the PC-3 basis set on the sulfur atom is the best choice for our system.

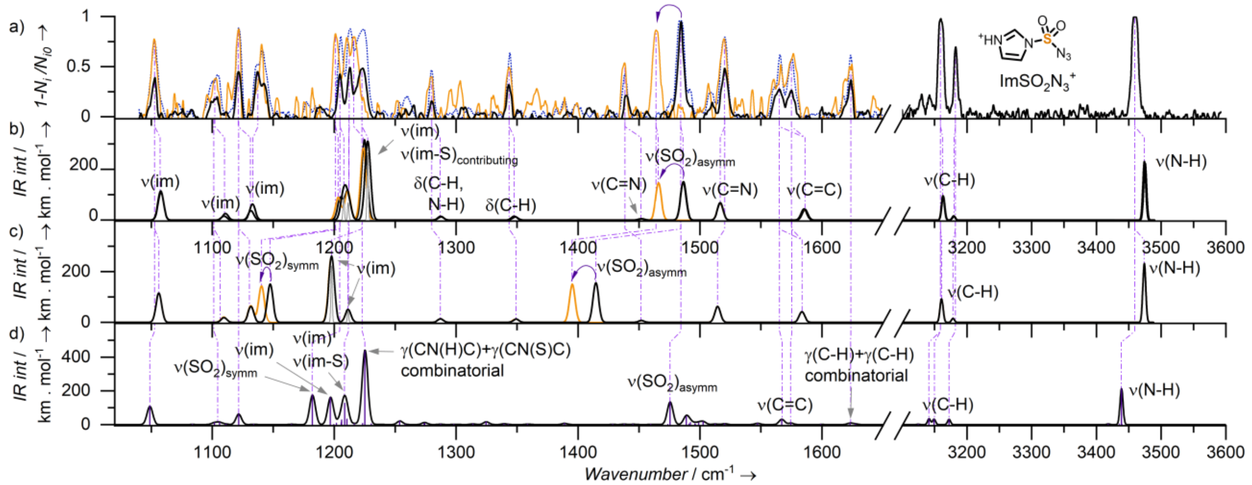

Figure S70. Comparison between IRPD (a) and calculated (b,c,d) vibrational spectra of $\mathbf{Im^{H+}SO_2N_3^+}$. (b) Vibrational spectrum calculated with B3LYP/6-311+G** basis set and PC3 basis set on sulfur atom. (c) same as (b) with 6-311+G** basis set on sulfur atom. (d) Anharmonic calculation (no scaling), PC-3 basis set used to describe the sulfur atom. Gray lines in (b) and (c) represents calculated spectrum with a narrower band distribution in the 1200-1250 cm^{-1} region. Two black lines in calculated spectrum (b) represent two conformers which differ by 1 kJ/mol in the energy and are separated by a 5 kJ/mol barrier. Full purple lines in the spectrum (d) represent vibrations and their intensity. The assignment of the experimental band which corresponds to the combinatorial one is speculative.

When assigning the structure of the $\mathbf{Im^{H+}SO_2N^{\bullet\bullet}}$ ion, we calculated theoretical spectrum of the pseudo-Curtius rearranged $\mathbf{Im^{H+}NSO_2}$ (Figure S71). The calculated spectrum of $\mathbf{Im^{H+}NSO_2}$ does not reproduce well the bands detected below 1250 cm^{-1} (Figure S72). The intense imidazole C-H stretch vibration band (1213 cm^{-1}) is completely omitted in case of the $\mathbf{Im^{H+}NSO_2^+}$ theoretical spectrum and the position of the symmetric SO_2 band vibration of $\mathbf{Im^{H+}NSO_2^+}$ (Figure S71) is substantially blue-shifted compared to the experimental spectrum (Figure S71). Hence, we can exclude this rearrangement happening under our experimental conditions. We can also exclude the singlet nitrene based on the position of the symmetric SO_2 band vibration (Figure S71d).

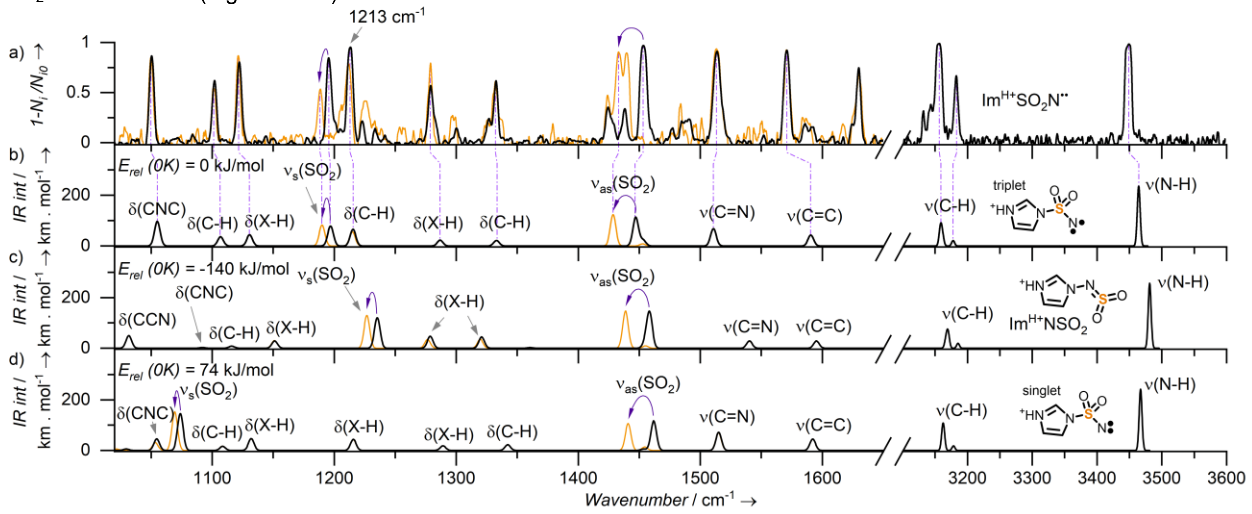

Figure S71. Comparison between IRPD (a) and calculated (b,c,d) vibrational spectra of $\mathbf{Im^{H+}SO_2N^{\bullet\bullet}}$ (respectively $\mathbf{Im^{H+}NSO_2}$). Vibrational spectra were calculated with B3LYP/6-311+G** basis set and PC3 basis set on sulfur atom. (b) $\mathbf{Im^{H+}SO_2N^{\bullet\bullet}}$ triplet spin state, (c) pseudo-Curtious rearranged ion $\mathbf{Im^{H+}NSO_2}$ (d) $\mathbf{Im^{H+}SO_2N^{\bullet\bullet}}$ singlet spin state. The singlet is energetically disfavored by +74 kJ/mol relative to the triplet state when electronic energies with zero-point energies are compared. Moreover, the symmetric band of SO_2 vibration which is easily identified by its isotope shift is on a wrong position for the singlet when compared to the experimental spectrum. This band fits perfectly in the triplet spin state calculation.

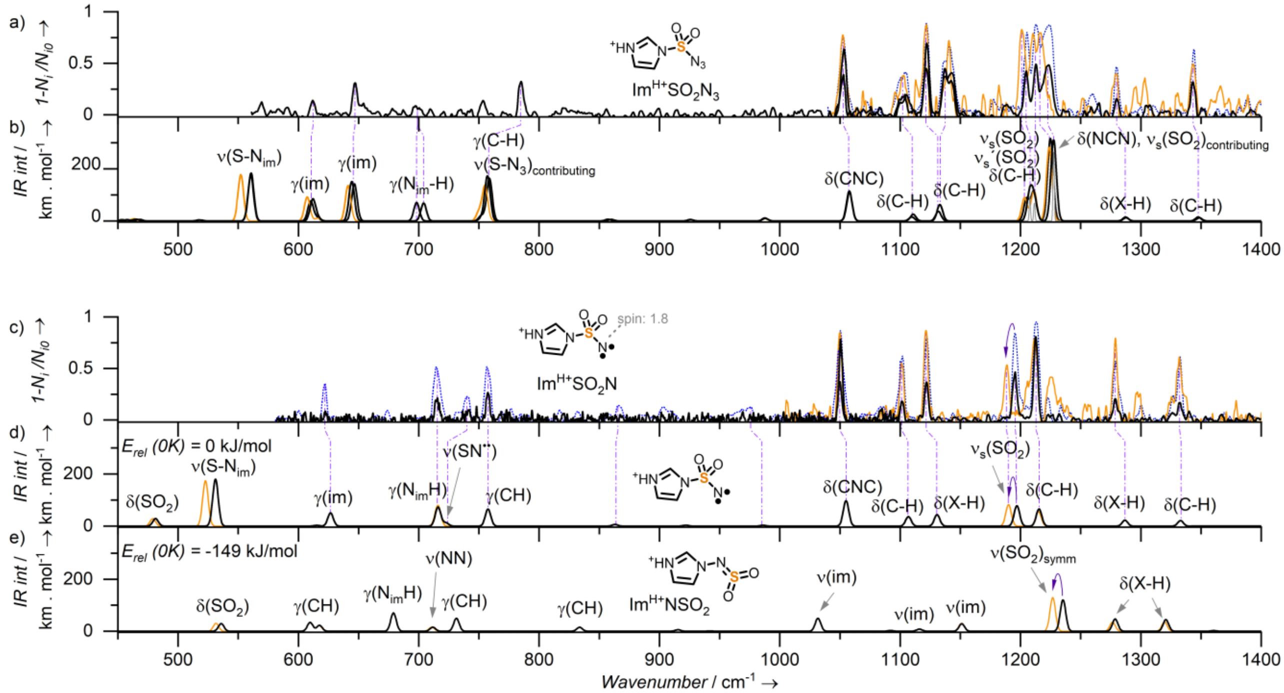

Figure S72. Comparison between IRPD and calculated (B3LYP/6-311+G**, gd3-bj, PC-3 basis set used for sulfur atom[13]) vibrational spectra in 400-1400 cm^{-1} range. (a) IRPD and (b) calculated vibrational spectra of **Im^{H+}SO$_2$N$_3$**. (c) IRPD (blue dotted line is spectrum of 4 second cycles scan) and (d,e) calculated vibrational spectra of **Im^{H+}SO$_2$N**. In case of **Im^{H+}SO$_2$N**, the low signal of ^{34}S labelled compound made recording of spectrum with reasonable signal-to-noise ratio in range below 1000 cm^{-1} impossible. The band in spectrum (c) assigned in spectrum (d) as (νSN) could theoretically originate also from minor contamination with **Im^{H+}NSO$_2$** (thus being γCH from spectrum (e)).

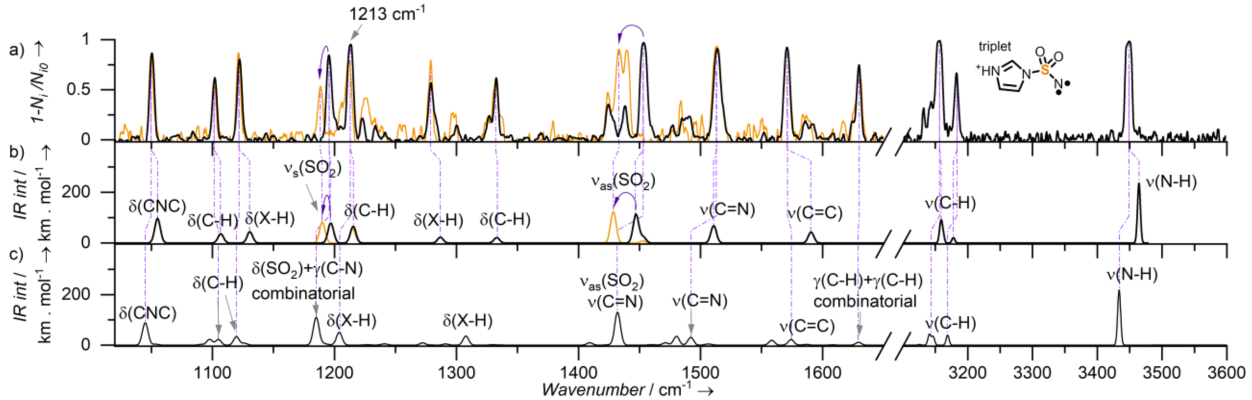

Figure S73. Comparison between IRPD (a) and calculated (b, c) vibrational spectra of **Im^{H+}SO$_2$N$^{\bullet\bullet}$**. (b) Vibrational spectrum calculated with B3LYP/6-311+G** basis set and PC3 basis set on sulfur atom from Figure S71, (c) same as (b) using anharmonic calculation (no scaling). The assignment of the experimental band which corresponds to the combinatorial ones is speculative. The overlap between anharmonic calculation and experimental spectrum is worse than the one of the scaled harmonic calculation. The combinatorial band at 1623 cm^{-1} in the spectrum c) originating from C-H vibrations of the imidazole ring is at the same position as in the case of **Im^{H+}SO$_2$N$_3$** (Figure S70, spectrum (d) 1623 cm^{-1}).

3.4 Geometry of optimized DFT structure of $ImH^{+}SO_2N_3$

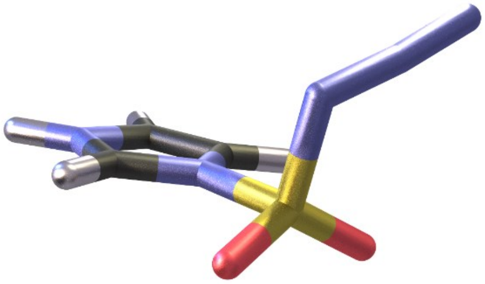

Figure S74. Calculated (B3LYP/6-311+G**, gd3-bj, PC-3 basis set used for sulfur atom[10]) structure of $\mathbf{Im^{H+}SO_2N_3}$.

3.5 Enthalpy of selected $NfN^{\bullet-}$ reactions

Reaction	HAT Reactivity with $\mathbf{CF_3SO_2N^{\bullet-}}$ kJ·mol^{-1}	HAT Reactivity with $\mathbf{CF_3NSO_2^{\bullet-}}$ kJ·mol^{-1}	PT Reactivity with $\mathbf{CF_3SO_2N^{\bullet-}}$ kJ·mol^{-1}
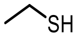	-55	-23	+151
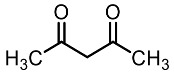	-10	+22	+133
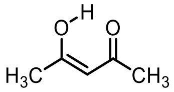	+9	+41	+114

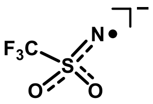

$\mathbf{CF_3SO_2N^{\bullet-}}$

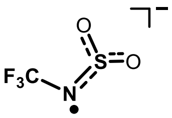

$\mathbf{CF_3NSO_2^{\bullet-}}$

Table 1. Calculated (B3LYP/6-311+G**) enthalpies of hydrogen atom transfer (HAT) and proton transfer (PT) reactions of $\mathbf{CF_3SO_2N^{\bullet-}}$ and $\mathbf{CF_3NSO_2^{\bullet-}}$ with ethanethiol, acetylacetone keto-form and enol-form. As apparent from the listed energies, proton transfer without subsequent electron transfer is energetically disfavored.

Due to anionic nature of the $NfN^{\bullet-}$, we believe that gas-phase reactivity with acetylacetone is initiated by proton abstraction either from C-H, or from the O-H bond, concerted with an electron transfer from the delocalized π-system. The spin density of the product lies mainly on the carbon atom. Mulliken spin densities summed into heavy atoms: +0.8 for the center carbon atom, +0.2 for each of the oxygen atoms and -0.2 for each of the carbonyl carbon atoms.

3.6 Enthalphy of possible $NfN^{\bullet-}$ fragmentation channels

The desired $NfN^{\bullet-}$ (m/z 297, $\mathbf{C_4F_9SO_2N^{\bullet-}}$) ion can theoretically undergo pseudo-Curtious rearrangement forming isobaric $\mathbf{C_4F_9NSO_2^{\bullet-}}$ ($\Delta H_{calc,CF3}$ = -147 kJ/mol). To test this option, we compared our theoretical calculations (Figure S75) with an experimental fragmentation spectrum of the m/z 297 parent ion (Figure S76). The main experimentally observed fragmentation is a loss of nonafluorobutyl radical ($C_4F_9^{\bullet}$), resulting in the detection of SO_2N^{-} (m/z 78) ion. This is in agreement with the thermodynamically lowest direct fragmentation pathway of $\mathbf{CF_3SO_2N^{\bullet-}}$ ($\Delta H_{calc,CF3}$ = 151 kJ/mol). In contrast, the pseudo-Curtious rearranged $\mathbf{C_4F_9NSO_2^{\bullet-}}$ ion would favorably most probably fragment through a neutral loss of SO_2 forming $\mathbf{C_4F_9N^{\bullet-}}$ ($\Delta H_{calc,CF3}$ = 202 kJ/mol). As this fragmentation channel is absent, we conclude that the ions observed and characterized by us are indeed the one of the nitrene radical anion ($\mathbf{C_4F_9SO_2N^{\bullet-}}$).

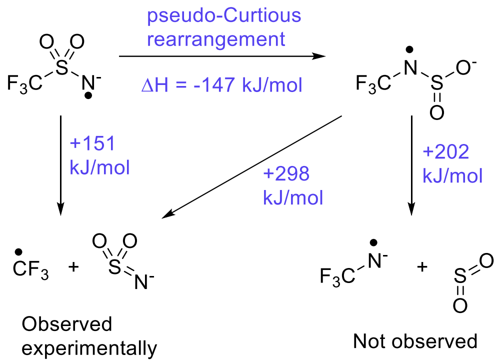

Figure S75. Comparison of most-favored direct fragmentation pathways of **$CF_3SO_2N^{\bullet-}$** and for **$CF_3NSO_2^{\bullet-}$**.

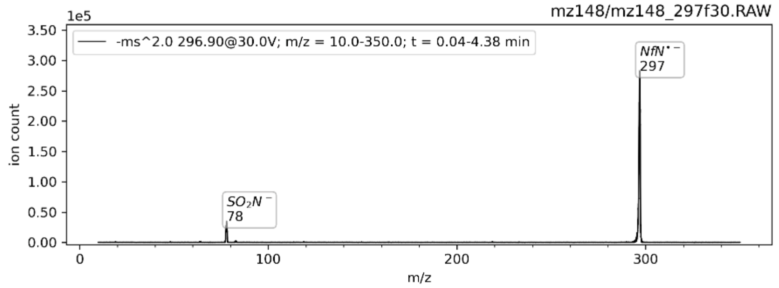

Figure S76. ESI-MS/MS fragmentation spectrum of the m/z 297 parent ion. Conditions: electron multiplier voltage 1800 V, sheath gas pressure 20 psi, auxiliary gas not used, spray voltage 5.0 kV, capillary temperature 275 °C, capillary voltage 0 V, tube lens voltage -120 V, p(Xe) = 0.14 mTorr (nominal), E_{cm} = 8.8 eV.

4 Cyclic voltammetry

To find out if electron transfer between excited $Ru(bipy)_3(PF_6)_2$ and **NfN_3** is feasible, we measured redox potential of **NfN_3** by cyclic voltammetry. The reduction of **NfN_3** is irreversible, therefore we obtained just half-wave reduction potential (Figure S77). Comparison between experiment and redox potential of the excited $^*Ru(bipy)_3^{2+}$ suggests that $^*Ru(bipy)_3^{2+}$ may transfer an electron to the **NfN_3**, but the results are inconclusive as half-wave potential of **NfN_3** lies lower than redox potential of the excited $^*Ru(bipy)_3^{2+}$. Measurements were performed with Autolab PGSTAT204.

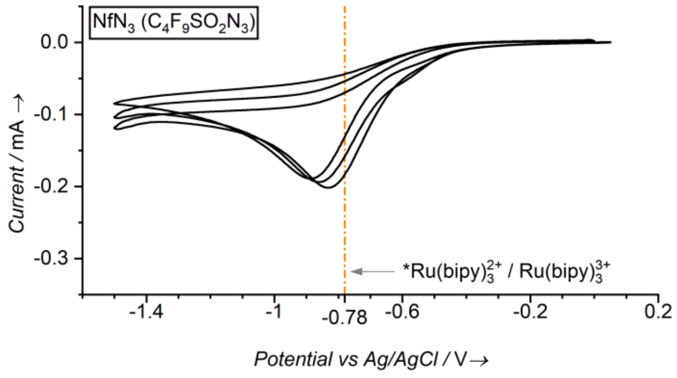

Figure S77. Voltammograms of the **NfN_3**. The gold dash-dotted vertical line lies at the redox potential of $^*Ru(bipy)_3^{2+}$ / $Ru(bipy)_3^{3+}$ pair (determined by excited-state quenching experiments).[14,15] Conditions: 0.1M tetrabutylammonium hexafluorophosphate ($TBAPF_6$) in acetonitrile. Reference electrode: Ag/AgCl in acetonitrile. Working electrode: Glassy carbon. Prior to the measurements, the redox potential of ferrocene was recorded (0.494 V). Before each cycle, glassy carbon electrode was polished and the whole system was purged by nitrogen. Freshly prepared 0.1 mM solutions of NfN_3 were used. Depicted scans have been recorded at 100 mV/s scan rate. The peak potential did not shift substantially (0.1 V) when other scan rates between 10 to 200 mV were used. The working electrode was polished prior to every measurement. Usage of non-polished electrode for this system renders measurements irreproducible.

5 XYZ coordinates of the calculated structures

Optimized Structures
The format of individual records is following:

number_of_atoms
Name
Charge Multiplicity
Electronic_energy(Hartree)
Zero_point_energy(Hartree)
Number_of_imaginary_freqs
Method/Basis_set
atom1 x y z
atom2 x y z
...

4
CF3_anion
-1 1 -337.7422581 0.0088408 0 RB3LYP-D3/6-311+G(D,P)
C 0.0393873979 -0.3675370791 -0.0553541249
F -0.0222767507 0.2046377665 1.2664007197
F 1.3600502694 0.0623446807 -0.4435540544
F -0.7508612412 0.5971425445 -0.7760495503

5
CF3N_radical_anion
-1 2 -392.49062 0.0134969 0 B3LYP-D3/6-311+G(D,P)
C 0.023560462 -0.3007285118 0.0444743883
F 0.0367645676 0.4377330086 1.3488872192
F 1.3437507595 -0.037070999 -0.3679571413
F -0.7940848531 0.577778277 -0.6927954187
N -0.330664318 -1.554806066 0.0112639357

8
CF3NSO2_conf2
-1 2 -941.304552 0.0261695 0 B3LYP-D3/6-311+G(D,P)
C -0.0579907783 -0.0703634242 0.0051739971
F -0.6831499784 -0.0244189366 1.2428371339
F 1.2908781281 -0.1925445409 0.331739762
N -0.5466520174 -0.9573481295 -0.8906758027
S -0.2572593645 -2.5225514965 -0.6987738075
O -0.9220933186 -3.2315023775 -1.7933392053
F -0.1787672436 1.2159945855 -0.4731090196
O -0.490751527 -3.0328622859 0.6584066955

8
CF3NSO2_conf3
-1 2 -941.3045521 0.0261711 0 B3LYP-D3/6-311+G(D,P)
C 0.0344371914 0.0213136987 0.077257062
F -0.821370496 0.0899469263 1.1746339118
F 1.2960719046 0.0773752143 0.6520216285
N -0.1893640972 -0.9800421842 -0.8031402213
S -0.3776161554 -2.4802294499 -0.2702705405
O -0.5781444924 -3.3410271065 -1.4372076973
F -0.1015606516 1.2455472989 -0.5390234834
O 0.6236848371 -2.9180836241 0.7108980352

8
CF3NSO2
-1 2 -941.3045521 0.0261713 0 B3LYP-D3/6-311+G(D,P)
C -0.0075312915 -0.0410616137 -0.0235984841
F 0.1713766051 0.0294630571 1.356213051
N 1.1086278826 -0.1908097433 -0.7719013802
S 2.1780232638 -1.3215503752 -0.3869984334
O 1.6055464059 -2.6448143334 -0.1078294951
F -0.6504627563 1.133765392 -0.3452581051
F -0.9915076333 -1.0084841453 -0.1691323575
O 3.2556927611 -1.2643811444 -1.3759368939

5
CF3N_tripplet
0 3 -392.3933364 0.0155266 0 B3LYP-D3/6-311+G(D,P)
C 0.0580955307 -0.1670078931 0.0683354155
F -0.0035579153 0.3269730301 1.3144594996
F 1.3104786322 -0.0201258411 -0.3907893724
F -0.7729100041 0.5379117457 -0.7146682869
N -0.3127796256 -1.5548453329 0.0665357273

4
CF3_radical
0 2 -337.6727208 0.0118575 0 B3LYP-D3/6-311+G(D,P)
C 0.1016593744 -0.1687017983 -0.0365883176
F -0.050919002 0.1187520712 1.2465829177
F 1.3442852243 0.0541873867 -0.4350396501
F -0.7687259212 0.492350253 -0.7835119598

8
CF3SO2N
-1 2 -941.2483947 0.0259648 0 B3LYP-D3/6-311+G(D,P)
C 0.0168073183 0.0291111293 -0.1151740066
S 0.0027083387 0.0046909802 1.7684100412
O 1.4295909212 -0.0141612196 2.0370911268
N -0.7282298803 -1.2613311522 2.2290107291
O -0.7270594365 1.230981445 2.0370911268
F -1.2373481163 0.0306753175 -0.6296009015
F 0.6454316437 1.1179203997 -0.6305605562
F 0.6452396624 -1.0562372433 -0.6296009015

3
SO2
0 1 -548.7325857 0.0070677 0 RB3LYP-D3/6-311+G(D,P)
S 0.1267507626 0. 0.0896263238
O 0.0053556468 0. 1.517986486
O 1.4329565996 0. -0.5009461431

4
SO2N_anion
-1 1 -603.5159634 0.0114179 0 RB3LYP-D3/6-311+G(D,P)
S 0.0831542827 0.1524135911 -0.0748016623
O -0.0670347225 -0.0026870579 1.3835920115
O 1.4910345886 -0.0481174354 -0.4638519593
N -1.0027717894 0.4594707515 -0.9983296433

4
SO2N_radical
0 2 -603.3843787 0.0111844 0 B3LYP-D3/6-311+G(D,P)
S 0.1491322343 0.1336529579 -0.0188231816
O -0.1334445854 0.0016284255 1.3741393311
O 1.4695889434 -0.0354595307 -0.534426412
N -0.9808942329 0.4612579966 -0.9742809909

3
SO2_radical_anion
-1 2 -548.7808648 0.0058054 0 B3LYP-D3/6-311+G(D,P)
S 0.0745186448 0. 0.052692639
O 0.0132662571 0. 1.5621997207
O 1.4772781072 0. -0.5082256931

4
SO2F_anion
-1 1 -648.7076234 0.0093239 0 RB3LYP-D3/6-311+G(D,P)
S 0.1421080254 -0.3488249312 0.0129201389
O -0.1303792695 0.2046643766 1.3431163224
O 1.4418518504 0.0582347825 -0.5307984358
F -0.9491982469 0.6470056215 -0.978629279

4
SO2F_radical
0 2 -648.5662327 0.0103259 0 B3LYP-D3/6-311+G(D,P)
S 0.1538080898 -0.195042874 -0.0015027808
O -0.1733404821 0.1428938321 1.3548371297
O 1.4393472421 0.0650059685 -0.5849637745
F -0.9154324904 0.5482229227 -0.921761828

4
CF2N_anion
-1 1 -292.6472587 0.0113168 0 RB3LYP-D3/6-311+G(D,P)
C 0.1241728522 0.1056237893 0.1717105502
N -0.0941551954 -0.0175987751 1.3363197392
F 1.3500965374 0.0037263149 -0.5819533404
F -0.7538145188 0.4048365836 -0.9346339587

4
CF2N_radical
0 2 -292.5455782 0.0129314 0 B3LYP-D3/6-311+G(D,P)
C 0.1455746717 0.1180595703 0.0583091625
N -0.0789704662 -0.0046761264 1.2828022444
F 1.3322007349 0.0003265251 -0.5011325536
F -0.7725052649 0.3828779436 -0.848535863

8
CF2NSO2F
-1 2 -941.287769 0.0264754 0 B3LYP-D3/6-311+G(D,P)
S 0.1184500486 0.0321742743 -0.1161120851
O 0.0831730063 -0.1161116577 1.3076462721
O 1.395858481 0.1080073837 -0.7677647295
N -0.9717445265 1.041249519 -0.577573034
C -1.0067454168 1.4738997313 -1.854727514
F -0.1373490088 2.493963963 -2.2134399446
F -2.2522731953 1.9170290313 -2.2326594514
F -0.4542069416 -1.4019508745 -0.6466519347

8
TfN_radical_anion
-1 2 -941.1635675 0.0252943 0 B3LYP-D3/6-311+G**
C 0.0045783751 0.0020277481 0.0191765516
F 0.13946516 -0.0211212717 1.3662511331
F 1.2496089958 0.1199770681 -0.4965418433
F -0.6558035479 1.1419387595 -0.288192728
S -0.8953421667 -1.5362683741 -0.665276942
O 0.0303833105 -2.6000605359 -0.2138129123
O -2.1963907913 -1.4055889098 0.0294480518
N -0.9990663231 -1.4148300501 -2.2110299502

9
TfNH_anion
-1 1 -941.829195 0.0373011 0 RB3LYP-D3/6-311+G(D,P)
N -0.3089700061 0.3185427274 0.1269050605
S -0.0877942693 0.16954238 1.6623854671
O 1.3173362437 0.1331029231 2.138778389
O -1.0469114961 0.9667538929 2.4453704542
C -0.6397472697 -1.6026240705 2.1122014619
F -1.9401385787 -1.8258767806 1.8201435871
F -0.4790321904 -1.8911114808 3.4245803837
F 0.0781736068 -2.5270199095 1.4200206105
H 0.5230110302 -0.0596955659 -0.3248936551

8
CurtTfN_radical_anion
-1 2 -941.2306342 0.0254275 0 B3LYP-D3/6-311+G**
C -0.0437077177 -0.1423553451 -0.069634291
F 0.1898262877 -0.06771417 1.3030547156
F 1.1881757979 0.1805775885 -0.6184317124
F -0.8288449179 0.9504940707 -0.3480785961
N -0.6384319596 -1.273945928 -0.5196467921
S -0.030991925 -2.7174092795 -0.0530520902
O -0.8802199841 -3.7697233791 -0.6859845308
O 1.4519593292 -2.8639854221 -0.2198933697

9
CurtTfNH_anion
-1 1 -941.8838536 0.0363985 0 RB3LYP-D3/6-311+G(D,P)
C -0.0116114048 -0.0414968333 -0.0496035148
N 0.0698911251 0.3692304351 1.2333418044
S 1.9014711643 0.1206107082 2.1935903455
O 2.4189177009 1.5129529758 2.2299121398
F 0.8919573406 0.5822436188 -0.8515007757
F 0.1993793292 -1.4138674717 -0.2979158042
F -1.2572499042 0.1701946062 -0.6408620706
O 1.3318084308 -0.3525817066 3.4934006971
H -0.5694603572 -0.1583991935 1.8201342995

9
EtSH
0 1 -478.0787776 0.0745926 0 RB3LYP-D3/6-311+G(D,P)
C 0.0229478499 0.0639781976 -0.0004777983
C -0.0329131534 -0.0030230362 1.5215744945
S 1.7591236484 -0.2316732545 -0.5570844692
H 0.6101528451 0.7554392028 1.9744970482
H -1.0555380681 0.170143792 1.8669690033
H 0.2860306996 -0.9824790566 1.8860905848
H -0.6221923679 -0.6965659929 -0.4422510589
H -0.297210988 1.0450702551 -0.3536775556
H 1.5272874104 -0.1208901074 -1.8806402487

8
EtS_anion
0 2 -477.4356534 0.0644859 0 B3LYP-D3/6-311+G(D,P)
C -0.0015982192 0.0021735428 -0.0000092061
C 0.0029379187 -0.0025110176 1.5247322744
S 1.6300662949 0.2134837298 2.2992616056
H 0.6206076657 -0.8035806434 -0.394794152
H -1.0197659099 -0.1332030978 -0.379140956
H 0.3857254248 0.9461602121 -0.3887168826
H -0.6360668161 0.7893111185 1.9331763919
H -0.4038010405 -0.9377976102 1.9271621393

15
pentadion
0 1 -345.9229782 0.1214771 0 RB3LYP-D3/6-311+G(D,P)
C 0.4672771801 -0.1357875682 -0.2889605128
C -0.0779886396 -0.2726097434 1.1374371999
H 1.5490466791 -0.2696892826 -0.3065965009
H -0.0302012126 -0.8757615454 -0.9161880831
C 0.1528592261 1.2868270118 -0.766278586
O 0.9337430948 2.1908149572 -0.5711518915
C -1.1869083928 1.4787436452 -1.4303304614
O -1.2114291058 -0.6500090338 1.3321230621
C 0.859970646 0.1372353096 2.2442470339
H -1.1897278925 0.9607110873 -2.3951111935
H -1.9728502898 1.021276088 -0.8222713963
H -1.385001725 2.5383200001 -1.5838200095
H 1.6878223331 -0.5774563682 2.2996138714
H 1.2983411911 1.1137381443 2.0184872275
H 0.3336251536 0.161238163 3.1970811229

15
pentadion_enol
0 1 -345.9309356 0.1223625 0 RB3LYP-D3/6-311+G(D,P)
C 0.0367926186 -0.0219213138 0.0116524415
C 0.0416139526 -0.091630637 1.5195992271
O 1.1159252209 -0.2447984125 2.1311139567
C -1.2146984455 0.0291353564 2.2185128976
C -1.2608672268 -0.0198438129 3.5863095919
C -2.5153212689 0.097503658 4.3868916023
O -0.1691512404 -0.1794398732 4.3204395672
H -2.1312478358 0.160264021 1.6607388578
H -0.6498802275 -0.7651125552 -0.4034007099
H -0.313305711 0.9623132998 -0.3145519155
H 1.042075488 -0.1938944522 -0.3694707744
H -3.3864814986 0.227086233 3.7460902779
H -2.6459733805 -0.7989874802 4.9993586281
H -2.4376852399 0.9483127134 5.0695187912
H 0.5954168894 -0.2446594656 3.6735198864

14
pentadion_anion
0 2 -345.2595504 0.1073223 0 B3LYP-D3/6-311+G(D,P)
C 0.1301484488 0.6049902251 0.1980871615
C 0.0162662196 0.2061314061 1.6066762364
H 0.9704285417 0.2120013257 -0.3686215099
C -0.8041240851 1.4943646574 -0.5034696049
O -1.5371741221 2.2626505012 0.0976871799
C -0.7756524022 1.4305768892 -2.0193304629
O -1.0409069376 0.2741550226 2.2122622664
C 1.2799538918 -0.3308871788 2.2527275221
H 0.2175421237 1.6996228381 -2.3928031823
H -0.9893469644 0.4146206701 -2.3651799693
H -1.5148576002 2.1188641636 -2.425932275
H 1.6392691881 -1.2131566919 1.713677577
H 2.0761971548 0.4190269779 2.2170314391
H 1.0741943122 -0.5962051648 3.2884685046

8
TfNd_eldist
-1 2 -941.2256936 0.0259215 0 B3LYP-D3/6-311+G(D,P)
N -0.2042810358 -0.188580093 0.0651898661
S 0.0039620614 0.0099938275 1.6022975883
O 1.3711073897 0.0061769182 2.1068984056
O -0.8557805046 0.968910155 2.284240313
C -0.6789488748 -1.6704318174 2.1495118914
F -1.9720592553 -1.8529379259 1.8068020653
F -0.6119601659 -1.7634988357 3.4979871121
F 0.0174915775 -2.7129914995 1.6485645171

15
ImN3
1 1 -939.0026188 0.0977332 0 RB3LYP-D3/6-311+G(D,P)
C 0.2503526865 0.2512800163 -0.0718396986
N 0.0662986577 0.0599236631 1.2892085751
C 1.2421168116 -0.1142845509 1.8896490319
N 2.1862164659 -0.0339674787 0.9415019336
C 1.588809567 0.1920648502 -0.2946335625
S 3.9211203933 -0.2100269681 1.2534212801
N 4.3264825006 1.341481549 1.6327317546
N 4.8346747694 2.0109600773 0.6915209025
N 5.3117538818 2.7075712922 -0.0403055494
O 4.4404549248 -0.6157139638 0.0019184102
O 3.9440343095 -0.9444408144 2.4554492861
H 1.4183216105 -0.2976224883 2.9371508799
H -0.8284377456 0.0368442234 1.7645860568
H -0.5730259328 0.3978128502 -0.7498368329
H 2.1650415175 0.2580004854 -1.2013931263

5
FSO2N

0 3 -703.2515144 0.0142031 0 B3LYP-D3/6-311+G(D,P)
S 0.1077777603 -0.0026924616 0.0762103852
O -0.0641397123 0.0182991744 1.4827390463
O 1.3765598751 0.0182991744 -0.5547178495
F -0.7234083551 1.1821091429 -0.5115269534
N -0.7887134493 -1.2609223422 -0.5577046284

13
ImN
1 3 -829.4118375 0.0859846 0 B3LYP-D3/6-311+G(D,P)
N 0.0477183756 0.1601185299 0.0392488125
C 0.0863544584 0.1495546439 1.3663711561
N 1.3634040522 -0.0885973198 1.7213969985
C 2.1484737113 -0.230638214 0.576728829
C 1.3146008505 -0.0737833332 -0.481417382
S 1.9211124686 -0.200680585 3.3829775126
O 3.0685189502 -1.0247055126 3.3295100678
O 0.7486218613 -0.4519214567 4.1360123468
N 2.3802621438 1.3985697953 3.5782994794
H -0.7391986896 0.2878333895 2.0463018835
H -0.7939708427 0.3067718092 -0.5074816972
H 1.5025425574 -0.1138393653 -1.5410050224
H 3.2027081095 -0.4426854098 0.6342485586

14
ImNH
1 2 -830.0786238 0.0974685 0 B3LYP-D3/6-311+G(D,P)
C -0.0035967808 -0.0457068339 -0.0152758233
N -0.0277280163 0.0324979483 1.323903909
C 1.2743413937 0.0753357605 1.814431645
C 2.0940144596 0.0221327489 0.7341164531
N 1.2746794042 -0.051615827 -0.3832350911
S -1.4927979478 0.0597355499 2.283657572
O -2.494713169 -0.3667980399 1.3740855364
N -1.57428151 1.6566489449 2.5408575653
O -1.1387159547 -0.6172274953 3.4772029354
H -0.867888163 -0.1038577844 -0.6565985727
H 1.5933449637 -0.1148331365 -1.3432459132
H 3.1681708353 0.0239112849 0.6618990473
H 1.483696831 0.1250767908 2.8691020604
H -2.4162296919 1.8436092433 3.1071429461

13
curtImN
1 1 -829.4698358 0.0878566 0 RB3LYP-D3/6-311+G(D,P)
N -0.000016024 -0.0034200302 0.0014070926
C 0.0016103657 -0.0009615066 1.3354908658
N 1.2831927638 0.0037015222 1.7248801933
C 2.1160771303 -0.012041159 0.6089178475
C 1.2977076751 -0.0086672521 -0.4753986119
N 1.6119010074 -0.1356023633 3.0684947569
S 2.6461099828 0.866973183 3.629508014
O 3.2824080741 1.8103547821 2.777921685
O 2.9324060781 0.6761924672 4.998153773
H -0.8493309045 -0.0184666073 1.9957754451
H -0.8368810539 -0.0055262641 -0.5690765892
H 1.527058589 -0.011395971 -1.527201585
H 3.1884991856 -0.016552645 0.6899824333

13
ImN_singlet
1 1 -829.3849264 0.0866559 0 RB3LYP-D3/6-311+G(D,P)
N 0.001032279 -0.1229225676 -0.0079795807
C -0.0432121994 -0.0953419701 1.3195355898
N 1.2186027889 0.0861280151 1.7503526288
C 2.080089337 0.1771466585 0.6565819773
C 1.3075893777 0.0431470957 -0.4496165513
S 1.6948216402 0.2043583311 3.4236788788
O 2.8415587091 -0.7274942243 3.5302690915
O 0.4670476028 0.1161821354 4.1076615199
N 2.8932322351 1.1410540917 3.5902431188
H -0.9131379811 -0.1974516135 1.9479180992
H -0.8098005247 -0.2464524972 -0.6043886834
H 1.5648931861 0.0527885624 -1.495198553
H 3.1387979107 0.330408558 0.7803096176

15
ImN3_noPC3
1 1 -938.9133647 0.0967743 0 RB3LYP-D3/6-311+G(D,P)
N -0.1540522163 -0.1938401356 -0.0781253794
C -0.0664678343 -0.0394619015 1.242328202
N 1.2364811989 -0.0454274455 1.5531244796
C 1.9946813301 -0.2102016419 0.400183649
C 1.1130513188 -0.3044821873 -0.6293795025
S 1.8725879927 0.1507387044 3.2328561954
O 3.1701227185 -0.4651978039 3.1702194698
O 0.7553038842 -0.2544440028 4.0301845354
N 1.954540273 1.8315198552 3.3198995874
N 3.1156225254 2.3055314017 3.1765647792
N 4.0801399085 2.8647359391 3.0860177276
H -0.8794052316 0.0611897443 1.9433714029
H -1.0258536312 -0.2385440583 -0.5929307865
H 1.272172187 -0.450177467 -1.6841761033
H 3.0690959864 -0.2739378028 0.4279761903

6 NMR spectra of the synthetized compounds

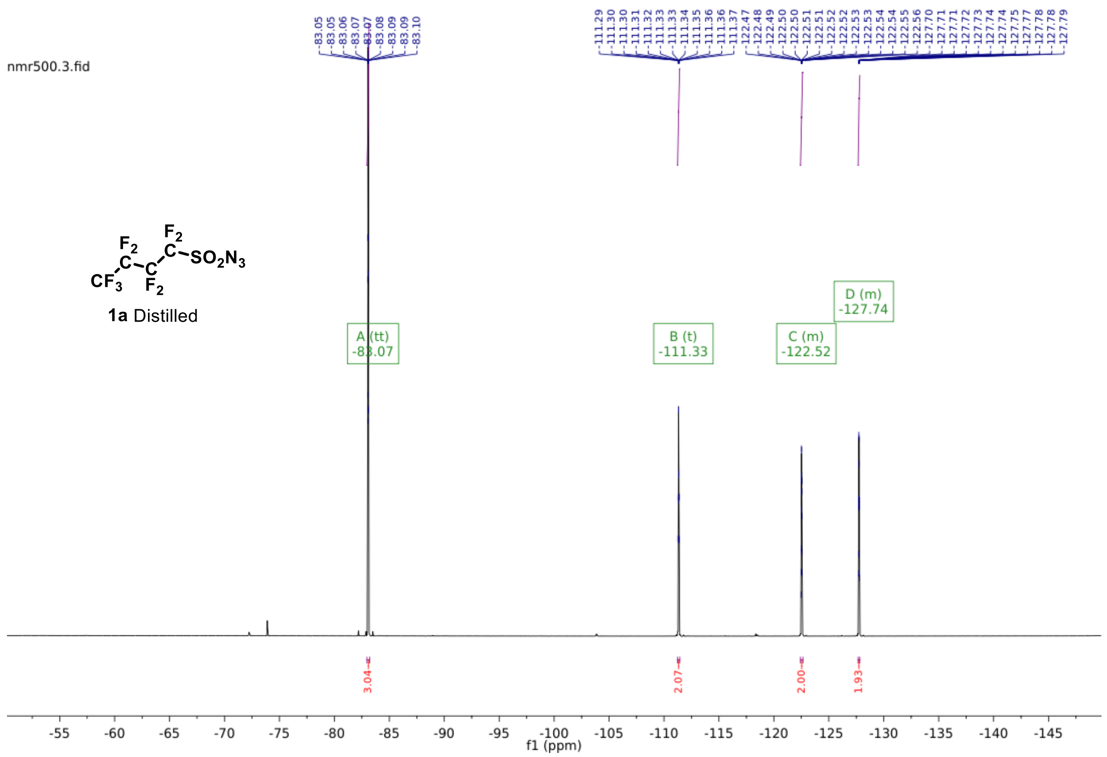

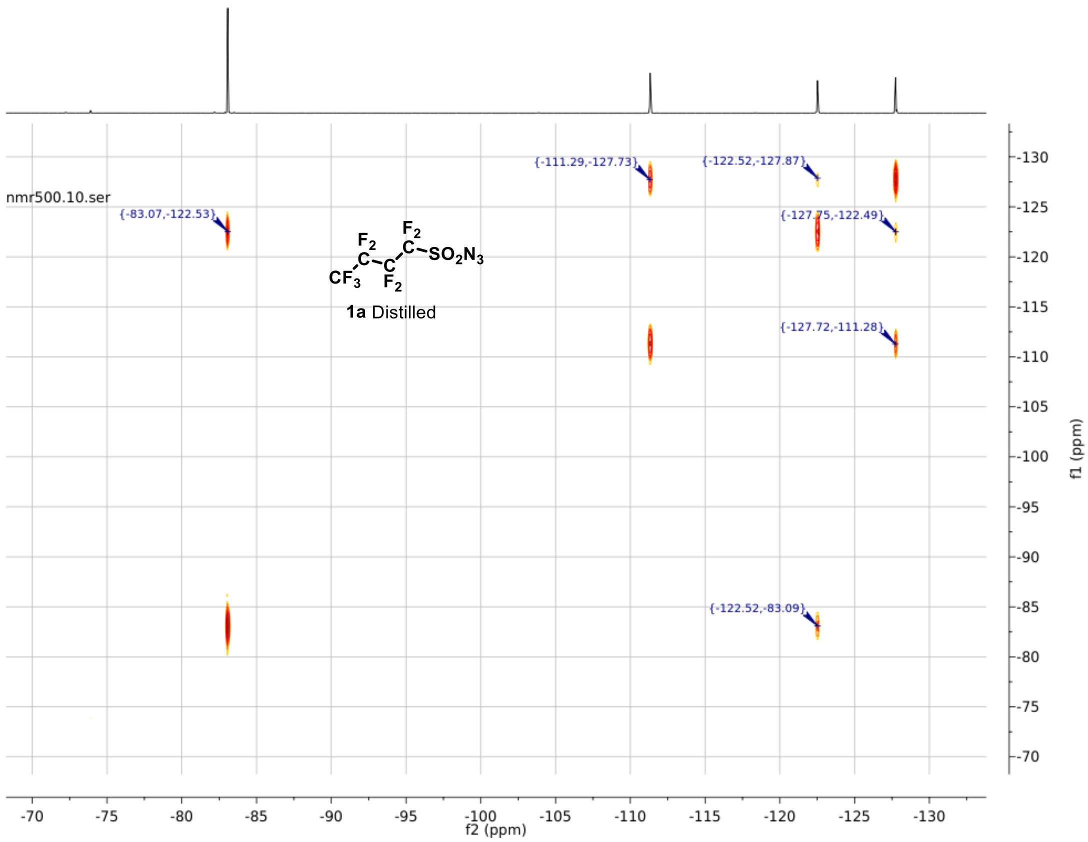

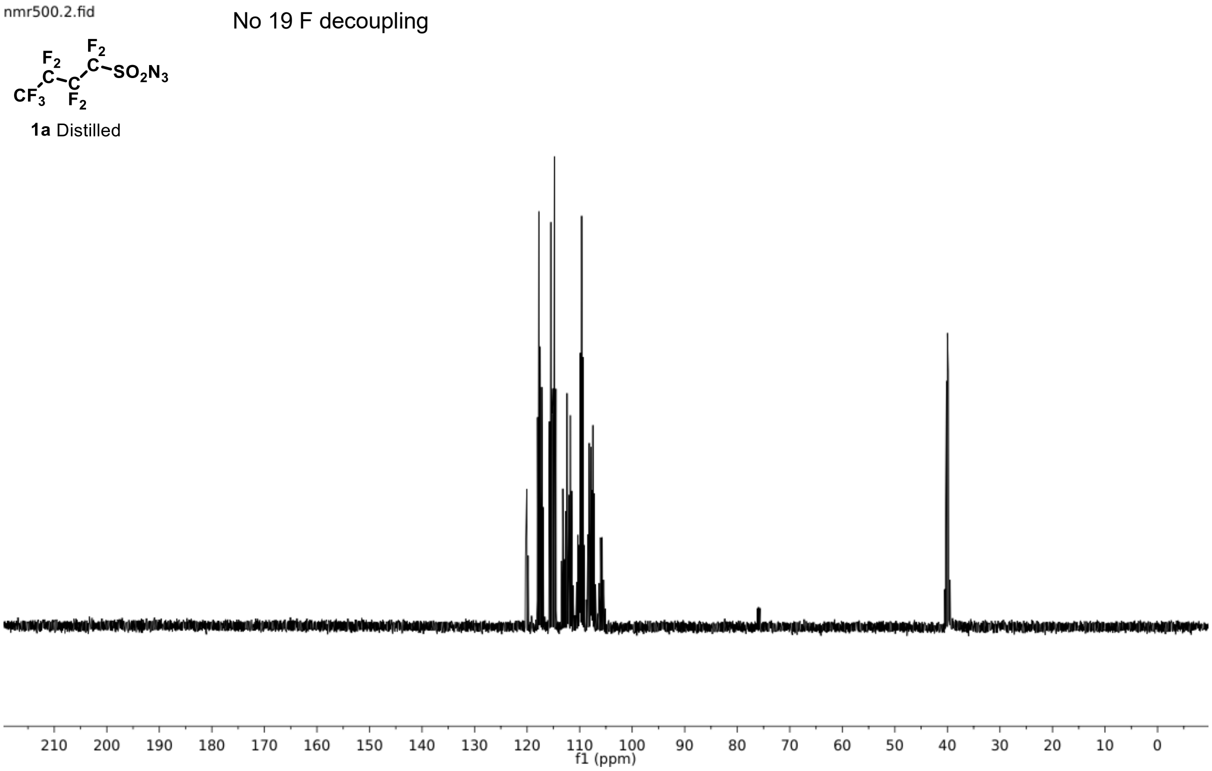
nmr500.2.fid
No 19 F decoupling
1a Distilled
210 200 190 180 170 160 150 140 130 120 110 100 90 80 70 60 50 40 30 20 10 0
f1 (ppm)

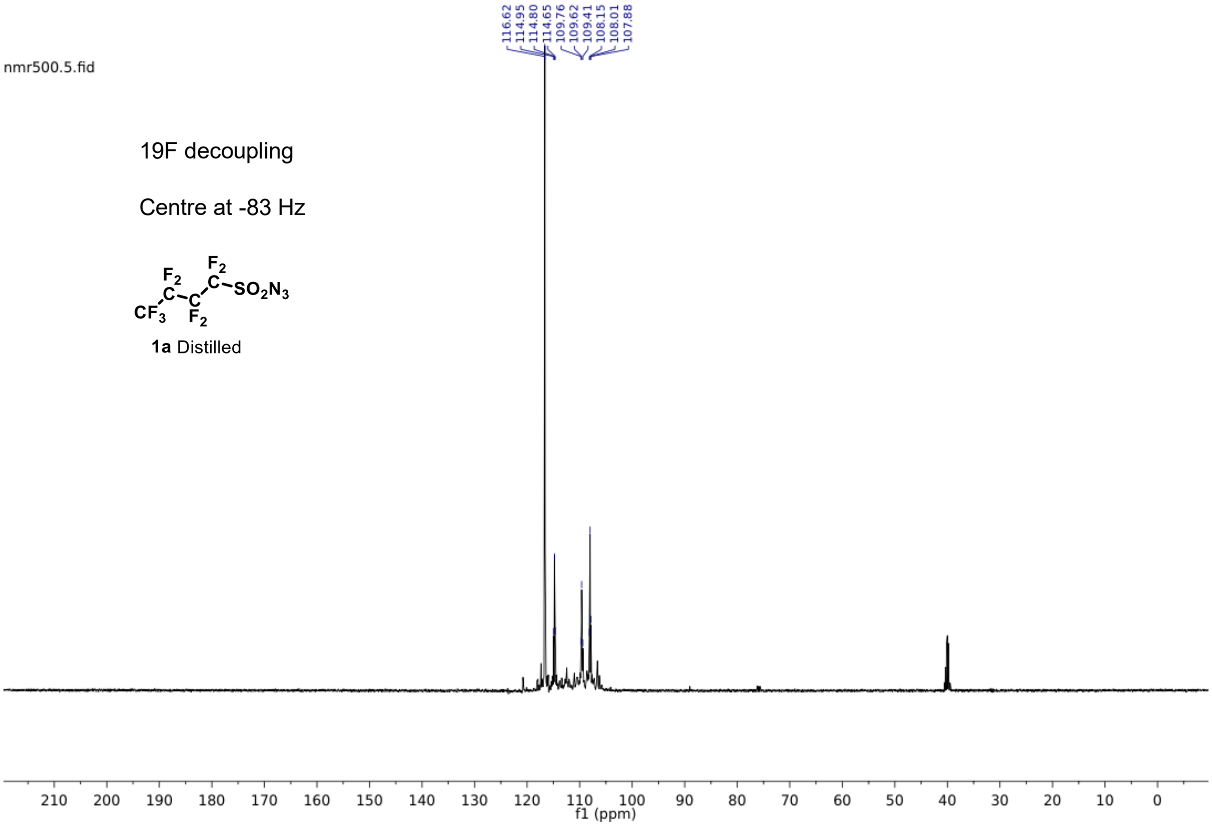
116.62
114.95
114.80
114.65
109.76
109.62
109.41
108.15
108.01
107.88
nmr500.5.fid
19F decoupling
Centre at -83 Hz
1a Distilled
210 200 190 180 170 160 150 140 130 120 110 100 90 80 70 60 50 40 30 20 10 0
f1 (ppm)

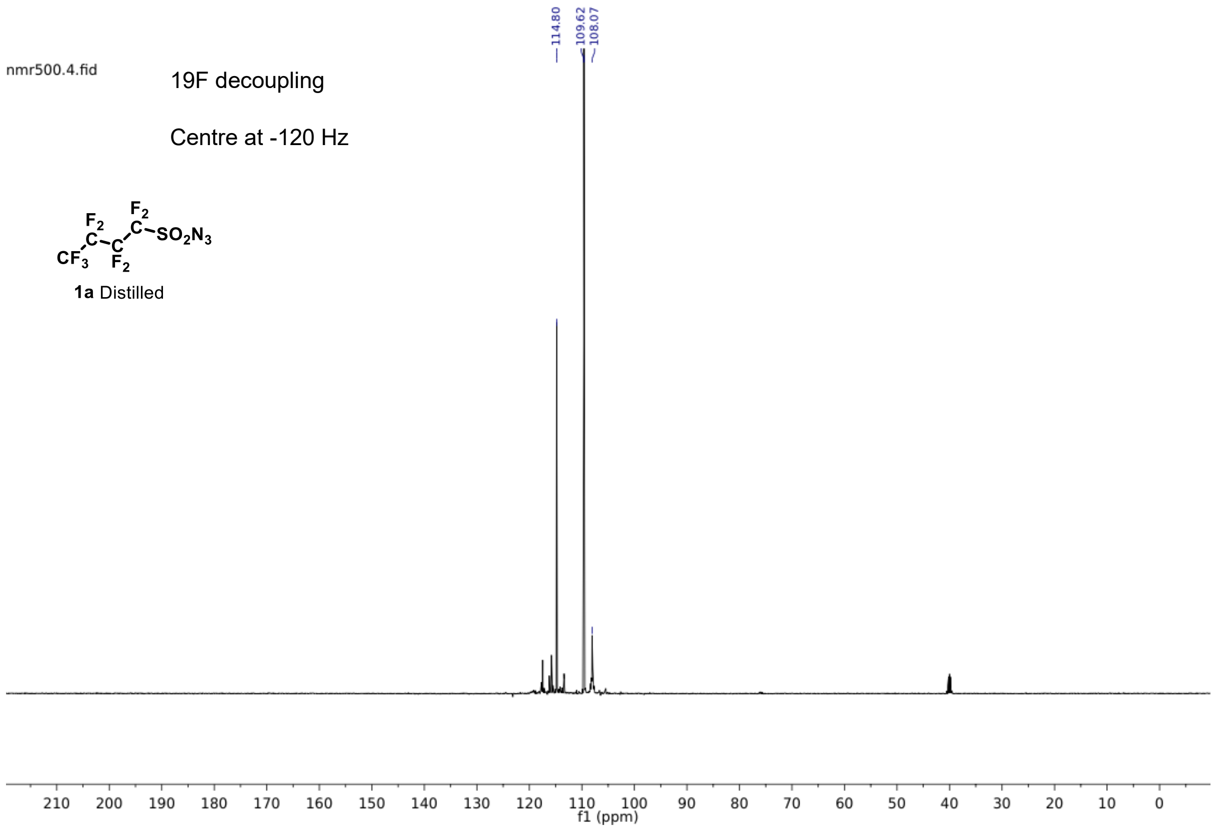
nmr500.4.fid
19F decoupling
Centre at -120 Hz
1a Distilled
114.80
109.62
108.07
f1 (ppm)

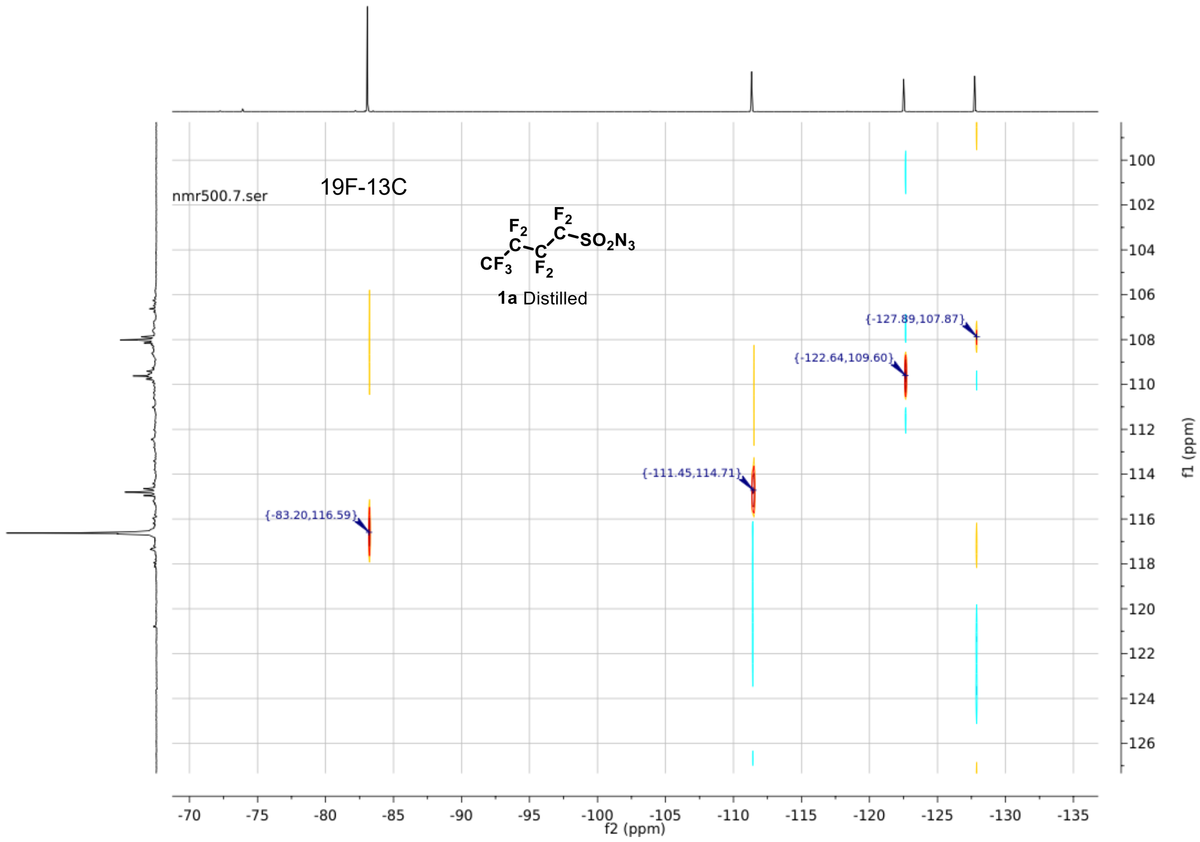
nmr500.7.ser
19F-13C
1a Distilled
{-127.89,107.87}
{-122.64,109.60}
{-111.45,114.71}
{-83.20,116.59}
f2 (ppm)
f1 (ppm)

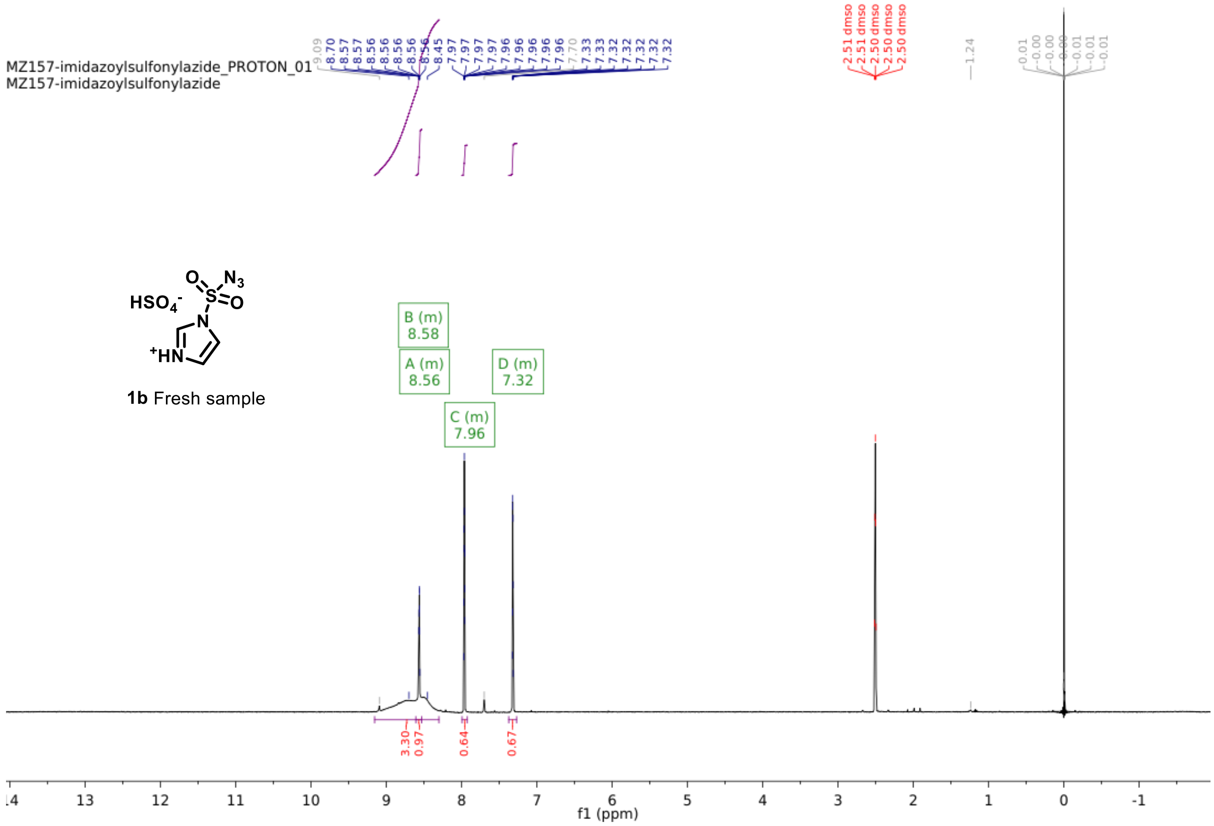
MZ157-imidazoylsulfonylazide_PROTON_01
MZ157-imidazoylsulfonylazide
HSO4-
1b Fresh sample
B (m) 8.58
A (m) 8.56
D (m) 7.32
C (m) 7.96
2.51 dmso
2.50 dmso
1.24
3.30
0.97
0.64
0.67
f1 (ppm)

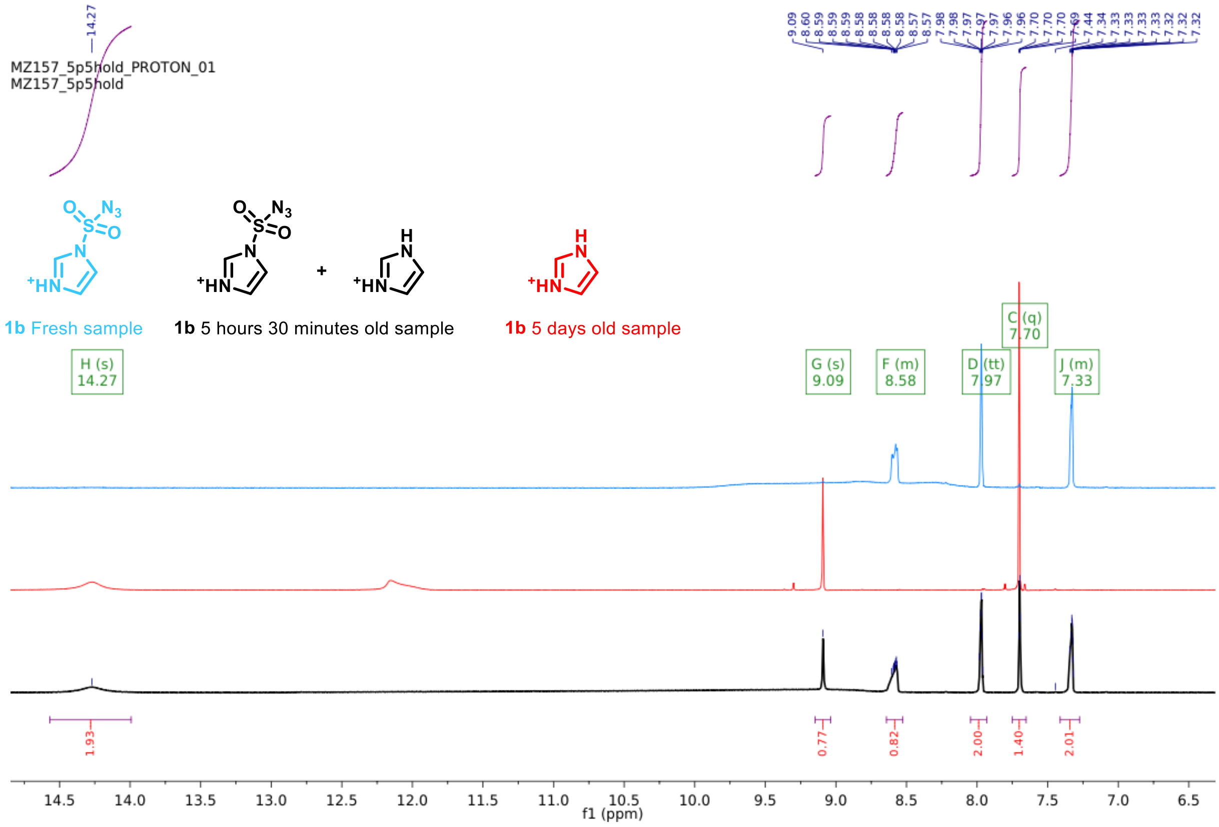
MZ157_5p5hold_PROTON_01
MZ157_5p5hold
14.27
1b Fresh sample
1b 5 hours 30 minutes old sample
1b 5 days old sample
H (s) 14.27
G (s) 9.09
F (m) 8.58
D (tt) 7.97
C (q) 7.70
J (m) 7.33
1.93
0.77
0.82
2.00
1.40
2.01
f1 (ppm)

[1] For synthesis of Nonaflyl azide: a) S.-Z. Zhu*, J. Chem. Soc., Perkin Trans. 1* **1994**, 2077–2081; b) B. Trastoy, M. E. Pérez-Ojeda, R. Sastre, J. L. Chiara, *Chemistry – A European Journal* **2010**, *16*, 3833–3841; For synthesis of 3-azidosulfonyl-3H-imidazol-1-ium Hydrogen Sulfate: c) G. T. Potter, G. C. Jayson, G. J. Miller, J. M. Gardiner, *J. Org. Chem.* **2016**, *81*, 3443–3446.

[2] D. Hernández-Guerra, A. Hlavačková, C. Pramthaisong, I. Vespoli, R. Pohl, T. Slanina, U. Jahn, Angew. Chem. Int. Ed. 2019, 131, 12570–12575.

[3] N. Fischer, E. D. Goddard-Borger, R. Greiner, T. M. Klapötke, B. W. Skelton, J. Stierstorfer, *J. Org. Chem.* **2012**, *77*, 1760–1764.

[4] a) J. Zelenka, R. Cibulka, J. Roithová, *Angew. Chem. Int. Ed.* **2019**, *58*, 15412–15420; for review see: b) J. Zelenka, J. Roithová, *ChemBioChem* **2020**, n/a, DOI 10.1002/cbic.202000072.

[5] a) K. L. Vikse, M. P. Woods, J. S. McIndoe, *Organometallics* **2010**, *29*, 6615–6618; b) K. L. Vikse, Z. Ahmadi, J. Luo, N. van der Wal, K. Daze, N. Taylor, J. S. McIndoe, *Int. J. Mass Spectrom*. **2012**, *323–324*, 8–13; c) X. Yan, E. Sokol, X. Li, G. Li, S. Xu, R. G. Cooks, *Angew. Chem. Int. Ed.* **2014**, *53*, 5931–5935.

[6] For instrument description please refer to: J. Jašík, J. Žabka, J. Roithová, D. Gerlich, *Int. J. Mass Spectrom*. **2013**

[7] For ion spectroscopy please refer to: a) High-resolution spectroscopy of cluster ions E. J. Bieske, O. Dopfer, *Chem. Rev.* **2000**, *100*, 3963–3998; b) Gas-phase infrared multiple photon dissociation spectroscopy of mass-selected molecular ions J. Oomens, B. G. Sartakov, G. Meijer, G. Von Helden, *Int. J. Mass Spectrom.* **2006**, *254*, 1–19; c) Infrared spectroscopy of organometallic ions in the gas phase: From model to real world complexes L. MacAleese, P. Maitre, *Mass Spectrom. Rev.* **2007**, *26*, 583–605; d) Characterization of reaction intermediates by ion spectroscopy J. Roithova, *Chem. Soc. Rev.* **2012**, *41*, 547–559; e) Cryogenic Ion Chemistry and Spectroscopy A. B. Wolk, C. M. Leavitt, E. Garand, M. A. Johnson, *Accounts Chem. Res.* **2014**, *47*, 202–210; f) Cryogenic ion trap vibrational spectroscopy of hydrogen-bonded clusters relevant to atmospheric chemistry N. Heine, K. R. Asmis, *Int. Rev. Phys. Chem.* **2015**, *34*, 1–34; g) A. M. Rijs, J. Oomens, in *Gas-Phase IR Spectroscopy and Structure of Biological Molecules*, Springer International Publishing, Cham, **2015**, pp. 1–42; h) Helium Tagging Infrared Photodissociation Spectroscopy of Reactive Ions J. Roithova, A. Gray, E. Andris, J. Jasik, D. Gerlich, *Accounts Chem. Res.* **2016**, *49*, 223–230; i) Infrared spectroscopy of cold trapped molecular ions using He-tagging D. Gerlich, *J. Chin. Chem. Soc.* **2018**, *65*, 637–653; j) Infrared Multiphoton Dissociation Spectroscopy with Free-Electron Lasers: On the Road from Small Molecules to Biomolecules L. Jasikova, J. Roithova, *Chem.-Eur. J.* **2018**, *24*, 3374–3390; k) Identification of Active Sites and Structural Characterization of Reactive Ionic Intermediates by Cryogenic Ion Trap Vibrational Spectroscopy H. Schwarz, K. R. Asmis, *Chem.: Eur. J.* **2019**, *25*, 2112–2126; l) Infrared ion spectroscopy: New opportunities for small-molecule identification in mass spectrometry - A tutorial perspective J. Martens, R. E. van Outersterp, R. J. Vreeken, F. Cuyckens, K. L. M. Coene, U. F. Engelke, L. A. J. Kluijtmans, R. A. Wevers, L. M. C. Buydens, B. Redlich, et al., *Anal. Chim. Acta* **2020**, *1093*, 1–15.

[8] a) A. D. Becke, *J. Chem. Phys.* **1993**, *98*, 5648–5652; b) C. Lee, W. Yang, R. G. Parr, *Phys. Rev. B* **1988**, *37*, 785–789.

[9] Effect of the damping function in dispersion corrected density functional theory, S. Grimme, S. Ehrlich, L. Goerigk, *J. Comp. Chem.* **2011**, *32*, 1456-1465.

[10] For pc-3 basis set refer to: F. Jensen, T. Helgaker, *J. Chem. Phys.* **2004**, *121*, 3463–3470; for basis set exchange refer to: B. P. Pritchard, D. Altarawy, B. Didier, T. D. Gibson, T. L. Windus, *J. Chem. Inf. Model.* **2019**, *59*, 4814–4820.

[11] a) M. J. S. Dewar, W. Thiel, *J. Am. Chem. Soc.* **1977**, *99*, 4899–4907; b) M. J. S. Dewar, E. G. Zoebisch, E. F. Healy, J. J. P. Stewart, *J. Am. Chem. Soc.* **1985**, 107, 3902–3909; c) M. J. S. Dewar, C. H. Reynolds, *J. Comput. Chem.* **1986**, *7*, 140–143; d) M. J. S. Dewar, Y. C. Yuan, *Inorg. Chem.* **1990**, *29*, 3881–3890.

[12] S. Grimme, *J. Comput. Chem*. **2006**, *27*, 1787–1799.

[13] For details please refer to SI. For B3LYP functional refer to: a) A. D. Becke, *J. Chem. Phys.* **1993**, *98*, 5648–5652; b) C. Lee, W. Yang, R. G. Parr, *Phys. Rev. B* **1988**, *37*, 785–789; For GD3-BJ dispersion refer to:S. Grimme, S. Ehrlich, L. Goerigk, *J. Comp. Chem.* **2011**, *32*, 1456-1465; For pc-3 basis set refer to: F. Jensen, T. Helgaker, *J. Chem. Phys.* **2004**, *121*, 3463–3470; for basis set exchange refer to: B. P. Pritchard, D. Altarawy, B. Didier, T. D. Gibson, T. L. Windus, *J. Chem. Inf. Model.* **2019**, *59*, 4814–4820.

[14] C. R. Bock, T. J. Meyer, D. G. Whitten, *J. Am. Chem. Soc.* **1975**, *97*, 2909–2911.

[15] For conversion between SCE, SSCE and Ag/Ag+ electrode we used coefficients obtained from: a) E. P. Friis, J. E. T. Andersen, L. L. Madsen, N. Bonander, P. Møller, J. Ulstrup, *Electrochim. Acta* **1998**, *43*, 1114–1122 and c) A. J. Bard, L. R. Faulkner, *Electrochemical Methods: Fundamentals and Applications*, Wiley, New York, **2001**; For conversion table, refer to: http://www.consultrsr.net/resources/ref/refpotls.htm
